# NBO/NRT Two-State Theory of Bond-Shift Spectral Excitation

**DOI:** 10.3390/molecules25184052

**Published:** 2020-09-04

**Authors:** Yinchun Jiao, Frank Weinhold

**Affiliations:** 1 Key Laboratory of Theoretical Organic Chemistry and Functional Molecules, Ministry of Education, School of Chemistry and Chemical Engineering, Hunan University of Science and Technology, Xiangtan 411201, China; yinchunjiao@hnust.edu.cn; 2 Theoretical Chemistry Institute and Department of Chemistry, University of Wisconsin-Madison, Madison, WI 53706, USA

**Keywords:** spectroscopy, reactivity, bonding analysis, natural bond orbitals, resonance theory

## Abstract

We show that natural bond orbital (NBO) and natural resonance theory (NRT) analysis methods provide both optimized Lewis-structural bonding descriptors for ground-state electronic properties as well as suitable building blocks for idealized “diabatic” two-state models of the associated spectroscopic excitations. Specifically, in the framework of single-determinant Hartree-Fock or density functional methods for a resonance-stabilized molecule or supramolecular complex, we employ NBO/NRT descriptors of the ground-state determinant to develop a qualitative picture of the associated charge-transfer excitation that dominates the valence region of the electronic spectrum. We illustrate the procedure for the elementary bond shifts of S_N_2-type halide exchange reaction as well as the more complex bond shifts in a series of conjugated cyanine dyes. In each case, we show how NBO-based descriptors of resonance-type 3-center, 4-electron (3c/4e) interactions provide simple estimates of spectroscopic excitation energy, bond orders, and other vibronic details of the excited-state PES that anticipate important features of the full multi-configuration description. The deep 3c/4e connections to measurable spectral properties also provide evidence for NBO-based estimates of ground-state donor-acceptor stabilization energies (sometimes criticized as “too large” compared to alternative analysis methods) that are also found to be of proper magnitude to provide useful estimates of excitation energies and structure-dependent spectral shifts.

## 1. Introduction

Chemical reactivity of a molecular or supramolecular species is often associated with characteristic features of its electronic spectroscopy [1]. It is noteworthy that G.N. Lewis, the founder of e-pair chemical bonding concepts, also played a pioneering role in studies of excited states [2,3,4] that underlie modern understanding of photochemistry [5,6,7,8,9]. A comprehensive theoretical conception of chemical bonding and reactivity should therefore aim to further elucidate the relationships that interconnect the reactive landscape of the ground state potential energy surface (PES) with those of spectroscopically connected excited states.

**Figure 1:**
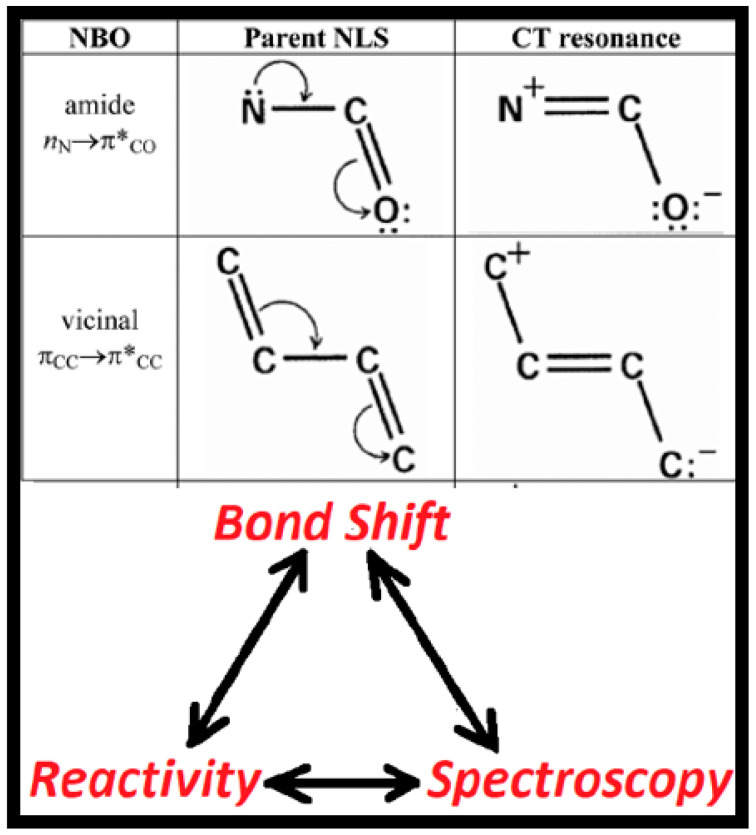
Three symbolic depictions of e-pair bond shifts (upper panels), seen as the key conceptual linkage between reactive and spectroscopic properties of the ground-state bonding pattern (see text).

As depicted in Figure 1, the central concept that directly links chemical reactivity and spectroscopy is the electron-pair bond shift. The upper portion of the figure represents well-known *e*-pair bond shifts of amides or polyenes in three alternative symbolic forms (from left to right in the upper row):

(1) Natural bond orbital (NBO) [10,11] donor-acceptor interaction that transfers two electrons from a Lewis-type (L; formally occupied) NBO of the parent natural Lewis-structure (NLS) bonding pattern (e.g., amide nitrogen lone pair, n_N_) to a non-Lewis (NL; formally vacant) NBO (e.g., adjacent carbonyl pi-antibond, π*_CO_);
(2) Robinson-type “curly arrow” depiction [12] of vicinal π-type delocalization, leading to π_CO_ → π_CN_ bond shift, with concomitant n_n _→ n_O_ lone pair shift and charge transfer;
(3) Resonance-structural depiction of the secondary bonding pattern that results from the e-pair bond shift in the parent NLS.

Resonance-type aspects (ii), (iii) of electronic bond shifts can be further quantified with the descriptors of natural resonance theory (NRT) [13,14,15], particularly the fractional bond orders {*b*_AB_} and resonance weightings {*w***_R_**} that allow continuous tracing of a bond shift from one limit to another along a reactive pathway. Such resonance-type NBO/NRT bond shifts of *n*_N_-π*_CO_ or π_CC_-π*_CC_ type are well-known to profoundly influence the structural, reactive, and spectroscopic properties of amides or polyenes, respectively.

The attempt to find simple connections between ground- and excited-state resonance bond-shifting bears a close relationship to the recently developed NBO/NRT picture of pseudo Jahn-Teller (PJT) effects [16]. Conventionally, PJT-based studies of vibronic symmetry-breaking [17] require examination of the full electronic excitation spectrum to identify a specific electronic root of the presumed symmetric precursor that can couple with the specific vibrational distortion mode leading to symmetry-breaking. This specific root (e.g., of a computed TD [18] spectrum) can then be taken as “perturbing state” for a 2nd-order (two-state) perturbative model that “explains” the symmetry-breaking. However, the envisioned electronic responses to a distortion of nuclear geometry are also quantified by the NBO/NRT-based descriptors of the ground-state PES, where the actual symmetry-breaking effect is observed. Simple $DEL-deletion techniques (as described below) thereby allow a more incisive and complete “two-state” picture of vibronic symmetry-breaking effects to be constructed purely from NBO/NRT descriptors of the electronic ground-state PES, in close parallel to the logic and methods of the present work.

**Figure 2:**
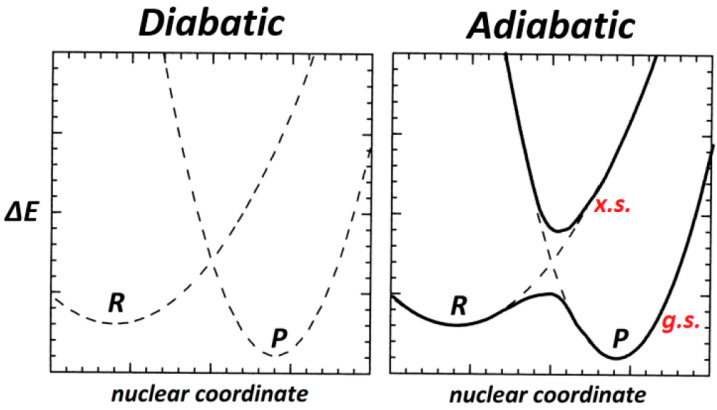
Schematic two-state coupling model of “diabatic” (localized bond) potential curves (centered at distinct ***R***, ***P*** positions along a reactive pathway) that interact vibronically to give final “adiabatic” potential curves for ground (***g.s.***) and excited (***x.s.***) spectroscopic states.

If we identify the parent and secondary bonding patterns of Figure 1 as **I**, **II**, respectively, we can envision using these labels to identify a variety of resonance-structural, reactivity, spectroscopic, wavefunction, or *e*–configuration descriptors, as shown in Table 1:

The essence of an envisioned “two-state” relationship between bonding patterns of a ground-state (***g.s.***) PES and those of an associated excited-state (***x.s.***) is conventionally visualized in terms of two *diabatic* potential curves, each representing the stretching of an idealized (non-interacting) localized bond oscillator centered at distinct positions (***R*** or ***P***) along a reactive nuclear coordinate pathway, as shown in the left panel of Figure 2. The vibronic couplings between the ***R***-centered vs. ***P***-centered bonding potentials then lead to the actual *adiabatic* ***g.s.*** and ***x.s.*** potential curves, as depicted in the right panel of Figure 2.

The assumed close relationship between spectroscopy and chemical bonding interactions underlies Mulliken’s well-known theory of charge transfer (CT) complexes [19]. Mulliken recognized that the sharp color change of a complex formed from apolar, closed-shell components (e.g., I_2_ + benzene) demands a non-classical interaction of chemical bonding type. In a simple two-state model, such bonding attraction can be expressed as a two-term superposition of ionic and covalent-bonding (“diabatic precursor”) configurations. In the presence of resonance-type configurational mixing effects, the idealized diabatic precursors undergo avoided crossing to yield final (adiabatic) states whose spectroscopic energy difference can be empirically related to the strength of mixing coefficients. Mirror-type symmetries and two-state character of couplings between ground- and excited-state potentials in the neighborhood of avoided crossings were also extensively explored in the framework of valence bond (VB)-based state correlation diagram (VBSCD) theory by Shaik and coworkers [20,21,22,23,24,25]. Analogous two-state conceptions can also be recognized in the Marcus two-parabola picture of electron transfer reactions [26].

More generally, the concept of CT-type covalent-ionic resonance was recognized by Coulson [27] as the essence of the Pimentel 3-center, 4-electron (3c/4e) model of hydrogen bonding and related hyperbonding phenomena [28], and such “chemical bonding” aspects of H-bond formation are now widely recognized in the research literature [29] (if not yet in elementary textbooks [30]). In addition, many faithful analogies can be demonstrated [31] between H-bonding and other so-called noncovalent interactions (pnicogen bonds [32,33], halogen bonds [34,35,36], aerogen bonds [37,38], and so forth [39,40]), leading to the inference that resonance-type 3c/4e interactions are the essential “glue” of practically all supramolecular complexation phenomena of chemical interest. Thus, such resonance-type two-state mixing underlies important aspects of molecular and supramolecular *structure*, as well as the broader *reactivity* and *spectroscopy* aspects addressed in the present work.

Still more generally, recognition of the importance of resonance-type 3c/4e bonding in the supramolecular domain merely represents the sub-integer extension of resonance-type fractional bonding effects that are well known in the supra-integer bonding domain of conjugated molecules [41]. Although such resonance effects are still criticized in some quarters as a figment of chemical imagination (“unicorns” [42]), the accumulating weight of chemical evidence indicates their essential role in an ever-expanding array of chemical phenomena. The present work aims to further extend this integrated resonance-based picture of chemical structure, reactivity, and spectroscopy.

The plan of our paper is as follows: We first briefly review the working tools of NBO/NRT analysis that allow variational bond orders and other resonance-type descriptors to be extracted from wavefunctions or densities of any form or accuracy. Starting from the simplest form of two-state model in the framework of single-determinant Kohn-Sham density functional theory (KS-DFT) [43], we show how NBO-based deletion techniques [44] can be used to construct suitable diabatic models for the two-state secular determinant that couples KS-DFT description of the ground state to that of a target bond-shifted “mirror state” in the excitation spectrum. We apply the resulting two-state KS-DFT spectroscopic model to two simple illustrative cases: (i) degenerate S_N_2 fluoride exchange reaction (F^−^ + CH_3_-F → F-CH_3_ + F^−^), (ii) CT excitations in a series of cyanine dyes [H_2_N(CH)_2n+1_NH_2_^+^, *n* = 2–5]. In each case we compare the simple two-state model with full multi-configurational time-dependent (TD) KS-DFT description to assess the accuracy and conceptual usefulness of the simplified spectroscopic picture, which adds negligibly to the computational cost of conventional ground-state KS-DFT calculation. A summary of our results and the prospects for further multi-state extension of the NBO/NRT spectroscopic model are discussed in the concluding section.

## 2. NBO/NRT Deletion Tools for Describing Resonance Delocalization

The principal objective of default NBO analysis is to find the best possible natural Lewis structure (NLS) orbitals {Ω_i_^(L)^} for a determinantal wavefunction Ψ^(L)^ of idealized Lewis-structural form,

Ψ^(NLS)^ = det|(Ω_1_^(L)^)^2^(Ω_2_^(L)^)^2^…(Ω_N/2_^(L)^)^2^| (1)

where each Ω_i_^(L)^ corresponds to a 1-center (lone pair) or 2-center (bond) feature of the optimal bonding pattern. The Lewis-type {Ω_i_^(L)^} are complemented by non-Lewis {Ω_j_^(NL)^} NBOs that complete the full orthonormal span of the starting basis set. The non-Lewis NBOs allow more complete description of donor-acceptor (Ω_i_^(L)^)^2^ → (Ω_j_^(NL)^)^2^ (2-electron stabilizing [44] (please see fig 5.1 in reference [44])) effects in the full multi-determinant Ψ. For notational convenience, we denote each such (Ω_i_^(L)^)^2^ → (Ω_j_^(NL)^)^2^ delocalization as the “i→j*” correction to the idealized Ψ^(NLS)^, with associated energy lowering Δ*E*_i__→j*_. Each i→j* interaction can also be associated with a corresponding resonance-structural bonding pattern (Figure 1) whose weighting (*w*_i__→j*_) can be quantified by NRT analysis [13,14,15].

In Hartree-Fock (HF) or Kohn-Sham (KS) level, the resonance-type energy lowering Δ*E*_i__→j*_ can be estimated by the simple 2nd-order perturbation theory expression [44] (please see fig 5.1 in reference [44]),

Δ*E*_i__→j*_^(2)^ = −|F_i,j*_|^2^/(ε_j*_ − ε_i_) (2)

where F_i,j*_ is the off-diagonal element connecting diagonal elements ε_j*_, ε_i_ of the HF/KS 1-electron (1e) effective Hamiltonian **F**-matrix in NBO representation. However, an alternative (quasi-variational) estimate of Δ*E*_i→j*_ is obtained by formally “deleting” the i→j* interaction (with $DEL keylist input [44]) and recalculating the variationally raised energy (*E*_i__→j*_^($DEL)^) when such delocalization is absent, viz.

Δ*E*_i__→j*_^($DEL)^ = *E*_full_ − *E*_i__→j*_^($DEL)^ (3)

In principle. such $DEL-type evaluations could be carried out at any theory level [i.e., by removing (Ω_i_^(L)^)^2^ → (Ω_j_^(NL)^)^2^ double-substitutions from the full multi-determinant expansion and recalculating the variational energy], but in practice the required 4-index transformation to the NBO basis is computationally intensive. In contrast, such numerical *E*_i__→j*_^($DEL)^ evaluations are virtually cost-free in HF/KS theory, so we restrict attention to the simple B3LYP/6-311++G** level of hybrid-DFT theory [45] in the ensuing applications.

## 3. Diabatic Two-State Model of Resonance Mixing

The most extreme $DEL-deletion type (of the nine keylist options in the current *NBO 7.0* program [46,47]) is to delete *all* delocalizations from the parent NLS bonding pattern, corresponding to the (strictly variational) energy of the idealized Ψ^(NLS)^ single determinant, Equation (1). For a simple two-state model, we may start from the determinants Ψ^(NLS−1)^, Ψ^(NLS−2)^ for two distinct resonance-structural bonding patterns, with associated diabatic energies H_11_, H_22_ [diagonal elements of system Hamiltonian *ℋ* = *ℋ*(***R***) at fixed nuclear geometry ***R***],

H_11_ = *E*^(NLS−1)^ = 〈Ψ^(NLS−1)^|*ℋ*|Ψ^(NLS−1)^〉 (4)

H_22_ = *E*^(NLS−2)^ = 〈Ψ^(NLS−2)^|*ℋ*|Ψ^(NLS−2)^〉 (5)

and off-diagonal interaction element H_12_,

H_12_ = 〈Ψ^(NLS−1)^|*ℋ*|Ψ^(NLS−2)^〉 (6)

As is well known [48], the simple 2 × 2 secular equation for this model can be written as

(7) $$\det\left[\begin{array}{ccc} H_{11}\;-ℇ & & H_{12}\\ H_{12} & & H_{22}\;-\;ℇ \end{array}\right]=0$$

with solutions ℇ_±_ given by

ℇ_±_ = ½(H_11_ + H_22_) ± δ (8)

δ = [¼(H_22_ − H_11_)^2^ + H_12_^2^]^½^ (9)

In the perturbative limit where H_22_ − H_11_ >> H_12_^2^, the low-energy (ground-state) and high-energy (excited-state) solutions can be approximated as

ℇ_g.s._ = ℇ_−_ ≈ H_11_ − H_12_^2^/(H_22_ − H_11_) (10)

ℇ_x.s._ = ℇ_+_ ≈ H_22_ + H_12_^2^/(H_22_ − H_11_) (11)

which corresponds to the 2nd-order perturbative estimate, Equation (2). However, the more general expressions (8), (9) remain strictly valid in the strong-coupling regime.

From Equations (8), (9) the exact ℇ_g.s._, ℇ_x.s_ solutions are evaluated as

ℇ_g.s._ = ℇ_−_ = ½(H_22_ + H_11_) − δ (12)

ℇ_x.s._ = ℇ_+_ = ½(H_22_ + H_11_) + δ (13)

From the $DEL-deletion calculations, we obtain both the diabatic-state energies (H_11_, H_22_) and the respective energy differences (δ_1_, δ_2_) by which each is separated (raised) from the starting adiabatic-state energy ℇ_g.s._

δ_1_ = H_11_ − ℇ_g.s._ = δ − ½(H_22_ − H_11_) (14)

δ_2_ = H_22_ − ℇ_g.s._ = (H_22_ − H_11_) + δ_1_ (15)

Combining Equations (12)–(15) we obtain

ℇ_x.s._ − ℇ_g.s._ = 2δ = δ_1_ + δ_2_ (16)

which yields the intuitive identification of splitting parameter δ with the arithmetic mean of deletion energies δ_1_, δ_2_,

δ = ½(δ_1_ + δ_2_) (17)

From Equation (9) the corresponding off-diagonal coupling element is

H_12_ = {[(δ_1_ + δ_2_)/2]^2^ − [(H_22_ − H_11_)/2]^2^}^½^ (18)

Furthermore, just as ℇ_g.s._ = H_11_ − δ_1_, Equation (14), one sees from Equations (15), (16) that

ℇ_x.s._ = H_22_ + δ_1_ (19)

which gives the most direct two-state prediction of ℇ_x.s._ from $DEL-deletion descriptors. Equation (19) completes specification of the NBO/NRT-based two-state model for a chosen resonance bond-shift and geometry on the KS-DFT ground-state PES.

Although Equations (4)–(6) express the matrix elements H_11_, H_22_, H_12_ in terms of extreme NLS-type deletions, the solutions (12)–(19) apply as well to more selective $DEL-deletion types, such as those of individual i→j* interactions, viz.,

H_11_ = *E*_i→j*_^($DEL)^ (20)

H_22_ = *E*_iʹ→jʹ*_^($DEL)^ (21)

where i, j* denote the donor (i) and acceptor (j*) NBOs of state “1” and i′, j′* those of state “2” in the two-state description. Since an individual *E*_i__→j*_^($DEL)^ refers to a weaker deletion energy (Δ*E*_i__→j*_^($DEL)^ = δ_i__→j*_) and simpler superposition mixing than the sum of all such interactions in the *E*^(NLS)^ deletions of (4), (5), the single-element $DEL-deletions of (20), (21) can target features of the low-energy excitation spectrum that are of principal chemical interest.

The essence of two-state character in the excitation spectrum is expressed by Equations (12), (13), (17)–(19). The elementary two-state predictions can be tested by comparing the r.h.s. of Equation (19) (obtained from conventional KS-DFT ground-state $DEL-deletion calculations) with multi-configuration TD-DFT description of the excitation spectrum. Although TD-DFT (CI-singles-type) description cannot be expected to achieve quantitative representation of the experimental excitation spectrum, it provides a useful first approximation to the qualitative landscape of low-lying spectral features that can be compared with the still simpler two-state model. Such comparisons will be carried out for numerical examples of reactive bond-shifting species in the following section.

Still a deeper aspect of the NBO/NRT-based two-state picture can be inferred from the expected bond-order conservation rules [49] of resonance-type 3c/4e interactions. In a general A⋯B−C ↔ A−B⋯C resonance triad (e.g., of allylic or H-bonding type), the fixed valency (*V*_B_) of the central B atom implies that the sum of *b*_AB_, *b*_BC_ bond orders should remain constant (“conserved”) as bond order shifts from *b*_AB_ to *b*_BC_ along a reactive pathway on the ground-state PES,

*b*_AB_^(g.s.)^ + *b*_BC_^(g.s.)^ ≈ constant (along a reactive pathway) (22)

A corollary of the two-state NBO/NRT picture is the complementary (mirror-image) symmetry of bonding on the coupled ℇ_g.s_, ℇ_x.s_ surfaces, such that increased A⋯B bonding on ℇ_g.s_ is correlated with reduced A⋯B bonding on ℇ_x.s_ (and vice versa). Such complementarity is expected to lead to approximate conservation rules of the form

*b*_AB_^(g.s.)^ + *b*_AB_^(x.s.)^ ≈ constant (upon excitation at given geometry) (23)

Familiar correlations [50,51,52] of bond order with bond length, bond energy, IR frequency, or other properties then imply similar mirror-type relationships between a variety of structural and spectroscopic properties of the two states. Such secondary mirror symmetries between ground and excited state bond orders will be examined in numerical applications to follow.

## 4. Chemical Applications 

### 4.1. Methods

The applications to be described below were all performed with the B3LYP hybrid density functional of Becke [53] for the ground state and associated time-dependent TD-B3LYP method for excited states [18,54], all employing Pople-style 6-311++G** basis set [45]. However, certain comparisons are included using ab initio HF (Hartree-Fock) for ground state and CIS (configuration interaction with all single excitations) for excited states, or with other popular DFT functionals: CAM-B3LYP of Handy and coworkers [55], M06 of Truhlar and coworkers [56], and wB97XD of Head-Gordon and coworkers [57] (all employing 6-311++G** basis). All calculations were performed with the *Gaussian-16* (G16) program system [58], using standard program options for optimized geometry (OPT), frequencies (FREQ), and intrinsic reaction coordinate (IRC) evaluations. Gaussian input files with keywords, geometrical coordinates, and other information for all IRC and stationary points are included in Supplementary Materials Information (SI).

NBO/NRT analysis and $DEL-deletion calculations were performed with the *NBO 7.0* program in interactive tandem (G16/NBO7) with host G16. Standard NBO keywords and keylists for $DEL-deletion evaluations are described in the online NBO Manual [59] and illustrated in the files included in SI. Visualizations of NBO orbital interactions were prepared with the *NBOPro7@Jmol* [60] utility program.

### 4.2. S_N_2 Fluoride Exchange Reaction

A simple example of chemical reactivity is provided by the S_N_2-type [61] degenerate exchange reaction of fluoride ion with methyl fluoride (cf. related VBSCD studies of Shaik and coworkers [20,21,22,23,24,25]),

F^−^ + CH_3_F → (FCH_3_F^−^)^‡^ → FCH_3_ + F^−^ (24)

**Figure 3:**
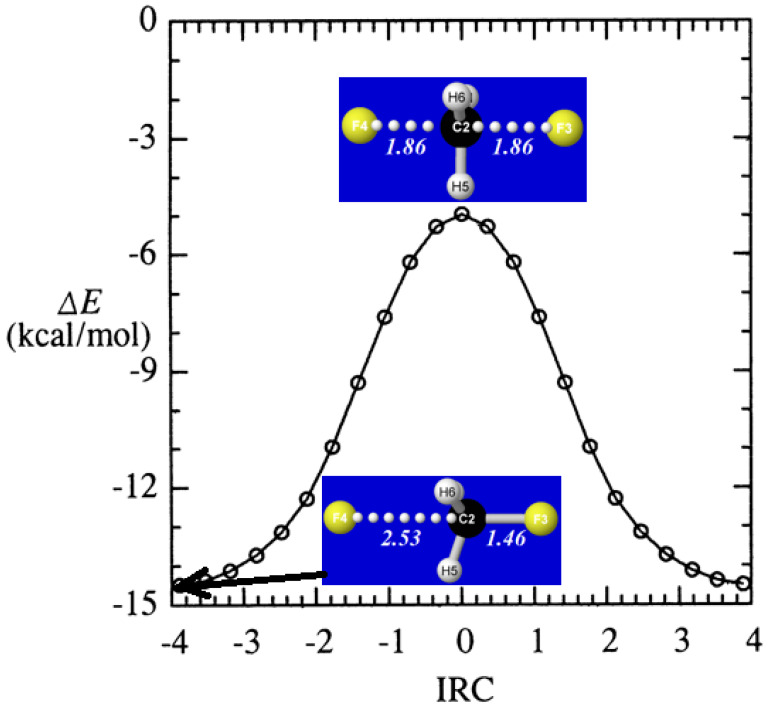
Energetic profile Δ*E* (relative to isolated F^−^ + CH_3_F) of S_N_2 halide displacement reaction (24) along the intrinsic reaction coordinate (IRC), with inserts showing the *C*_3v_-symmetric geometry of the long-range reactant complex (IRC = −3.89) and *D*_3h_ geometry of the transition state (IRC = 0).

From the starting *D*_3h_-symmetric transition state complex (FCH_3_F^−^)^‡^, we obtain the full intrinsic reaction coordinate (IRC) [62], which terminates at a stable long-range ion-molecule complex in either reactant or product direction, as shown in the energetic profile of Figure 3. The figure inserts show interatomic distances (Å) both in the long-range equilibrium complexes (near IRC = ±3.89) and *D*_3h_-symmetric transition state (IRC = 0) of the reaction pathway.

**Figure 4:**
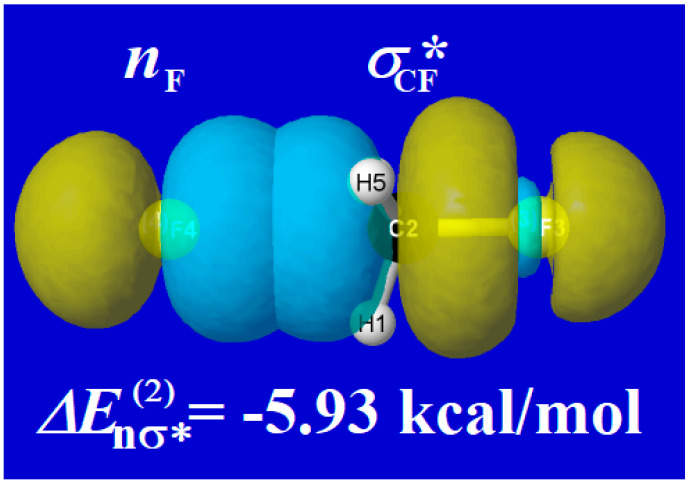
(Pre-)NBO overlap diagram for *n*_F_ → σ_CF_* interaction of F^−^∙∙∙CH_3_F in the long-range approach region (IRC = −3.89), with 2nd-order perturbation estimate of the associated resonance-type stabilization.

The key NBO donor-acceptor interaction is of *n*_F_ → σ_CF_* type, representing resonance-type delocalization (charge transfer) from the fluoride lone pair (*n*_F_) to the backside of the C–F valence antibond (σ_CF_*). This orbital interaction is illustrated in Figure 4 for the geometry of the reactant complex (IRC = −3.89; cf. Figure 3). At this geometry, the estimated 2nd-order stabilization energy is Δ*E*_nσ*_^(2)^ = −5.93 kcal/mol. 

**Figure 5:**
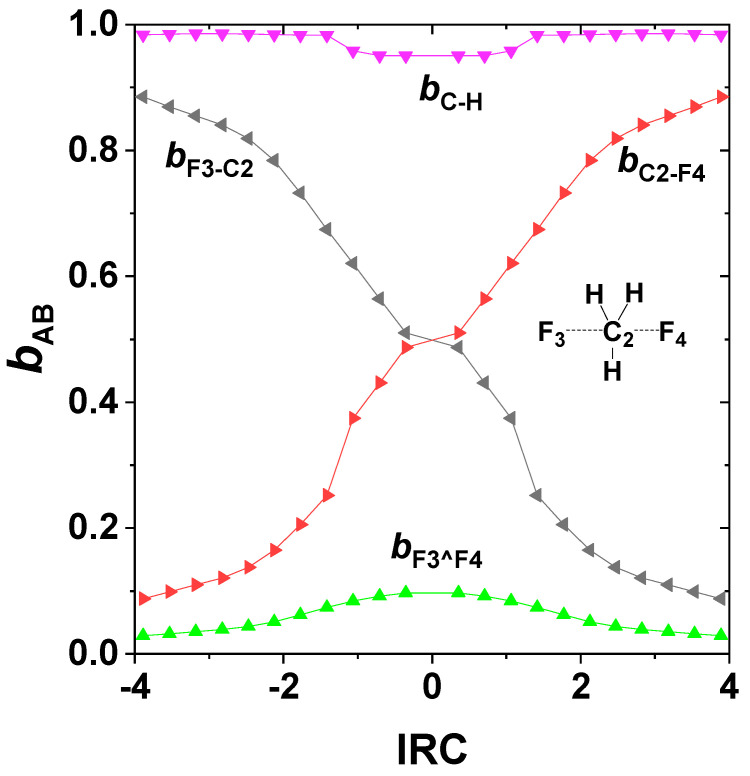
NRT bond orders of S_N_2 exchange reaction (24) along the IRC, with atom numberings as shown in the figure inset.

One can also evaluate the NRT bond orders along the IRC to visualize the continuous changes of *b*_CF_, *b*_CF’_, *b*_CH_ on the ground-state potential, as shown in Figure 5. The *b*_CH_ (violet) bond orders vary only slightly from unity near the transition state, whereas the complementary *b*_CF_, *b*_CF’_ (red, black) bond orders vary steeply between near-zero and near-unit values in reciprocal manner. Also shown are the *b*_F^F’_ (green) “long-bond” orders (a surprising form of 3c/4e interaction [63]), which contribute appreciably to stabilizing the transition-state region.

**Figure 6:**
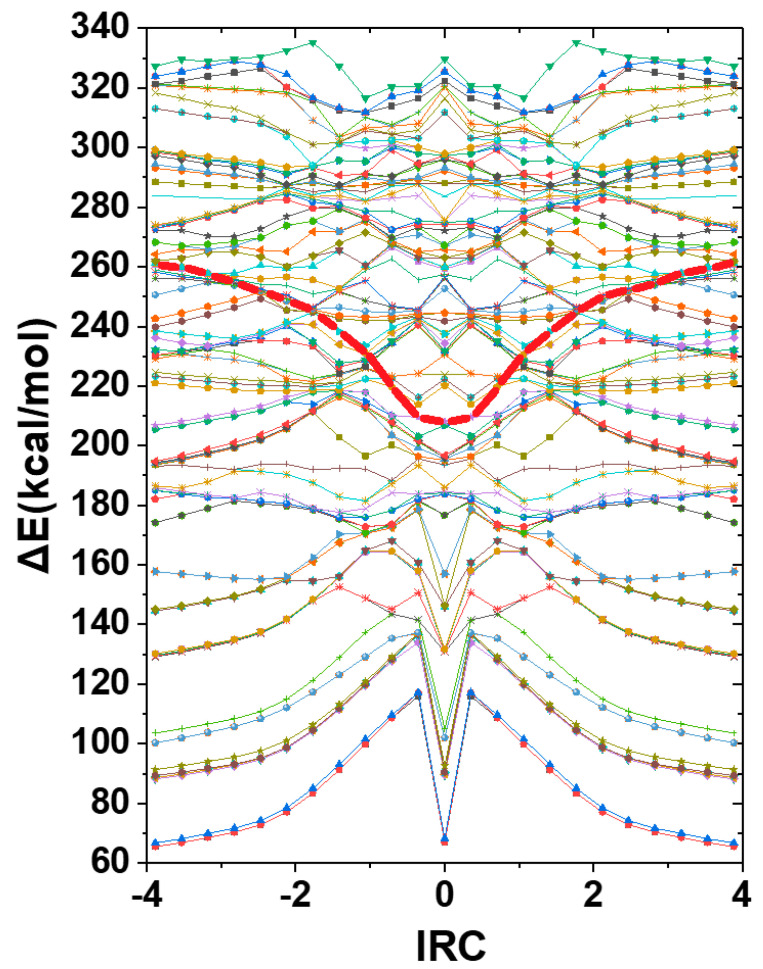
Excitation diagram for the 100 lowest TD roots along the IRC of methyl fluoride S_N_2 exchange reaction (24), showing (in dark-red connecting lines) the band of bright roots of maximal oscillator strength (sharply distinguishable from the many adjacent or interleaving dark roots of negligible intensity) that are taken as the spectroscopically significant “ℇ_x.s._^(TD)^ excited state” for present purposes. (See SI for TD root-numbers of connectedpoints along the bright spectral sequence.).

At any geometry along the computed IRC, the FCH_3_F^−^ species does not exhibit appreciable intensity (oscillator strength) in any of its low-lying TD excited states. Instead, a connected band of high-intensity (maximal oscillator strength) transitions is found among the higher roots of the TD spectrum, occasionally crossed by other dark roots of negligible intensity that alter the nominal root-number of the high-intensity feature. Figure 6 displays this complex structure of excited roots in the TD excitation spectrum, showing (in bold red) the connected bright roots of maximal oscillator strength (compared to nearby dark roots of negligible intensity) that are identified as the relevant spectral excitation root at each IRC value. The numerous dark roots surrounding each bright root of the anionic complex are interpreted as finite-basis representations of the background autoionization continuum states, whose details may be highly basis-dependent compared to the reasonably stable representation of the high-intensity valence-state feature. The sequence of connected bright roots in Figure 6 (each explicitly identified by TD root-number in SI) is adopted as the relevant excited state ℇ_x.s._^(TD)^ for NBO/NRT analysis and comparison with two-state predictions along the IRC. Note that color-connections based on TD root-number necessarily incur apparent “discontinuities” in slope near physical curve-crossings, whereas the red curve of maximal oscillator strength (connecting points of varying root-numbers) varies smoothly along the IRC.

**Figure 7:**
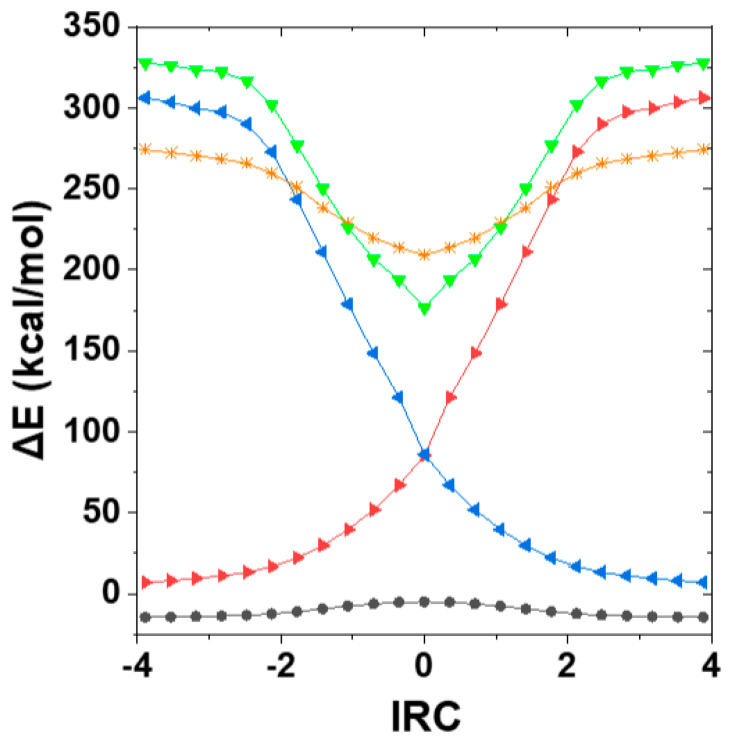
Potential curves along the IRC for F^−^ + CH_3_F → FCH_3_ + F^−^ degenerate exchange reaction, showing adiabatic ground-state B3LYP/6-311++G** (circles, black) and first excited state TD (*, gold) potential curves, compared with diabatic *E*_$DEL_(*n*_F_-σ_CF_*) potentials for product (◄, blue) or reactant (►, red) species and corresponding two-state prediction, Equation (19), of the excited-state potential (▼, green). All energy values are expressed with respect to isolated F^−^ + CH_3_F reactant species (*E* = −239.680066 a.u.).

Figure 7 shows the adiabatic ℇ_g.s._^(KS)^, ℇ_x.s._^(TD)^, diabatic $DEL-deletion H_11_, H_22_, and two-state ℇ_x.s.._^(2^^─st)^ energy curves along the computed IRC. In this case the mirror-image symmetry between KS ground-state and TD excited-state curves is more complex, with more pronounced attractive well in ℇ_x.s._^(TD)^ (*, gold) than repulsive barrier in ℇ_g.s._^(KS)^ (circles, black). The two-state model ℇ_x.s._^(2 − st)^ (▼, green) captures and further accentuates this asymmetry, dropping deeper than ℇ_x.s._^(TD)^ near IRC = 0 but rising above (and roughly parallel to) the TD excited-state curve as the IRC approaches its outer limits. Given the simplicity of its construction, the ℇ_x.s._^(2 − st)^ seems to form a reasonable first approximation to the landscape and excitation energy of ℇ_x.s._^(TD)^.

The TD excitation energy (Δ*E*^(TD)^) and corresponding two-state estimate (Δ*E*^(2^^-st^) depend on chosen DFT functional and basis level (here, B3LYP/6-311++G**), as well as the specific geometry. To exhibit the sensitivity to method choice, Table 2 displays corresponding Δ*E*^(2^^-st)^ and Δ*E*^(TD)^ (or Δ*E*^CIS)^ for HF) values for a variety of alternative HF/DFT methods (including CAM-B3LYP [55], M06 [56], wB97XD [57]) at transition-state geometry (IRC = 0). The percentage errors of Δ*E*^(2^^-st)^ from the full Δ*E*^(CIS/TD)^ are all in the range 19–31% (20% for B3LYP), indicating relatively modest performance differences among the various functionals.

**Figure 8:**
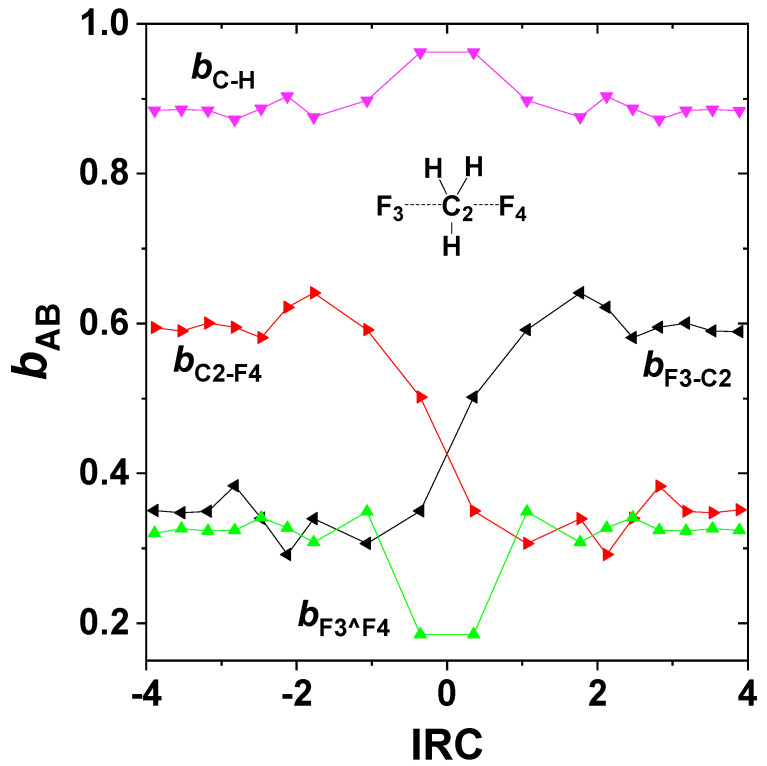
NRT bond orders along the IRC for the TD excited state of the F^−^ + CH_3_F exchange reaction, showing the inverted bond-order pattern for the excited-state potential compared to that of the ground-state (Figure 4). Note also the significantly increased F_3_^F_4_ long-bond order throughout the excited-state potential.

More detailed electronic relationships between the strongly coupled states can also be examined. Unfortunately, the numerical TD 1-particle density matrix is only an approximation to that of a properly antisymmetric *N*-particle wavefunction. Although the FIXDM keyword attempts to restore proper *N*-representable mathematical structure [64] to the numerical TD density, some issues remain that afflict the numerical stability of calculated excited-state bond orders. NRT analysis can be carried out at most points of the IRC to give a qualitative impression of TD excited-state bond orders as shown in Figure 8, for direct comparison with the ground-state bond orders of Figure 5. Although the TD results of Figure 8 show considerable numerical uncertainty, the evident complementarity of bond-order trends in the two states (i.e., with bond-order increases in one state being mirrored by decreases in the other at each IRC step) is qualitatively consistent with two-state bond conservation (22).

### 4.3. Polyene Bond Shifts of Cyanine Dyes 

The previous example deals with spectral shifts associated with reactive structural changes that are seldom directly measurable with conventional spectroscopic instrumentation. Typically, spectral investigations are reserved for equilibrium species having chromophoric properties in a desired wavelength region, such as the visible region of commercial dyestuffs. In such cases the spectral shifts of interest are those associated with the effects of chemical substitution or other systematic structural variations, which may again be estimated with the NBO-based two-state model.

As a more spectroscopically pertinent example of spectral bond-shifting phenomena, we consider the family of cationic diaminopolyenes (“cyanine” dyes, named for their cyan-colored hues [65]) that exhibit a common chromophoric bonding motif of the form

H_2_N(CH)_2n+1_NH_2_^+^, *n* = 2, 3,… (25)

In this case, bond-shifting is not due to chemical reaction but to light absorption itself, which induces concerted polyene bond shift and long-range charge transfer in the chromophoric pi system, viz., for *n* = 2,

(26) $$H_{2}N^{+}=\mathrm{CHCH}=\mathrm{CHCH}={\mathrm{CHNH}}_{2}\;\begin{array}{c} h\nu \\ \to \end{array}\;H_{2}\mathrm{NCH}=\mathrm{CHCH}=\mathrm{CHCH}=N^{+}H_{2}$$

**Figure 9:**
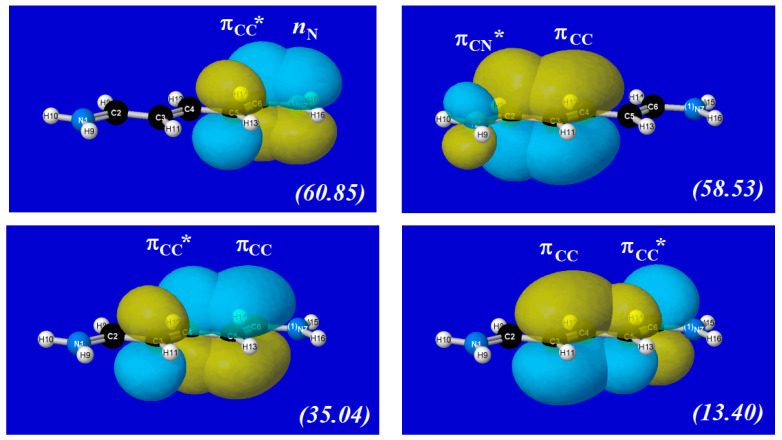
(Pre-)NBO overlap diagrams for leading donor-acceptor interactions in the l.h.s. bonding pattern of cyanine dye (26), with associated 2nd-order stabilization estimate |Δ*E*_DA_^(2)^| (kcal/mol) in parentheses.

Such concerted bond-shifts along a polyene chain necessarily involve a cooperative sequence of localized *n*_N_ → π_CC_*, π_CC_ → π_CC_*, and π_CC_ → π_CN_* interactions. Figure 9 illustrates the four leading NBO donor-acceptor interactions for the left-side Lewis-structural motif of (26), showing the dominance of terminal *n*_N_ → π_CC_* and π_CC_ → π_CN_* interactions over the interior π_CC_ → π_CC_* interactions of the polyene chain. Of the two π_CC_ → π_CC_* interactions depicted in the lower panels of Figure 9, the cooperatively ordered (“leftward-CT”) interaction (35.04 kcal/mol) is far more important than the oppositely directed interaction (13.40 kcal/mol) in achieving the concerted three-bond shift of (19). We primarily focus on such cooperatively ordered NBO interactions of the concerted polyene bond shift in results to follow.

Table 3 (a)–(c) displays results for the sequence of cyanine dyes, H_2_N(CH)_2n+1_NH_2_^+^, (*n* = 2–5), showing perturbative (Δ*E*_DA_^(2)^) vs. $DEL-deletion (Δ*E*_DA_^($DEL)^) estimates of stabilization energy (kcal/mol) for leading NBO interactions of the concerted bond shift: (a) *n*_N_ → π_CC_*, (b) π_CC_ → π_CN_*, (c) π_CC_ → π_CC_* (multiple). Because the concerted chromophoric bond shift in each cyanine-*n* chain involves *n* + 1 distinct contributing NBO donor-acceptor interactions (each necessary to achieve the desired symmetric 2-resonance form), we adopt the *average* of the associated $DEL-deletion values as a simple composite measure of interaction strength,

δ _n_^(av)^ = (*n* + 1)^−1^ ∑_i_ Δ*E*_DA(i)_^($DEL)^ (27)

with values as shown in the final row of Table 3.

The two-state model takes a particularly simple form in this case. The diabatic H_11(n)_ for each cyanine-*n* (relative to the ground-state energy ℇ_g.s.(n)_^(KS)^) satisfies

H_11(n)_ − δ_n_^(av)^ = ℇ_g.s.(n)_^(KS)^ (28)

In this degenerate limit (H_11_ = H_22_, Δ_1_ = Δ_2_) the corresponding two-state ℇ_x.s.(n)_^(2 − st)^ estimate is

ℇ_x.s.(n)_^(2 − st)^ = H_11(n)_ + δ_n_^(av)^ (29)

or equivalently,

ℇ_x.s.(n)_^(2 − st)^ = ℇ_g.s.(n)_^(KS)^ + 2 δ_n_^(av)^ (30)

One can see from Table 3 that the individual Δ*E*_DA_^($DEL)^ stabilizations for each interaction type (*n*_N_ → π_CC_*, π_CC_ → π_CC_*, or π_CC_ → π_CN_*) diminish monotonically with increasing polyene chain-length *n*. The same trend is necessarily true for δ_n_^(av)^ and the two-state estimate of excitation energy Δℇ_(n)_^(2 − st)^, viz.,

Δℇ_(n)_^(2 − st)^ = ℇ_x.s.(n)_^(2 − st)^ − ℇ_g.s.(n)_^(KS)^ = 2 δ_n_^(av)^ (31)

Thus, even without direct comparisons with excited-state calculations, one can see that the simple two-state model successfully captures the well-known empirical trend toward red-shifting of excitation energy as chain-length *n* increases [66,67]. Table 4 shows the direct numerical comparisons of the two-state estimate Δℇ_(n)_^(2 − st)^ with full TD calculations of spectral excitation energy for each considered member of the cyanine-*n* series. The comparisons demonstrate that the NBO-based Δℇ_(n)_^(2 − st)^ model is not only qualitatively consistent with the expected red-shifting trend, but tracks the actual *n*-dependent shifts of full TD theory in remarkably parallel fashion.

**Figure 10:**
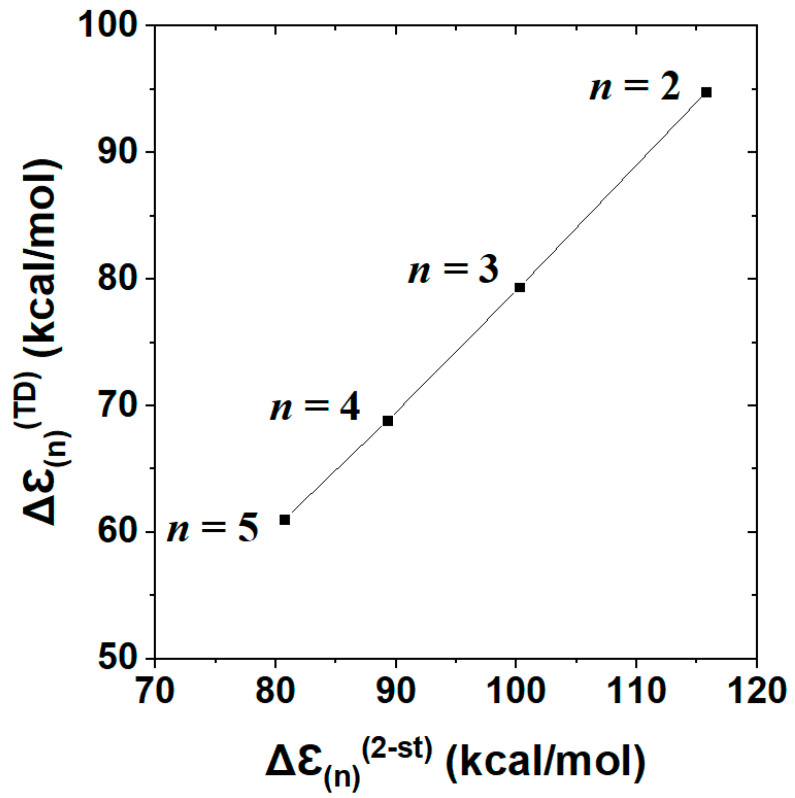
Correlation plot of excitation energies from NBO/NRT-based two-state model (Δℇ_(n)_^(2 − st)^) vs. full TD calculation (Δℇ_(n)_^(TD)^) for the sequence of cyanine-*n* dyes (*n* = 2–5), well represented by least-squares regression fit, Equation (32) (Pearson |χ|^2^ = 0.9996).

Figure 10 displays the near-perfect linear correlation between two-state Δℇ_(n)_^(2 − st)^ vs. full TD Δℇ_(n)_^(TD)^ descriptors (Table 4) of *n*-dependent cyanine chromophores. The displayed correlation, with Pearson correlation coefficient |χ|^2^ = 0.9996, corresponds to a least-squares regression fit

Δℇ_(n)_^(TD)^ = 0.9654*Δℇ_(n)_^(2 − st)^ − 17.15 (32)

with near-unit slope and near-constant (ca. 17 kcal/mol) offset of Δℇ_(n)_^(2 − st)^ above full Δℇ_(n)_^(TD)^ at each *n*. Both features of the observed two-state vs. TD correlations suggest the accuracy and robustness of the two-state description across a range of homologous species. Although the present results for equilibrium cyanine dyes do not probe geometrical aspects of excited-state landscape in the manner of a reactive process such as that for F^−^⋯CH_3_F, they successfully test the predicted two-state *magnitudes* of excitation energy over a range of chromophore chain lengths. The electronic logic of terminal vs. interior NBO donor-acceptor interactions (Figure 9) evidently gives predictive correlations (Figure 10) that go well beyond the elementary “free electron” (particle in a box) picture [66,67] of spectroscopic color shift with increasing polyene chain length. Comparisons of TD-DFT with higher levels of theory are described elsewhere [68].

## 5. Concluding Discussion

In the present work we have developed a simple NBO/NRT-based two-state model, based entirely on electronic ground-state descriptors, that is able to predict salient features of spectral excitation to the associated (resonance-coupled) excited state. We outlined the mathematical basis of this model and illustrated its application to two well-known prototype species: (i) F^−^⋯CH_3_F (as prototype for S_N_2-type chemical reactivity), and (ii) H_2_N(CH)_2n+1_NH_2_^+^, *n* = 2–5 (as prototype for spectral excitation in the polyene chromophore of cyanine dyes). In each case, the two-state model is found to yield useful estimates of the excited-state landscape (viz., well vs. barrier character or other details of resonance-shifted bond-order patterns) as well as magnitude of spectral excitation energy (viz., spectral shifts with respect to chromophore chain length). The deep connections of this model to familiar NBO/NRT descriptors of ground-state conjugative and hyperconjugative interactions also suggest how informed substitutional modifications of the ground-state bonding pattern can alter the spectral excitation energy or other excited-state landscape features in a desired manner. The current exploitation of simple connections between ground- and excited-state resonance bond-shifting also bears a close relationship to the recently developed NBO/NRT picture of pseudo Jahn-Teller effects [16].

The current study also has relevance for ongoing controversies concerning proper energy decomposition analysis (EDA) of electronic wavefunctions or densities [69]. Among the many suggested variants [70,71,72,73,74], the NBO-based natural energy decomposition analysis (NEDA) [75,76,77] is unique in its strict exclusion of *overlap* [78] from the conceptual building blocks (atomic orbitals, bond orbitals, or other “reference-fragment” orbitals) on which the analysis is based. Such overlap-free “natural” measures of orbital population [79] or “transfer” between orbitals thereby deviate significantly from the corresponding estimates inferred in alternative (overlap-dependent) EDA methods. These differences result in frequent sharp criticisms of NBO-based measures of CT interaction energy as, e.g., “excessively large, almost an order of magnitude larger than the other methods [and not] chemically credible” [80]. However, one can now recognize that estimates of CT-interaction energy differing by “order of magnitude” from NBO-based values must *fail* to capture the close connections to spectroscopic excitation values that are documented in this work. As shown in Equation (32) and Figure 10, a least-squares fit with slope of *near-unit* magnitude serves to give *near-perfect* correlation between the NBO-based two-state model and full TD calculation of the spectral shift in cyanine dyes. We conclude that the present results provide important spectroscopic evidence supporting NBO-based estimates of CT interactions in both ground- and excited-state analysis.

We mention finally that the elementary 2 × 2 secular determinant (7) on which the present two-state description is based can be extended to higher 3-, 4-,..., *k*-state description. The extension consists of generalizing Equation (18) for additional off-diagonal H_ij_ matrix elements and numerically solving the resulting D_k_(ℇ) secular equations for *k* > 2. However, such applications of higher multi-state NBO/NRT models of spectroscopic bond-shifting are beyond the scope of present work.

## Figures and Tables

**Table 1:** Symbolic association of resonance-structural bonding patterns (**I**, **II**) with “initial state” vs. “final state” of (i) chemical reaction, (ii) spectroscopic excitation, (iii) single-determinant wavefunction, or (iv) NBO electronic configuration.

*Resonance Structure*	I	II
(i) reactivity	reactant (***R***)	product (***P***)
(ii) spectroscopy	ground-state (***g.s.***)	excited state (***x.s.***)
(iii) single-determinant wavefunction	Ψ_**I**_^(SD)^	Ψ_**II**_^(SD)^
(iv) NBO configuration type	NLS	2*e* L→NL excitation

**Table 2:** Comparison of NBO-based two-state approximation (Δ*E*^(2 − st)^) vs. corresponding multi-configurational CIS or TD description (Δ*E*^(CIS/TD)^) (kcal/mol) for various alternative Hartree-Fock (HF) and DFT methods (B3LYP, CAM-B3LYP, M06, wB97XD; 6-311++G** basis level) at the transition state of S_N_2 reaction (24).

Method	HF	B3LYP	CAM-B3LYP	M06	wB97XD
Δ*E*^(2 − st)^	160	171	162	169	158
Δ*E*^(CIS/TD)^	213	214	228	208	230

**Table 3:** Leading NBO stabilizations (as estimated by perturbative Δ*E*_DA_^(2)^ and $DEL-deletion Δ*E*_DA_^($DEL)^ values) for concerted bond shifts in each cyanine-*n* polyene chain, with composite average δ_n_^(av)^ $DEL-deletion strength for each *n*.

**(a) *n*_N_** **→** π_CC_*	***n* = 2**	***n* = 3**	***n* = 4**	***n* = 5**
Δ*E*_DA_^(2)^	60.85	57.74	55.77	54.34
Δ*E*_DA_^($DEL)^	64.71	61.71	59.88	58.97
**(b) π_CC_** **→** π_CN_*				
Δ*E*_DA_^(2)^	58.53	60.42	61.73	62.88
Δ*E*_DA_^($DEL)^	67.14	66.30	64.99	64.14
**(c) π_CC_** **→** π_CC_*				
Δ*E*_DA_^(2)^	35.04	34.68 30.93	29.06 30.48 34.90	27.95 27.20 29.00 35.87
Δ*E*_DA_^($DEL)^	41.83	36.56 35.96	33.80 31.09 33.39	33.22 27.54 26.53 31.80
δ_n_^(av)^	57.89	50.13	44.63	40.37

**Table 4:** B3LYP/6-311++G** excitation energies Δℇ_(n)_ (kcal/mol) for each cyanine-*n* chromophore, as estimated by two-state model (upper) or full TD calculation (lower).

*n*	2	3	4	5
Δℇ_(n)_^(2 − st)^	115.78	100.26	89.26	80.74
Δℇ_(n)_^(TD)^	94.81	79.38	68.79	61.07

## Supplementary Materials

The Supplementary Materials are available online. Gaussian 16 input files including optimized geometry for all IRC and equilibrium species described in the text. Each input file also contains details of NBO keywords and keylists, as well as numerical values obtained in the corresponding output file.

## References

[1] Mulliken R.S. (1967). Spectroscopy, molecular orbitals, and chemical bonding (Nobel Prize lecture, 1966). Science.

[2] Lewis G.N., Kasha M. (1944). Phosphorescence and the triplet state. J. Am. Chem. Soc..

[3] Lewis G.N., Calvin M., Kasha M. (1949). Photomagnetism. Determination of the paramagnetic susceptibility of a dye in its phosphorescent state. J. Chem. Phys..

[4] Kasha M. (1984). The triplet state: An example of G. N. Lewis’ research style. J. Chem. Educ..

[5] Zimmerman H.E. (1982). Some theoretical aspects of organic photochemistry. Acc. Chem. Res..

[6] Zimmerman H.E., Alabugin I.V. (2000). Energy distribution and redistribution and chemical reactivity. Mechanistic and exploratory organic photochemistry. J. Am. Chem. Soc..

[7] Kutateladze A.G. (2005). Computational Methods in Photochemistry: Molecular and Supramolecular Photochemistry.

[8] Olivucci M. (2005). Computational Photochemistry.

[9] Albini A. (2016). Photochemistry: Past, Present and Future.

[10] Weinhold F., Landis C.R., Glendening E.D. (2016). What is NBO analysis and how is it useful?. Int. Rev. Phys. Chem..

[11] Weinhold F., Landis C.R. (2005). Valency and Bonding: A Natural Bond Orbital Donor-Acceptor Perspective.

[12] Rzepa H. The First Ever Curly Arrows. https://www.ch.imperial.ac.uk/rzepa/blog/?p=7234.

[13] Glendening E.D., Weinhold F. (1998). Natural resonance theory. I. General formulation. J. Comput. Chem..

[14] Glendening E.D., Wright S.J., Weinhold F. (2019). Efficient optimization of natural resonance theory weightings and bond orders by Gram-based convex programming. J. Comput. Chem..

[15] Glendening E.D., Landis C.R., Weinhold F. (2019). Resonance theory reboot. J. Am. Chem. Soc..

[16] Nori-Shargh D., Weinhold F. (2018). Natural bond orbital theory of pseudo Jahn-Teller effects. J. Phys. Chem. A.

[17] Bersuker I.B. (2013). Pseudo-Jahn-Teller effect—A two-state paradigm in formation, deformation, and transformation of molecular systems and solids. Chem. Rev..

[18] Ullrich C. (2012). Time-Dependent Density-Functional Theory: Concepts and Applications.

[19] Mulliken R.S., Person W.B. (1969). Molecular Complexes.

[20] Shaik S.S. (1981). What happens to molecules as they react? A valence bond approach to reactivity. J. Am. Chem. Soc..

[21] Shaik S.S., Pross A. (1982). S_N_2 reactivity of CH_3_X derivatives. A valence bond approach. J. Am. Chem. Soc..

[22] Shaik S.S. (1985). The collage of S_N_2 reactivity patterns. A state correlation diagram model. Prog. Phys. Org. Chem..

[23] Sini G., Shaik S., Hiberty P.C. (1992). Quantitative valence-bond computations of curve crossing diagrams for a gas-phase S_N_2 reactions, F**^−^** + CH_3_F → FCH_3_ + F**^−^**. J. Chem. Soc. Perkin Trans..

[24] Shaik S.S., Schlegel H.B., Wolfe S. (1992). Theoretical Aspects of Physical Organic Chemistry. Application to the S_N_2 Transition State.

[25] Song L., Wu W., Hiberty P.C., Shaik S. (2006). The identity S_N_2 reactions X**^−^** + CH_3_-X → X-CH_3_ + X**^−^** (X = F, Cl, Br and I) in vacuum and in aqueous solution: A valence bond study. Chem. Eur. J..

[26] Marcus R.A. (1956). On the theory of oxidation-reduction reactions involving electron transfer. J. Chem. Phys..

[27] Coulson C.A. (1964). The nature of the bonding in xenon fluorides and related molecules. J. Chem. Soc..

[28] Pimentel G.C. (1951). The bonding of trihalide and bifluoride ions by the molecular orbital method. J. Chem. Phys..

[29] Arunan E., Desiraju G.R., Klein R.A., Sadlej J., Scheiner S., Alkorta I., Clary D.C., Crabtree R.H., Dannenberg J.J., Hobza P. (2011). Definition of the hydrogen bond (IUPAC recommendations 2011). Pure Appl. Chem..

[30] Weinhold F., Klein R.A. (2014). What is a hydrogen bond? Resonance covalency in the supramolecular domain. Chem. Educ. Res. Pract..

[31] Jiao Y., Weinhold F. (2019). What is the nature of supramolecular bonding? Comprehensive NBO/NRT picture of halogen and pnicogen bonding in RPH_2_···IF/FI complexes (R=CH_3_, OH, CF_3_, CN, NO_2_). Molecules.

[32] Scheiner S. (2013). The pnicogen bond: Its relation to hydrogen, halogen, and other noncovalent bonds. Acc. Chem. Res..

[33] Del Bene J.E., Alkorta I., Elguero J., Scheiner S. (2015). Noncovalent Forces. Challenges and Advances in Computational Chemistry and Physics.

[34] Legon A.C. (1999). Prereactive complexes of dihalogens XY with Lewis bases B in the gas phase: A systematic case for the halogen analogue B∙∙∙XY of the hydrogen bond B∙∙∙HX. Angew. Chem. Int. Ed..

[35] Metrangolo P., Resnati G. (2001). Halogen bonding: A paradigm in supramolecular chemistry. Chem. Eur. J..

[36] Politzer P., Lane P., Concha M.C., Ma Y., Murray J.S. (2007). An overview of halogen bonding. J. Mol. Model.

[37] Bauza A., Frontera A. (2015). Aerogen bonding interaction: A new supramolecular force?. Angew. Chem. Int. Ed..

[38] Miao J., Xiong Z., Gao Y. (2018). The effects of aergogen bonding on the geometries and spectral properties of serveral small molecular clusters containing XeO_3_. J. Phys. Cond. Matter.

[39] Arunan E. (2013). Hydrogen bond seen, halogen bond defined and carbon bond proposed: Intermolecular bonding, a field that is maturing!. Curr. Sci..

[40] Michalczyk M., Zierkiewicz W., Wysokinski R., Scheiner S. (2019). Theoretical studies of IR and NMR spectral change induced by sigma-hole hydrogen, halogen, chalcogen, pnicogen, and tetrel bonds in a model protein environment. Molecules.

[41] Pauling L.C. (1977). The theory of resonance in chemistry. Proc. Roy. Soc..

[42] Frenking G., Krapp A. (2007). Unicorns in the world of chemical bonding models. J. Comput. Chem..

[43] Parr R.G., Yang W. (1994). Density-Functional Theory of Atoms and Molecules.

[44] Weinhold F., Landis C.R. (2012). Discovering Chemistry with Natural Bond Orbitals.

[45] Foresman J.B., Frisch A. (2015). Exploring Chemistry with Electronic Structure Methods.

[46] Glendening E.D., Landis C.R., Weinhold F. (2019). NBO 7.0: New vistas in localized and delocalized chemical bonding theory. J. Comput. Chem..

[47] Glendening E.D., Badenhoop J.K., Reed A.E., Carpenter J.E., Bohmann J.A., Morales C.M., Karafiloglou P., Landis C.R., Weinhold F. (2018). NBO 7.0.

[48] Weinhold F. (1999). Chemical bonding as a superposition phenomenon. J. Chem. Educ..

[49] Shahi A., Arunan E. (2014). Hydrogen bonding, halogen bonding and lithium bonding: An atoms in molecules and natural bond orbital perspective towards conservation of total bond order, inter- and intra-molecular bonding. Phys. Chem. Chem. Phys..

[50] Coulson C.A. (1939). The electronic structure of some polyenes and aromatic molecules. VII. Bonds of fractional order in the molecular orbital method. Proc. R. Soc. London A.

[51] Brown R.L. (1981). Rate constants for H-atom transfer reactions by the BEBO method. J. Res. Nat. Bur. Std..

[52] Badger R.M. (1934). A relation between internuclear distances and bond force constants. J. Chem. Phys..

[53] Becke A.D. (1993). A new mixing of Hartree-Fock and local density-functional theories. J. Chem. Phys..

[54] Casida M.E., Huix-Rotlant M. (2012). Progress in time-dependent density-functional theory. Ann. Rev. Phys. Chem..

[55] Takeshi Y., Tew D.P., Handy N.C. (2004). A new hybrid-exchange-correlation functional using the Coulomb-attenuating method (CAM-B3LYP). Chem. Phys. Lett..

[56] Zhao Y., Truhlar D.G. (2008). The M06 suite of density functionals for main group thermochemistry, thermochemical kinetics, noncovalent interactions, excited states, and transition elements: Two new functionals and systematic testing of four M06-class functionals and 12 other functionals. Theor. Chem. Acc..

[57] Chai J.-D., Head-Gordon M. (2008). Long-range corrected hybrid density functionals with damped atom-atom dispersion corrections. Phys. Chem. Chem. Phys..

[58] Frisch M.J., Trucks G.W., Schlegel H.B., Scuseria G.E., Robb M.A., Cheeseman J.R., Scalmani G., Barone V., Petersson G.A., Nakatsuji H. (2016). Gaussian 16, Revision C.01.

[59] Weinhold F., Glendening E.D. NBO 7.0 Manual. http://nbo7.chem.wisc.edu/nboman.pdf.

[60] Weinhold F., Phillips D., Glendening E.D., Foo Z.Y., Hanson R.M. (2018). NBOPro7@Jmol.

[61] Ingold C.K. (1934). Principles of an electronic theory of organic reactions. Chem. Rev..

[62] Fukui K. (1981). The path of chemical reactions—The IRC approach. Acc. Chem. Res..

[63] Landis C.R., Weinhold F. (2013). 3c/4e σ-type long-bonding: A novel NBO motif toward the metallic delocalization limit. Inorg. Chem..

[64] Mazziotti D.A. (2012). Structure of fermionic density matrices: Complete N-representability conditions. Phys. Rev. Lett..

[65] Shindy H.A. (2017). Fundamentals in the chemistry of cyanine dyes: A review. Dyes Pigm..

[66] Kuhn H.A. (1949). Quantum-mechanical theory of light absorption of organic dyes and similar compounds. J. Chem. Phys..

[67] Platt J.R. (1949). Classification of spectra of cata-condensed hydrocarbons. J. Chem. Phys..

[68] Le Guennic L., Jacquemin D. (2015). Taking up the cyanine challenge with quantum tools. Acc. Chem. Res..

[69] Andrés J., Ayres P.W., Boto R., Carbó-Dorca R., Ciolowski J., Chermette H., Contreras García J., Cooper D., Frenking G., Gatti C. (2019). Nine questions on energy decomposition analysis. J. Comput. Chem..

[70] Morokuma K. (1977). Why do molecules interact? The origin of electron donor-acceptor complexes, hydrogen bonding and proton affinity. Acc. Chem. Res..

[71] Bickelhaupt F.M., Baerends E.J., Lipkowitz K.B., Boyd D.B. (2000). Kohn-Sham density functional theory: Predicting and understanding chemistry. Reviews in Computational Chemistry.

[72] Mo Y., Gao J. (2000). Energy decomposition analysis of intermolecular interactions using a block-localized wave function approach. J. Chem. Phys..

[73] Khaliullin R.Z., Cobar E.A., Lochan R.C., Bell A.T., Head-Gordon M. (2007). Unravelling the origin of intermolecular interactions using absolutely localized molecular orbitals. J. Phys. Chem. A.

[74] Horn P.R., Mao Y., Head-Gordon M. (2016). Probing non-covalent interactions with a second generation energy decomposition analysis using localized molecular orbitals. Phys. Chem. Chem. Phys..

[75] Glendening E.D., Streitwieser A. (1994). Natural energy decomposition analysis: An energy partitioning procedure for molecular interactions with application to weak hydrogen bonding, strong ionic, and moderate donor-acceptor interactions. J. Chem. Phys..

[76] Schenter G.K., Glendening E.D. (1996). Natural energy decomposition analysis: The linear response electrical self energy. J. Phys. Chem..

[77] Glendening E.D. (2005). Natural energy decomposition analysis: Extension to density functional methods and analysis of cooperative effects in water clusters. J. Phys. Chem. A.

[78] Weinhold F., Carpenter J.E. (1988). Some remarks on nonorthogonal orbitals in quantum chemistry. J. Mol. Struct. THEOCHEM.

[79] Reed A.E., Weinstock R.B., Weinhold F. (1985). Natural population analysis. J. Chem. Phys..

[80] Phipps M.J.S., Fox T., Tautermann C.S., Skylaris C.-K. (2015). Energy decomposition analysis approaches and their evaluation on prototypical protein-drug interaction patterns. Chem. Soc. Rev..

