# Supplementary material for: NBO/NRT Two-State Theory of Bond-Shift Spectral Excitation
Source: Molecules. 2020 Sep 4;25(18):4052. doi: 10.3390/molecules25184052 (PMC7571041; doi:10.3390/molecules25184052)
Supplement: Supplementary file 1 [file molecules-25-04052-s001.pdf]

SUPPLEMENTARY INFORMATION:

NBO/NRT Two-State Theory of Bond-Shift Spectral Excitation

Yinchun Jiao[1] and Frank Weinhold[2]*

[1]*Key Laboratory of Theoretical Organic Chemistry and Functional Molecules, Ministry of Education, School of Chemistry and Chemical Engineering, Hunan University of Science and Technology, Xiangtan, 411201, China;* [2]*Theoretical Chemistry Institute and Department of Chemistry, University of Wisconsin-Madison, Madison, WI 53706, USA;* weinhold@chem.wisc.edu

Attached below are *Gaussian 16* input files with B3LYP/6-311++G** optimized geometries for:

(i) IRC for the S_N2 methyl fluoride exchange reaction (Sec. 4.2);

(ii) each of the cyanine dyes, $H_2N(CH)_{2n+1}NH_2^+$, *n* = 2-5 (Sec. 4.3).

Each file generally contains $NBO keylist input to generate the NBO archive (.47) file, PLOT files, and NRT bond-order output employed in the paper. The title section of each file may include leading vibrational frequencies (cm^{-1}) and full listing of vibrational and thermochemical values for stationary species as well as other information pertaining to calculated structural or spectroscopic properties (e.g., TD excited-state or $DEL-deletion data). For the $F^-{\cdots}CH_3F$ exchange reaction, the IRC input is provided both for NRT and TD output jobs, where the title lines in the latter include TD data for the "bright-state" root highlighted in Text Fig. 7. For the cyanine dyes, various forms of input files are provided for $DEL-deletion and TD excited-state jobs to illustrate the *Gaussian* and *NBO 7.0* keyword syntax employed for each job-type.

IRC for the S_N2 methyl fluoride exchange reaction

******************** NRT input *********************

```
%mem=2gb
%nprocshared=8
%chk=fch3f_m_irc
#b3lyp/6-311++g** pop=nbo7read

F...CH3F, #m11, IRC=-3.88656, E = -239.703163738

-1 1
                1          0.066352   -0.000000   -1.029368
                6          0.406087   -0.000000   -0.000074
                9          1.861432    0.000000   -0.000015
                9         -2.128121    0.000000    0.000077
                1          0.066277   -0.891378    0.514546
                1          0.066277    0.891378    0.514546
```

```
$nbo file=fch3f_m11 archive plot nrt nrtlong $end
 $NRTSTR
   STR        ! Wgt=83.51%; rhoNL=0.10054; D(0)=0.03416
     LONE 3 3 4 4 END
     BOND S 1 2 S 2 3 S 2 5 S 2 6 END
   END
   STR        ! Wgt=8.79%; rhoNL=0.54057; D(0)=0.07890
     LONE 3 4 4 3 END
     BOND S 1 2 S 2 4 S 2 5 S 2 6 END
   END
   STR        ! Wgt=2.89%; rhoNL=1.54547; D(0)=0.13332
     LONE 2 1 3 3 4 3 END
     BOND S 1 2 S 2 5 S 2 6 S 3 4 END
   END
   STR        ! Wgt=1.53%; rhoNL=0.91160; D(0)=0.10242
     LONE 2 1 3 2 4 4 END
     BOND S 1 3 S 2 3 S 2 5 S 2 6 END
   END
   STR        ! Wgt=1.53%; rhoNL=0.91159; D(0)=0.10242
     LONE 2 1 3 2 4 4 END
     BOND S 1 2 S 2 3 S 2 5 S 3 6 END
   END
   STR        ! Wgt=1.53%; rhoNL=0.91159; D(0)=0.10242
     LONE 2 1 3 2 4 4 END
     BOND S 1 2 S 2 3 S 2 6 S 3 5 END
   END
   STR        ! Wgt=1.53%; rhoNL=0.91160; D(0)=0.10242
     LONE 2 1 3 4 4 2 END
     BOND S 1 4 S 2 4 S 2 5 S 2 6 END
   END
   STR        ! Wgt=1.53%; rhoNL=0.91159; D(0)=0.10242
     LONE 2 1 3 4 4 2 END
     BOND S 1 2 S 2 4 S 2 5 S 4 6 END
   END
   STR        ! Wgt=1.53%; rhoNL=0.91159; D(0)=0.10242
     LONE 2 1 3 4 4 2 END
     BOND S 1 2 S 2 4 S 2 6 S 4 5 END
   END
 $END

--Link1--
%mem=2gb
%nprocshared=8
%chk=fch3f_m_irc
#b3lyp/6-311++g** pop=nbo7read

F...CH3F, #m10, IRC=-3.53240, E = -239.702977083

-1 1
                1          0.049433    0.000000   -1.030199
                6          0.384926    0.000000   -0.000022
                9          1.843073   -0.000000   -0.000008
                9         -2.093718   -0.000000    0.000027
                1          0.049418   -0.892157    0.515056
                1          0.049418    0.892157    0.515056

$nbo file=fch3f_m10 archive plot nrt nrtlong $end
 $NRTSTR
   STR        ! Wgt=83.51%; rhoNL=0.10054; D(0)=0.03416
     LONE 3 3 4 4 END
     BOND S 1 2 S 2 3 S 2 5 S 2 6 END
   END
   STR        ! Wgt=8.79%; rhoNL=0.54057; D(0)=0.07890
     LONE 3 4 4 3 END
     BOND S 1 2 S 2 4 S 2 5 S 2 6 END
   END
   STR        ! Wgt=2.89%; rhoNL=1.54547; D(0)=0.13332
     LONE 2 1 3 3 4 3 END
     BOND S 1 2 S 2 5 S 2 6 S 3 4 END
   END
```

```
   STR        ! Wgt=1.53%; rhoNL=0.91160; D(0)=0.10242
     LONE 2 1 3 2 4 4 END
     BOND S 1 3 S 2 3 S 2 5 S 2 6 END
   END
   STR        ! Wgt=1.53%; rhoNL=0.91159; D(0)=0.10242
     LONE 2 1 3 2 4 4 END
     BOND S 1 2 S 2 3 S 2 5 S 3 6 END
   END
   STR        ! Wgt=1.53%; rhoNL=0.91159; D(0)=0.10242
     LONE 2 1 3 2 4 4 END
     BOND S 1 2 S 2 3 S 2 6 S 3 5 END
   END
   STR        ! Wgt=1.53%; rhoNL=0.91160; D(0)=0.10242
     LONE 2 1 3 4 4 2 END
     BOND S 1 4 S 2 4 S 2 5 S 2 6 END
   END
   STR        ! Wgt=1.53%; rhoNL=0.91159; D(0)=0.10242
     LONE 2 1 3 4 4 2 END
     BOND S 1 2 S 2 4 S 2 5 S 4 6 END
   END
   STR        ! Wgt=1.53%; rhoNL=0.91159; D(0)=0.10242
     LONE 2 1 3 4 4 2 END
     BOND S 1 2 S 2 4 S 2 6 S 4 5 END
   END
 $END

--Link1--
%mem=2gb
%nprocshared=8
%chk=fch3f_m_irc
#b3lyp/6-311++g** pop=nbo7read

F...CH3F, #m9, IRC=-3.17829, E = -239.702585776

-1 1
                1                   0.032998   -0.000000   -1.031249
                6                   0.362713   -0.000000   -0.000003
                9                   1.825367    0.000000   -0.000006
                9                  -2.059356    0.000000    0.000008
                1                   0.033020   -0.893093    0.515621
                1                   0.033020    0.893093    0.515621

$nbo file=fch3f_m9 archive plot nrt nrtlong $end
 $NRTSTR
   STR        ! Wgt=83.51%; rhoNL=0.10054; D(0)=0.03416
     LONE 3 3 4 4 END
     BOND S 1 2 S 2 3 S 2 5 S 2 6 END
   END
   STR        ! Wgt=8.79%; rhoNL=0.54057; D(0)=0.07890
     LONE 3 4 4 3 END
     BOND S 1 2 S 2 4 S 2 5 S 2 6 END
   END
   STR        ! Wgt=2.89%; rhoNL=1.54547; D(0)=0.13332
     LONE 2 1 3 3 4 3 END
     BOND S 1 2 S 2 5 S 2 6 S 3 4 END
   END
   STR        ! Wgt=1.53%; rhoNL=0.91160; D(0)=0.10242
     LONE 2 1 3 2 4 4 END
     BOND S 1 3 S 2 3 S 2 5 S 2 6 END
   END
   STR        ! Wgt=1.53%; rhoNL=0.91159; D(0)=0.10242
     LONE 2 1 3 2 4 4 END
     BOND S 1 2 S 2 3 S 2 5 S 3 6 END
   END
   STR        ! Wgt=1.53%; rhoNL=0.91159; D(0)=0.10242
     LONE 2 1 3 2 4 4 END
     BOND S 1 2 S 2 3 S 2 6 S 3 5 END
   END
   STR        ! Wgt=1.53%; rhoNL=0.91160; D(0)=0.10242
     LONE 2 1 3 4 4 2 END
     BOND S 1 4 S 2 4 S 2 5 S 2 6 END
```

```
   END
   STR        ! Wgt=1.53%; rhoNL=0.91159; D(0)=0.10242
     LONE 2 1 3 4 4 2 END
     BOND S 1 2 S 2 4 S 2 5 S 4 6 END
   END
   STR        ! Wgt=1.53%; rhoNL=0.91159; D(0)=0.10242
     LONE 2 1 3 4 4 2 END
     BOND S 1 2 S 2 4 S 2 6 S 4 5 END
   END
 $END

--Link1--
%mem=2gb
%nprocshared=8
%chk=fch3f_m_irc
#b3lyp/6-311++g** pop=nbo7read

F...CH3F, #m8, IRC=-2.82421, E = -239.701949245

-1 1
                1          0.016735    0.000000   -1.032353
                6          0.342193   -0.000000    0.000002
                9          1.806183    0.000000   -0.000003
                9         -2.025094   -0.000000    0.000001
                1          0.016759   -0.894052    0.516181
                1          0.016759    0.894052    0.516181

$nbo file=fch3f_m8 archive plot nrt nrtlong $end
 $NRTSTR
   STR        ! Wgt=83.51%; rhoNL=0.10054; D(0)=0.03416
     LONE 3 3 4 4 END
     BOND S 1 2 S 2 3 S 2 5 S 2 6 END
   END
   STR        ! Wgt=8.79%; rhoNL=0.54057; D(0)=0.07890
     LONE 3 4 4 3 END
     BOND S 1 2 S 2 4 S 2 5 S 2 6 END
   END
   STR        ! Wgt=2.89%; rhoNL=1.54547; D(0)=0.13332
     LONE 2 1 3 3 4 3 END
     BOND S 1 2 S 2 5 S 2 6 S 3 4 END
   END
   STR        ! Wgt=1.53%; rhoNL=0.91160; D(0)=0.10242
     LONE 2 1 3 2 4 4 END
     BOND S 1 3 S 2 3 S 2 5 S 2 6 END
   END
   STR        ! Wgt=1.53%; rhoNL=0.91159; D(0)=0.10242
     LONE 2 1 3 2 4 4 END
     BOND S 1 2 S 2 3 S 2 5 S 3 6 END
   END
   STR        ! Wgt=1.53%; rhoNL=0.91159; D(0)=0.10242
     LONE 2 1 3 2 4 4 END
     BOND S 1 2 S 2 3 S 2 6 S 3 5 END
   END
   STR        ! Wgt=1.53%; rhoNL=0.91160; D(0)=0.10242
     LONE 2 1 3 4 4 2 END
     BOND S 1 4 S 2 4 S 2 5 S 2 6 END
   END
   STR        ! Wgt=1.53%; rhoNL=0.91159; D(0)=0.10242
     LONE 2 1 3 4 4 2 END
     BOND S 1 2 S 2 4 S 2 5 S 4 6 END
   END
   STR        ! Wgt=1.53%; rhoNL=0.91159; D(0)=0.10242
     LONE 2 1 3 4 4 2 END
     BOND S 1 2 S 2 4 S 2 6 S 4 5 END
   END
 $END

--Link1--
%mem=2gb
%nprocshared=8
%chk=fch3f_m_irc
```

```
#b3lyp/6-311++g** pop=nbo7read

F...CH3F, #m7, IRC=-2.47107, E = -239.701020539

-1 1
                1          0.003581   -0.000000   -1.035329
                6          0.316307   -0.000000    0.000001
                9          1.790746    0.000000   -0.000001
                9         -1.991152   -0.000000    0.000000
                1          0.003598   -0.896626    0.517667
                1          0.003598    0.896626    0.517667

$nbo file=fch3f_m7 archive plot nrt nrtlong $end
 $NRTSTR
   STR        ! Wgt=83.51%; rhoNL=0.10054; D(0)=0.03416
     LONE 3 3 4 4 END
     BOND S 1 2 S 2 3 S 2 5 S 2 6 END
   END
   STR        ! Wgt=8.79%; rhoNL=0.54057; D(0)=0.07890
     LONE 3 4 4 3 END
     BOND S 1 2 S 2 4 S 2 5 S 2 6 END
   END
   STR        ! Wgt=2.89%; rhoNL=1.54547; D(0)=0.13332
     LONE 2 1 3 3 4 3 END
     BOND S 1 2 S 2 5 S 2 6 S 3 4 END
   END
   STR        ! Wgt=1.53%; rhoNL=0.91160; D(0)=0.10242
     LONE 2 1 3 2 4 4 END
     BOND S 1 3 S 2 3 S 2 5 S 2 6 END
   END
   STR        ! Wgt=1.53%; rhoNL=0.91159; D(0)=0.10242
     LONE 2 1 3 2 4 4 END
     BOND S 1 2 S 2 3 S 2 5 S 3 6 END
   END
   STR        ! Wgt=1.53%; rhoNL=0.91159; D(0)=0.10242
     LONE 2 1 3 2 4 4 END
     BOND S 1 2 S 2 3 S 2 6 S 3 5 END
   END
   STR        ! Wgt=1.53%; rhoNL=0.91160; D(0)=0.10242
     LONE 2 1 3 4 4 2 END
     BOND S 1 4 S 2 4 S 2 5 S 2 6 END
   END
   STR        ! Wgt=1.53%; rhoNL=0.91159; D(0)=0.10242
     LONE 2 1 3 4 4 2 END
     BOND S 1 2 S 2 4 S 2 5 S 4 6 END
   END
   STR        ! Wgt=1.53%; rhoNL=0.91159; D(0)=0.10242
     LONE 2 1 3 4 4 2 END
     BOND S 1 2 S 2 4 S 2 6 S 4 5 END
   END
 $END

--Link1--
%mem=2gb
%nprocshared=8
%chk=fch3f_m_irc
#b3lyp/6-311++g** pop=nbo7read

F...CH3F, #m6, IRC=-2.12228, E = -239.699653885

-1 1
                1         -0.009065    0.000000   -1.040599
                6          0.280861   -0.000000   -0.000000
                9          1.784465    0.000000    0.000000
                9         -1.960427   -0.000000   -0.000000
                1         -0.009054   -0.901187    0.520299
                1         -0.009054    0.901187    0.520299

$nbo file=fch3f_m6 archive plot nrt nrtlong $end
 $NRTSTR
   STR        ! Wgt=83.51%; rhoNL=0.10054; D(0)=0.03416
```

```
      LONE 3 3 4 4 END
      BOND S 1 2 S 2 3 S 2 5 S 2 6 END
    END
    STR        ! Wgt=8.79%; rhoNL=0.54057; D(0)=0.07890
      LONE 3 4 4 3 END
      BOND S 1 2 S 2 4 S 2 5 S 2 6 END
    END
    STR        ! Wgt=2.89%; rhoNL=1.54547; D(0)=0.13332
      LONE 2 1 3 3 4 3 END
      BOND S 1 2 S 2 5 S 2 6 S 3 4 END
    END
    STR        ! Wgt=1.53%; rhoNL=0.91160; D(0)=0.10242
      LONE 2 1 3 2 4 4 END
      BOND S 1 3 S 2 3 S 2 5 S 2 6 END
    END
    STR        ! Wgt=1.53%; rhoNL=0.91159; D(0)=0.10242
      LONE 2 1 3 2 4 4 END
      BOND S 1 2 S 2 3 S 2 5 S 3 6 END
    END
    STR        ! Wgt=1.53%; rhoNL=0.91159; D(0)=0.10242
      LONE 2 1 3 2 4 4 END
      BOND S 1 2 S 2 3 S 2 6 S 3 5 END
    END
    STR        ! Wgt=1.53%; rhoNL=0.91160; D(0)=0.10242
      LONE 2 1 3 4 4 2 END
      BOND S 1 4 S 2 4 S 2 5 S 2 6 END
    END
    STR        ! Wgt=1.53%; rhoNL=0.91159; D(0)=0.10242
      LONE 2 1 3 4 4 2 END
      BOND S 1 2 S 2 4 S 2 5 S 4 6 END
    END
    STR        ! Wgt=1.53%; rhoNL=0.91159; D(0)=0.10242
      LONE 2 1 3 4 4 2 END
      BOND S 1 2 S 2 4 S 2 6 S 4 5 END
    END
  $END

--Link1--
%mem=2gb
%nprocshared=8
%chk=fch3f_m_irc
#b3lyp/6-311++g** pop=nbo7read

F...CH3F, #m5, IRC=-1.76986, E = -239.697548469

-1 1
               1         -0.015161    0.000000   -1.048552
               6          0.235928    0.000000   -0.000001
               9          1.791858    0.000000    0.000001
               9         -1.938455    0.000000    0.000000
               1         -0.015153   -0.908073    0.524274
               1         -0.015153    0.908073    0.524274

$nbo file=fch3f_m5 archive plot nrt nrtlong $end
 $NRTSTR
    STR        ! Wgt=83.51%; rhoNL=0.10054; D(0)=0.03416
      LONE 3 3 4 4 END
      BOND S 1 2 S 2 3 S 2 5 S 2 6 END
    END
    STR        ! Wgt=8.79%; rhoNL=0.54057; D(0)=0.07890
      LONE 3 4 4 3 END
      BOND S 1 2 S 2 4 S 2 5 S 2 6 END
    END
    STR        ! Wgt=2.89%; rhoNL=1.54547; D(0)=0.13332
      LONE 2 1 3 3 4 3 END
      BOND S 1 2 S 2 5 S 2 6 S 3 4 END
    END
    STR        ! Wgt=1.53%; rhoNL=0.91160; D(0)=0.10242
      LONE 2 1 3 2 4 4 END
      BOND S 1 3 S 2 3 S 2 5 S 2 6 END
    END
```

```
   STR        ! Wgt=1.53%; rhoNL=0.91159; D(0)=0.10242
     LONE 2 1 3 2 4 4 END
     BOND S 1 2 S 2 3 S 2 5 S 3 6 END
   END
   STR        ! Wgt=1.53%; rhoNL=0.91159; D(0)=0.10242
     LONE 2 1 3 2 4 4 END
     BOND S 1 2 S 2 3 S 2 6 S 3 5 END
   END
   STR        ! Wgt=1.53%; rhoNL=0.91160; D(0)=0.10242
     LONE 2 1 3 4 4 2 END
     BOND S 1 4 S 2 4 S 2 5 S 2 6 END
   END
   STR        ! Wgt=1.53%; rhoNL=0.91159; D(0)=0.10242
     LONE 2 1 3 4 4 2 END
     BOND S 1 2 S 2 4 S 2 5 S 4 6 END
   END
   STR        ! Wgt=1.53%; rhoNL=0.91159; D(0)=0.10242
     LONE 2 1 3 4 4 2 END
     BOND S 1 2 S 2 4 S 2 6 S 4 5 END
   END
 $END

--Link1--
%mem=2gb
%nprocshared=8
%chk=fch3f_m_irc
#b3lyp/6-311++g** pop=nbo7read

F...CH3F, #m4, IRC=-1.41605, E = E(RB3LYP) =  -239.694883585

-1 1
                1         -0.018107   -0.000000   -1.057221
                6          0.189320    0.000000   -0.000002
                9          1.804013   -0.000000    0.000001
                9         -1.920720   -0.000000    0.000001
                1         -0.018102   -0.915580    0.528607
                1         -0.018102    0.915580    0.528607

$nbo file=fch3f_m4 archive plot nrt nrtlong $end
 $NRTSTR
   STR        ! Wgt=83.51%; rhoNL=0.10054; D(0)=0.03416
     LONE 3 3 4 4 END
     BOND S 1 2 S 2 3 S 2 5 S 2 6 END
   END
   STR        ! Wgt=8.79%; rhoNL=0.54057; D(0)=0.07890
     LONE 3 4 4 3 END
     BOND S 1 2 S 2 4 S 2 5 S 2 6 END
   END
   STR        ! Wgt=2.89%; rhoNL=1.54547; D(0)=0.13332
     LONE 2 1 3 3 4 3 END
     BOND S 1 2 S 2 5 S 2 6 S 3 4 END
   END
   STR        ! Wgt=1.53%; rhoNL=0.91160; D(0)=0.10242
     LONE 2 1 3 2 4 4 END
     BOND S 1 3 S 2 3 S 2 5 S 2 6 END
   END
   STR        ! Wgt=1.53%; rhoNL=0.91159; D(0)=0.10242
     LONE 2 1 3 2 4 4 END
     BOND S 1 2 S 2 3 S 2 5 S 3 6 END
   END
   STR        ! Wgt=1.53%; rhoNL=0.91159; D(0)=0.10242
     LONE 2 1 3 2 4 4 END
     BOND S 1 2 S 2 3 S 2 6 S 3 5 END
   END
   STR        ! Wgt=1.53%; rhoNL=0.91160; D(0)=0.10242
     LONE 2 1 3 4 4 2 END
     BOND S 1 4 S 2 4 S 2 5 S 2 6 END
   END
   STR        ! Wgt=1.53%; rhoNL=0.91159; D(0)=0.10242
     LONE 2 1 3 4 4 2 END
     BOND S 1 2 S 2 4 S 2 5 S 4 6 END
```

```
   END
   STR        ! Wgt=1.53%; rhoNL=0.91159; D(0)=0.10242
     LONE 2 1 3 4 4 2 END
     BOND S 1 2 S 2 4 S 2 6 S 4 5 END
   END
 $END

--Link1--
%mem=2gb
%nprocshared=8
%chk=fch3f_m_irc
#b3lyp/6-311++g** pop=nbo7read

F...CH3F, #m3, IRC=-1.06204, E = -239.692192320

-1 1
                1         -0.016083    0.000000   -1.065313
                6          0.142177   -0.000000   -0.000002
                9          1.818172    0.000000    0.000001
                9         -1.905424    0.000000    0.000001
                1         -0.016078   -0.922586    0.532652
                1         -0.016078    0.922586    0.532652

$nbo file=fch3f_m3 archive plot nrt nrtlong $end
 $NRTSTR
   STR        ! Wgt=83.51%; rhoNL=0.10054; D(0)=0.03416
     LONE 3 3 4 4 END
     BOND S 1 2 S 2 3 S 2 5 S 2 6 END
   END
   STR        ! Wgt=8.79%; rhoNL=0.54057; D(0)=0.07890
     LONE 3 4 4 3 END
     BOND S 1 2 S 2 4 S 2 5 S 2 6 END
   END
   STR        ! Wgt=2.89%; rhoNL=1.54547; D(0)=0.13332
     LONE 2 1 3 3 4 3 END
     BOND S 1 2 S 2 5 S 2 6 S 3 4 END
   END
   STR        ! Wgt=1.53%; rhoNL=0.91160; D(0)=0.10242
     LONE 2 1 3 2 4 4 END
     BOND S 1 3 S 2 3 S 2 5 S 2 6 END
   END
   STR        ! Wgt=1.53%; rhoNL=0.91159; D(0)=0.10242
     LONE 2 1 3 2 4 4 END
     BOND S 1 2 S 2 3 S 2 5 S 3 6 END
   END
   STR        ! Wgt=1.53%; rhoNL=0.91159; D(0)=0.10242
     LONE 2 1 3 2 4 4 END
     BOND S 1 2 S 2 3 S 2 6 S 3 5 END
   END
   STR        ! Wgt=1.53%; rhoNL=0.91160; D(0)=0.10242
     LONE 2 1 3 4 4 2 END
     BOND S 1 4 S 2 4 S 2 5 S 2 6 END
   END
   STR        ! Wgt=1.53%; rhoNL=0.91159; D(0)=0.10242
     LONE 2 1 3 4 4 2 END
     BOND S 1 2 S 2 4 S 2 5 S 4 6 END
   END
   STR        ! Wgt=1.53%; rhoNL=0.91159; D(0)=0.10242
     LONE 2 1 3 4 4 2 END
     BOND S 1 2 S 2 4 S 2 6 S 4 5 END
   END
 $END

--Link1--
%mem=2gb
%nprocshared=8
%chk=fch3f_m_irc
#b3lyp/6-311++g** pop=nbo7read

F...CH3F, #m2, IRC=-0.70805, E = -239.689954267
```

```
-1 1
                1         -0.012332   -0.000000   -1.070594
                6          0.094872    0.000000   -0.000002
                9          1.832996   -0.000000    0.000001
                9         -1.890962    0.000000    0.000001
                1         -0.012327   -0.927159    0.535293
                1         -0.012327    0.927159    0.535293

$nbo file=fch3f_m2 archive plot nrt nrtlong $end
 $NRTSTR
   STR        ! Wgt=83.51%; rhoNL=0.10054; D(0)=0.03416
     LONE 3 3 4 4 END
     BOND S 1 2 S 2 3 S 2 5 S 2 6 END
   END
   STR        ! Wgt=8.79%; rhoNL=0.54057; D(0)=0.07890
     LONE 3 4 4 3 END
     BOND S 1 2 S 2 4 S 2 5 S 2 6 END
   END
   STR        ! Wgt=2.89%; rhoNL=1.54547; D(0)=0.13332
     LONE 2 1 3 3 4 3 END
     BOND S 1 2 S 2 5 S 2 6 S 3 4 END
   END
   STR        ! Wgt=1.53%; rhoNL=0.91160; D(0)=0.10242
     LONE 2 1 3 2 4 4 END
     BOND S 1 3 S 2 3 S 2 5 S 2 6 END
   END
   STR        ! Wgt=1.53%; rhoNL=0.91159; D(0)=0.10242
     LONE 2 1 3 2 4 4 END
     BOND S 1 2 S 2 3 S 2 5 S 3 6 END
   END
   STR        ! Wgt=1.53%; rhoNL=0.91159; D(0)=0.10242
     LONE 2 1 3 2 4 4 END
     BOND S 1 2 S 2 3 S 2 6 S 3 5 END
   END
   STR        ! Wgt=1.53%; rhoNL=0.91160; D(0)=0.10242
     LONE 2 1 3 4 4 2 END
     BOND S 1 4 S 2 4 S 2 5 S 2 6 END
   END
   STR        ! Wgt=1.53%; rhoNL=0.91159; D(0)=0.10242
     LONE 2 1 3 4 4 2 END
     BOND S 1 2 S 2 4 S 2 5 S 4 6 END
   END
   STR        ! Wgt=1.53%; rhoNL=0.91159; D(0)=0.10242
     LONE 2 1 3 4 4 2 END
     BOND S 1 2 S 2 4 S 2 6 S 4 5 END
   END
 $END

--Link1--
%mem=2gb
%nprocshared=8
%chk=fch3f_m_irc
#b3lyp/6-311++g** pop=nbo7read

F...CH3F, #m1, IRC=-0.35407, E = -239.688496623

-1 1
                1         -0.006549    0.000000   -1.072800
                6          0.047465    0.000000   -0.000000
                9          1.847731   -0.000000    0.000000
                9         -1.876669   -0.000000   -0.000000
                1         -0.006547   -0.929072    0.536400
                1         -0.006547    0.929072    0.536400

$nbo file=fch3f_m1 archive plot nrt nrtlong $end
 $NRTSTR
   STR        ! Wgt=83.51%; rhoNL=0.10054; D(0)=0.03416
     LONE 3 3 4 4 END
     BOND S 1 2 S 2 3 S 2 5 S 2 6 END
   END
   STR        ! Wgt=8.79%; rhoNL=0.54057; D(0)=0.07890
```

```
      LONE 3 4 4 3 END
      BOND S 1 2 S 2 4 S 2 5 S 2 6 END
    END
    STR        ! Wgt=2.89%; rhoNL=1.54547; D(0)=0.13332
      LONE 2 1 3 3 4 3 END
      BOND S 1 2 S 2 5 S 2 6 S 3 4 END
    END
    STR        ! Wgt=1.53%; rhoNL=0.91160; D(0)=0.10242
      LONE 2 1 3 2 4 4 END
      BOND S 1 3 S 2 3 S 2 5 S 2 6 END
    END
    STR        ! Wgt=1.53%; rhoNL=0.91159; D(0)=0.10242
      LONE 2 1 3 2 4 4 END
      BOND S 1 2 S 2 3 S 2 5 S 3 6 END
    END
    STR        ! Wgt=1.53%; rhoNL=0.91159; D(0)=0.10242
      LONE 2 1 3 2 4 4 END
      BOND S 1 2 S 2 3 S 2 6 S 3 5 END
    END
    STR        ! Wgt=1.53%; rhoNL=0.91160; D(0)=0.10242
      LONE 2 1 3 4 4 2 END
      BOND S 1 4 S 2 4 S 2 5 S 2 6 END
    END
    STR        ! Wgt=1.53%; rhoNL=0.91159; D(0)=0.10242
      LONE 2 1 3 4 4 2 END
      BOND S 1 2 S 2 4 S 2 5 S 4 6 END
    END
    STR        ! Wgt=1.53%; rhoNL=0.91159; D(0)=0.10242
      LONE 2 1 3 4 4 2 END
      BOND S 1 2 S 2 4 S 2 6 S 4 5 END
    END
  $END

--Link1--
%mem=2gb
%nprocshared=8
%chk=fch3f_m_irc
#b3lyp/6-311++g** pop=nbo7read

F...CH3F  SN2 t.s., IRC = 0.00, E(RB3LYP) =  -239.687990417
nu: -465,314(2),342,1043(2),1200,1389(2),3155,3346(2)
 Vibrational temperatures:    451.12   451.12   492.69  1501.26  1501.26
          (Kelvin)           1727.70  1997.85  1997.85  4539.86  4814.50
                             4814.50
 Zero-point correction=                           0.038460 (Hartree/Particle)
 Thermal correction to Energy=                    0.042564
 Thermal correction to Enthalpy=                  0.043508
 Thermal correction to Gibbs Free Energy=         0.014548
 Sum of electronic and zero-point Energies=           -239.649530
 Sum of electronic and thermal Energies=              -239.645426
 Sum of electronic and thermal Enthalpies=            -239.644482
 Sum of electronic and thermal Free Energies=         -239.673442

-1  1
H
C    1   r2
F    2   r3    1  90.
F    2   r3    1  90.   3   180.
H    2   r2    3  90.   1   120.
H    2   r2    3  90.   1  -120.

r2  1.0728
r3  1.8622

$nbo file=fch3f_m_ts archive archive plot nrt nrtlong $end
 $NRTSTR
    STR        ! Wgt=83.51%; rhoNL=0.10054; D(0)=0.03416
      LONE 3 3 4 4 END
      BOND S 1 2 S 2 3 S 2 5 S 2 6 END
    END
    STR        ! Wgt=8.79%; rhoNL=0.54057; D(0)=0.07890
```

```
    LONE 3 4 4 3 END
    BOND S 1 2 S 2 4 S 2 5 S 2 6 END
  END
  STR        ! Wgt=2.89%; rhoNL=1.54547; D(0)=0.13332
    LONE 2 1 3 3 4 3 END
    BOND S 1 2 S 2 5 S 2 6 S 3 4 END
  END
  STR        ! Wgt=1.53%; rhoNL=0.91160; D(0)=0.10242
    LONE 2 1 3 2 4 4 END
    BOND S 1 3 S 2 3 S 2 5 S 2 6 END
  END
  STR        ! Wgt=1.53%; rhoNL=0.91159; D(0)=0.10242
    LONE 2 1 3 2 4 4 END
    BOND S 1 2 S 2 3 S 2 5 S 3 6 END
  END
  STR        ! Wgt=1.53%; rhoNL=0.91159; D(0)=0.10242
    LONE 2 1 3 2 4 4 END
    BOND S 1 2 S 2 3 S 2 6 S 3 5 END
  END
  STR        ! Wgt=1.53%; rhoNL=0.91160; D(0)=0.10242
    LONE 2 1 3 4 4 2 END
    BOND S 1 4 S 2 4 S 2 5 S 2 6 END
  END
  STR        ! Wgt=1.53%; rhoNL=0.91159; D(0)=0.10242
    LONE 2 1 3 4 4 2 END
    BOND S 1 2 S 2 4 S 2 5 S 4 6 END
  END
  STR        ! Wgt=1.53%; rhoNL=0.91159; D(0)=0.10242
    LONE 2 1 3 4 4 2 END
    BOND S 1 2 S 2 4 S 2 6 S 4 5 END
  END
 $END

--Link1--
%mem=2gb
%nprocshared=8
%chk=fch3f_m_irc
#b3lyp/6-311++g** pop=nbo7read

F...CH3F, #p1, IRC=0.35408, E = E(RB3LYP) =  -239.688496623

-1 1
                1          0.006549   -0.000000   -1.072800
                6         -0.047465   -0.000000    0.000000
                9          1.876669    0.000000   -0.000000
                9         -1.847731    0.000000    0.000000
                1          0.006547   -0.929072    0.536400
                1          0.006547    0.929072    0.536400

$nbo file=fch3f_p1 archive plot nrt nrtlong $end
 $NRTSTR
  STR        ! Wgt=83.51%; rhoNL=0.10054; D(0)=0.03416
    LONE 3 3 4 4 END
    BOND S 1 2 S 2 3 S 2 5 S 2 6 END
  END
  STR        ! Wgt=8.79%; rhoNL=0.54057; D(0)=0.07890
    LONE 3 4 4 3 END
    BOND S 1 2 S 2 4 S 2 5 S 2 6 END
  END
  STR        ! Wgt=2.89%; rhoNL=1.54547; D(0)=0.13332
    LONE 2 1 3 3 4 3 END
    BOND S 1 2 S 2 5 S 2 6 S 3 4 END
  END
  STR        ! Wgt=1.53%; rhoNL=0.91160; D(0)=0.10242
    LONE 2 1 3 2 4 4 END
    BOND S 1 3 S 2 3 S 2 5 S 2 6 END
  END
  STR        ! Wgt=1.53%; rhoNL=0.91159; D(0)=0.10242
    LONE 2 1 3 2 4 4 END
    BOND S 1 2 S 2 3 S 2 5 S 3 6 END
  END
```

```
   STR        ! Wgt=1.53%; rhoNL=0.91159; D(0)=0.10242
     LONE 2 1 3 2 4 4 END
     BOND S 1 2 S 2 3 S 2 6 S 3 5 END
   END
   STR        ! Wgt=1.53%; rhoNL=0.91160; D(0)=0.10242
     LONE 2 1 3 4 4 2 END
     BOND S 1 4 S 2 4 S 2 5 S 2 6 END
   END
   STR        ! Wgt=1.53%; rhoNL=0.91159; D(0)=0.10242
     LONE 2 1 3 4 4 2 END
     BOND S 1 2 S 2 4 S 2 5 S 4 6 END
   END
   STR        ! Wgt=1.53%; rhoNL=0.91159; D(0)=0.10242
     LONE 2 1 3 4 4 2 END
     BOND S 1 2 S 2 4 S 2 6 S 4 5 END
   END
 $END

--Link1--
%mem=2gb
%nprocshared=8
%chk=fch3f_m_irc
#b3lyp/6-311++g** pop=nbo7read

F...CH3F, #p2, IRC=0.70806, E = E(RB3LYP) =  -239.689954308

-1 1
                1          0.012332   -0.000000   -1.070595
                6         -0.094873   -0.000000   -0.000003
                9          1.890960    0.000000    0.000001
                9         -1.832993    0.000000    0.000001
                1          0.012327   -0.927159    0.535292
                1          0.012327    0.927159    0.535292

$nbo file=fch3f_p2 archive plot nrt nrtlong $end
 $NRTSTR
   STR        ! Wgt=83.51%; rhoNL=0.10054; D(0)=0.03416
     LONE 3 3 4 4 END
     BOND S 1 2 S 2 3 S 2 5 S 2 6 END
   END
   STR        ! Wgt=8.79%; rhoNL=0.54057; D(0)=0.07890
     LONE 3 4 4 3 END
     BOND S 1 2 S 2 4 S 2 5 S 2 6 END
   END
   STR        ! Wgt=2.89%; rhoNL=1.54547; D(0)=0.13332
     LONE 2 1 3 3 4 3 END
     BOND S 1 2 S 2 5 S 2 6 S 3 4 END
   END
   STR        ! Wgt=1.53%; rhoNL=0.91160; D(0)=0.10242
     LONE 2 1 3 2 4 4 END
     BOND S 1 3 S 2 3 S 2 5 S 2 6 END
   END
   STR        ! Wgt=1.53%; rhoNL=0.91159; D(0)=0.10242
     LONE 2 1 3 2 4 4 END
     BOND S 1 2 S 2 3 S 2 5 S 3 6 END
   END
   STR        ! Wgt=1.53%; rhoNL=0.91159; D(0)=0.10242
     LONE 2 1 3 2 4 4 END
     BOND S 1 2 S 2 3 S 2 6 S 3 5 END
   END
   STR        ! Wgt=1.53%; rhoNL=0.91160; D(0)=0.10242
     LONE 2 1 3 4 4 2 END
     BOND S 1 4 S 2 4 S 2 5 S 2 6 END
   END
   STR        ! Wgt=1.53%; rhoNL=0.91159; D(0)=0.10242
     LONE 2 1 3 4 4 2 END
     BOND S 1 2 S 2 4 S 2 5 S 4 6 END
   END
   STR        ! Wgt=1.53%; rhoNL=0.91159; D(0)=0.10242
     LONE 2 1 3 4 4 2 END
     BOND S 1 2 S 2 4 S 2 6 S 4 5 END
```

```
   END
 $END

--Link1--
%mem=2gb
%nprocshared=8
%chk=fch3f_m_irc
#b3lyp/6-311++g** pop=nbo7read

F...CH3F, #p3, IRC=1.06205, E = -239.692192374

-1 1
                1          0.016083    0.000000   -1.065313
                6         -0.142178    0.000000   -0.000003
                9          1.905423   -0.000000    0.000001
                9         -1.818170   -0.000000    0.000001
                1          0.016078   -0.922585    0.532651
                1          0.016078    0.922585    0.532651

$nbo file=fch3f_p3 archive plot nrt nrtlong $end
 $NRTSTR
   STR        ! Wgt=83.51%; rhoNL=0.10054; D(0)=0.03416
     LONE 3 3 4 4 END
     BOND S 1 2 S 2 3 S 2 5 S 2 6 END
   END
   STR        ! Wgt=8.79%; rhoNL=0.54057; D(0)=0.07890
     LONE 3 4 4 3 END
     BOND S 1 2 S 2 4 S 2 5 S 2 6 END
   END
   STR        ! Wgt=2.89%; rhoNL=1.54547; D(0)=0.13332
     LONE 2 1 3 3 4 3 END
     BOND S 1 2 S 2 5 S 2 6 S 3 4 END
   END
   STR        ! Wgt=1.53%; rhoNL=0.91160; D(0)=0.10242
     LONE 2 1 3 2 4 4 END
     BOND S 1 3 S 2 3 S 2 5 S 2 6 END
   END
   STR        ! Wgt=1.53%; rhoNL=0.91159; D(0)=0.10242
     LONE 2 1 3 2 4 4 END
     BOND S 1 2 S 2 3 S 2 5 S 3 6 END
   END
   STR        ! Wgt=1.53%; rhoNL=0.91159; D(0)=0.10242
     LONE 2 1 3 2 4 4 END
     BOND S 1 2 S 2 3 S 2 6 S 3 5 END
   END
   STR        ! Wgt=1.53%; rhoNL=0.91160; D(0)=0.10242
     LONE 2 1 3 4 4 2 END
     BOND S 1 4 S 2 4 S 2 5 S 2 6 END
   END
   STR        ! Wgt=1.53%; rhoNL=0.91159; D(0)=0.10242
     LONE 2 1 3 4 4 2 END
     BOND S 1 2 S 2 4 S 2 5 S 4 6 END
   END
   STR        ! Wgt=1.53%; rhoNL=0.91159; D(0)=0.10242
     LONE 2 1 3 4 4 2 END
     BOND S 1 2 S 2 4 S 2 6 S 4 5 END
   END
 $END

--Link1--
%mem=2gb
%nprocshared=8
%chk=fch3f_m_irc
#b3lyp/6-311++g** pop=nbo7read

F...CH3F, #p4, IRC=1.41605, E = -239.694883640

-1 1
                1          0.018107    0.000000   -1.057221
                6         -0.189321   -0.000000   -0.000002
                9          1.920719    0.000000    0.000001
```

```
                9         -1.804012    0.000000    0.000001
                1          0.018102   -0.915580    0.528607
                1          0.018102    0.915580    0.528607

$nbo file=fch3f_p4 archive plot nrt nrtlong $end
 $NRTSTR
   STR        ! Wgt=83.51%; rhoNL=0.10054; D(0)=0.03416
     LONE 3 3 4 4 END
     BOND S 1 2 S 2 3 S 2 5 S 2 6 END
   END
   STR        ! Wgt=8.79%; rhoNL=0.54057; D(0)=0.07890
     LONE 3 4 4 3 END
     BOND S 1 2 S 2 4 S 2 5 S 2 6 END
   END
   STR        ! Wgt=2.89%; rhoNL=1.54547; D(0)=0.13332
     LONE 2 1 3 3 4 3 END
     BOND S 1 2 S 2 5 S 2 6 S 3 4 END
   END
   STR        ! Wgt=1.53%; rhoNL=0.91160; D(0)=0.10242
     LONE 2 1 3 2 4 4 END
     BOND S 1 3 S 2 3 S 2 5 S 2 6 END
   END
   STR        ! Wgt=1.53%; rhoNL=0.91159; D(0)=0.10242
     LONE 2 1 3 2 4 4 END
     BOND S 1 2 S 2 3 S 2 5 S 3 6 END
   END
   STR        ! Wgt=1.53%; rhoNL=0.91159; D(0)=0.10242
     LONE 2 1 3 2 4 4 END
     BOND S 1 2 S 2 3 S 2 6 S 3 5 END
   END
   STR        ! Wgt=1.53%; rhoNL=0.91160; D(0)=0.10242
     LONE 2 1 3 4 4 2 END
     BOND S 1 4 S 2 4 S 2 5 S 2 6 END
   END
   STR        ! Wgt=1.53%; rhoNL=0.91159; D(0)=0.10242
     LONE 2 1 3 4 4 2 END
     BOND S 1 2 S 2 4 S 2 5 S 4 6 END
   END
   STR        ! Wgt=1.53%; rhoNL=0.91159; D(0)=0.10242
     LONE 2 1 3 4 4 2 END
     BOND S 1 2 S 2 4 S 2 6 S 4 5 END
   END
 $END

--Link1--
%mem=2gb
%nprocshared=8
%chk=fch3f_m_irc
#b3lyp/6-311++g** pop=nbo7read

F...CH3F, #p5, IRC=1.76987, E = -239.697548509

-1 1
                1          0.015161    0.000000   -1.048552
                6         -0.235929    0.000000   -0.000002
                9          1.938454   -0.000000    0.000000
                9         -1.791857   -0.000000    0.000001
                1          0.015154   -0.908073    0.524273
                1          0.015154    0.908073    0.524273

$nbo file=fch3f_p5 archive plot nrt nrtlong $end
 $NRTSTR
   STR        ! Wgt=83.51%; rhoNL=0.10054; D(0)=0.03416
     LONE 3 3 4 4 END
     BOND S 1 2 S 2 3 S 2 5 S 2 6 END
   END
   STR        ! Wgt=8.79%; rhoNL=0.54057; D(0)=0.07890
     LONE 3 4 4 3 END
     BOND S 1 2 S 2 4 S 2 5 S 2 6 END
   END
   STR        ! Wgt=2.89%; rhoNL=1.54547; D(0)=0.13332
```

```
      LONE 2 1 3 3 4 3 END
      BOND S 1 2 S 2 5 S 2 6 S 3 4 END
    END
    STR        ! Wgt=1.53%; rhoNL=0.91160; D(0)=0.10242
      LONE 2 1 3 2 4 4 END
      BOND S 1 3 S 2 3 S 2 5 S 2 6 END
    END
    STR        ! Wgt=1.53%; rhoNL=0.91159; D(0)=0.10242
      LONE 2 1 3 2 4 4 END
      BOND S 1 2 S 2 3 S 2 5 S 3 6 END
    END
    STR        ! Wgt=1.53%; rhoNL=0.91159; D(0)=0.10242
      LONE 2 1 3 2 4 4 END
      BOND S 1 2 S 2 3 S 2 6 S 3 5 END
    END
    STR        ! Wgt=1.53%; rhoNL=0.91160; D(0)=0.10242
      LONE 2 1 3 4 4 2 END
      BOND S 1 4 S 2 4 S 2 5 S 2 6 END
    END
    STR        ! Wgt=1.53%; rhoNL=0.91159; D(0)=0.10242
      LONE 2 1 3 4 4 2 END
      BOND S 1 2 S 2 4 S 2 5 S 4 6 END
    END
    STR        ! Wgt=1.53%; rhoNL=0.91159; D(0)=0.10242
      LONE 2 1 3 4 4 2 END
      BOND S 1 2 S 2 4 S 2 6 S 4 5 END
    END
  $END

--Link1--
%mem=2gb
%nprocshared=8
%chk=fch3f_m_irc
#b3lyp/6-311++g** pop=nbo7read

F...CH3F, #p6, IRC=2.12229, E = -239.699653902

-1 1
                1          0.009064    0.000000   -1.040599
                6         -0.280861    0.000000   -0.000001
                9          1.960426   -0.000000   -0.000000
                9         -1.784464   -0.000000    0.000001
                1          0.009054   -0.901187    0.520299
                1          0.009054    0.901187    0.520299

$nbo file=fch3f_p6 archive plot nrt nrtlong $end
  $NRTSTR
    STR        ! Wgt=83.51%; rhoNL=0.10054; D(0)=0.03416
      LONE 3 3 4 4 END
      BOND S 1 2 S 2 3 S 2 5 S 2 6 END
    END
    STR        ! Wgt=8.79%; rhoNL=0.54057; D(0)=0.07890
      LONE 3 4 4 3 END
      BOND S 1 2 S 2 4 S 2 5 S 2 6 END
    END
    STR        ! Wgt=2.89%; rhoNL=1.54547; D(0)=0.13332
      LONE 2 1 3 3 4 3 END
      BOND S 1 2 S 2 5 S 2 6 S 3 4 END
    END
    STR        ! Wgt=1.53%; rhoNL=0.91160; D(0)=0.10242
      LONE 2 1 3 2 4 4 END
      BOND S 1 3 S 2 3 S 2 5 S 2 6 END
    END
    STR        ! Wgt=1.53%; rhoNL=0.91159; D(0)=0.10242
      LONE 2 1 3 2 4 4 END
      BOND S 1 2 S 2 3 S 2 5 S 3 6 END
    END
    STR        ! Wgt=1.53%; rhoNL=0.91159; D(0)=0.10242
      LONE 2 1 3 2 4 4 END
      BOND S 1 2 S 2 3 S 2 6 S 3 5 END
    END
```

```
   STR        ! Wgt=1.53%; rhoNL=0.91160; D(0)=0.10242
     LONE 2 1 3 4 4 2 END
     BOND S 1 4 S 2 4 S 2 5 S 2 6 END
   END
   STR        ! Wgt=1.53%; rhoNL=0.91159; D(0)=0.10242
     LONE 2 1 3 4 4 2 END
     BOND S 1 2 S 2 4 S 2 5 S 4 6 END
   END
   STR        ! Wgt=1.53%; rhoNL=0.91159; D(0)=0.10242
     LONE 2 1 3 4 4 2 END
     BOND S 1 2 S 2 4 S 2 6 S 4 5 END
   END
 $END

--Link1--
%mem=2gb
%nprocshared=8
%chk=fch3f_m_irc
#b3lyp/6-311++g** pop=nbo7read

F...CH3F, #p7, IRC=2.47108, E = -239.701020545

-1 1
                1         -0.003582   -0.000000   -1.035330
                6         -0.316308   -0.000000    0.000000
                9          1.991152    0.000000    0.000000
                9         -1.790746    0.000000   -0.000001
                1         -0.003598   -0.896626    0.517667
                1         -0.003598    0.896626    0.517667

$nbo file=fch3f_p7 archive plot nrt nrtlong $end
 $NRTSTR
   STR        ! Wgt=83.51%; rhoNL=0.10054; D(0)=0.03416
     LONE 3 3 4 4 END
     BOND S 1 2 S 2 3 S 2 5 S 2 6 END
   END
   STR        ! Wgt=8.79%; rhoNL=0.54057; D(0)=0.07890
     LONE 3 4 4 3 END
     BOND S 1 2 S 2 4 S 2 5 S 2 6 END
   END
   STR        ! Wgt=2.89%; rhoNL=1.54547; D(0)=0.13332
     LONE 2 1 3 3 4 3 END
     BOND S 1 2 S 2 5 S 2 6 S 3 4 END
   END
   STR        ! Wgt=1.53%; rhoNL=0.91160; D(0)=0.10242
     LONE 2 1 3 2 4 4 END
     BOND S 1 3 S 2 3 S 2 5 S 2 6 END
   END
   STR        ! Wgt=1.53%; rhoNL=0.91159; D(0)=0.10242
     LONE 2 1 3 2 4 4 END
     BOND S 1 2 S 2 3 S 2 5 S 3 6 END
   END
   STR        ! Wgt=1.53%; rhoNL=0.91159; D(0)=0.10242
     LONE 2 1 3 2 4 4 END
     BOND S 1 2 S 2 3 S 2 6 S 3 5 END
   END
   STR        ! Wgt=1.53%; rhoNL=0.91160; D(0)=0.10242
     LONE 2 1 3 4 4 2 END
     BOND S 1 4 S 2 4 S 2 5 S 2 6 END
   END
   STR        ! Wgt=1.53%; rhoNL=0.91159; D(0)=0.10242
     LONE 2 1 3 4 4 2 END
     BOND S 1 2 S 2 4 S 2 5 S 4 6 END
   END
   STR        ! Wgt=1.53%; rhoNL=0.91159; D(0)=0.10242
     LONE 2 1 3 4 4 2 END
     BOND S 1 2 S 2 4 S 2 6 S 4 5 END
   END
 $END

--Link1--
```

```
%mem=2gb
%nprocshared=8
%chk=fch3f_m_irc
#b3lyp/6-311++g** pop=nbo7read

F...CH3F, #p8, IRC=2.82422, E = E(RB3LYP) =  -239.701949250

-1 1
                1         -0.016736    0.000000   -1.032353
                6         -0.342193    0.000000    0.000001
                9          2.025094   -0.000000    0.000001
                9         -1.806184   -0.000000   -0.000003
                1         -0.016759   -0.894052    0.516181
                1         -0.016759    0.894052    0.516181

$nbo file=fch3f_p8 archive plot nrt nrtlong $end
 $NRTSTR
   STR        ! Wgt=83.51%; rhoNL=0.10054; D(0)=0.03416
     LONE 3 3 4 4 END
     BOND S 1 2 S 2 3 S 2 5 S 2 6 END
   END
   STR        ! Wgt=8.79%; rhoNL=0.54057; D(0)=0.07890
     LONE 3 4 4 3 END
     BOND S 1 2 S 2 4 S 2 5 S 2 6 END
   END
   STR        ! Wgt=2.89%; rhoNL=1.54547; D(0)=0.13332
     LONE 2 1 3 3 4 3 END
     BOND S 1 2 S 2 5 S 2 6 S 3 4 END
   END
   STR        ! Wgt=1.53%; rhoNL=0.91160; D(0)=0.10242
     LONE 2 1 3 2 4 4 END
     BOND S 1 3 S 2 3 S 2 5 S 2 6 END
   END
   STR        ! Wgt=1.53%; rhoNL=0.91159; D(0)=0.10242
     LONE 2 1 3 2 4 4 END
     BOND S 1 2 S 2 3 S 2 5 S 3 6 END
   END
   STR        ! Wgt=1.53%; rhoNL=0.91159; D(0)=0.10242
     LONE 2 1 3 2 4 4 END
     BOND S 1 2 S 2 3 S 2 6 S 3 5 END
   END
   STR        ! Wgt=1.53%; rhoNL=0.91160; D(0)=0.10242
     LONE 2 1 3 4 4 2 END
     BOND S 1 4 S 2 4 S 2 5 S 2 6 END
   END
   STR        ! Wgt=1.53%; rhoNL=0.91159; D(0)=0.10242
     LONE 2 1 3 4 4 2 END
     BOND S 1 2 S 2 4 S 2 5 S 4 6 END
   END
   STR        ! Wgt=1.53%; rhoNL=0.91159; D(0)=0.10242
     LONE 2 1 3 4 4 2 END
     BOND S 1 2 S 2 4 S 2 6 S 4 5 END
   END
 $END

--Link1--
%mem=2gb
%nprocshared=8
%chk=fch3f_m_irc
#b3lyp/6-311++g** pop=nbo7read

F...CH3F, #p9, IRC=3.17830, E = -239.702585779

-1 1
                1         -0.032998   -0.000000   -1.031249
                6         -0.362713   -0.000000   -0.000003
                9          2.059356    0.000000    0.000009
                9         -1.825367    0.000000   -0.000006
                1         -0.033019   -0.893093    0.515620
                1         -0.033019    0.893093    0.515620
```

```
$nbo file=fch3f_p9 archive plot nrt nrtlong $end
 $NRTSTR
   STR        ! Wgt=83.51%; rhoNL=0.10054; D(0)=0.03416
     LONE 3 3 4 4 END
     BOND S 1 2 S 2 3 S 2 5 S 2 6 END
   END
   STR        ! Wgt=8.79%; rhoNL=0.54057; D(0)=0.07890
     LONE 3 4 4 3 END
     BOND S 1 2 S 2 4 S 2 5 S 2 6 END
   END
   STR        ! Wgt=2.89%; rhoNL=1.54547; D(0)=0.13332
     LONE 2 1 3 3 4 3 END
     BOND S 1 2 S 2 5 S 2 6 S 3 4 END
   END
   STR        ! Wgt=1.53%; rhoNL=0.91160; D(0)=0.10242
     LONE 2 1 3 2 4 4 END
     BOND S 1 3 S 2 3 S 2 5 S 2 6 END
   END
   STR        ! Wgt=1.53%; rhoNL=0.91159; D(0)=0.10242
     LONE 2 1 3 2 4 4 END
     BOND S 1 2 S 2 3 S 2 5 S 3 6 END
   END
   STR        ! Wgt=1.53%; rhoNL=0.91159; D(0)=0.10242
     LONE 2 1 3 2 4 4 END
     BOND S 1 2 S 2 3 S 2 6 S 3 5 END
   END
   STR        ! Wgt=1.53%; rhoNL=0.91160; D(0)=0.10242
     LONE 2 1 3 4 4 2 END
     BOND S 1 4 S 2 4 S 2 5 S 2 6 END
   END
   STR        ! Wgt=1.53%; rhoNL=0.91159; D(0)=0.10242
     LONE 2 1 3 4 4 2 END
     BOND S 1 2 S 2 4 S 2 5 S 4 6 END
   END
   STR        ! Wgt=1.53%; rhoNL=0.91159; D(0)=0.10242
     LONE 2 1 3 4 4 2 END
     BOND S 1 2 S 2 4 S 2 6 S 4 5 END
   END
 $END

--Link1--
%mem=2gb
%nprocshared=8
%chk=fch3f_m_irc
#b3lyp/6-311++g** pop=nbo7read

F...CH3F, #p10, IRC=3.53241, E = -239.702977085

-1 1
                1         -0.049433   -0.000000   -1.030199
                6         -0.384926    0.000000   -0.000022
                9          2.093719    0.000000    0.000027
                9         -1.843073   -0.000000   -0.000008
                1         -0.049418   -0.892157    0.515056
                1         -0.049418    0.892157    0.515056

$nbo file=fch3f_p10 archive plot nrt nrtlong $end
 $NRTSTR
   STR        ! Wgt=83.51%; rhoNL=0.10054; D(0)=0.03416
     LONE 3 3 4 4 END
     BOND S 1 2 S 2 3 S 2 5 S 2 6 END
   END
   STR        ! Wgt=8.79%; rhoNL=0.54057; D(0)=0.07890
     LONE 3 4 4 3 END
     BOND S 1 2 S 2 4 S 2 5 S 2 6 END
   END
   STR        ! Wgt=2.89%; rhoNL=1.54547; D(0)=0.13332
     LONE 2 1 3 3 4 3 END
     BOND S 1 2 S 2 5 S 2 6 S 3 4 END
   END
   STR        ! Wgt=1.53%; rhoNL=0.91160; D(0)=0.10242
```

```
    LONE 2 1 3 2 4 4 END
    BOND S 1 3 S 2 3 S 2 5 S 2 6 END
  END
  STR        ! Wgt=1.53%; rhoNL=0.91159; D(0)=0.10242
    LONE 2 1 3 2 4 4 END
    BOND S 1 2 S 2 3 S 2 5 S 3 6 END
  END
  STR        ! Wgt=1.53%; rhoNL=0.91159; D(0)=0.10242
    LONE 2 1 3 2 4 4 END
    BOND S 1 2 S 2 3 S 2 6 S 3 5 END
  END
  STR        ! Wgt=1.53%; rhoNL=0.91160; D(0)=0.10242
    LONE 2 1 3 4 4 2 END
    BOND S 1 4 S 2 4 S 2 5 S 2 6 END
  END
  STR        ! Wgt=1.53%; rhoNL=0.91159; D(0)=0.10242
    LONE 2 1 3 4 4 2 END
    BOND S 1 2 S 2 4 S 2 5 S 4 6 END
  END
  STR        ! Wgt=1.53%; rhoNL=0.91159; D(0)=0.10242
    LONE 2 1 3 4 4 2 END
    BOND S 1 2 S 2 4 S 2 6 S 4 5 END
  END
 $END

--Link1--
%mem=2gb
%nprocshared=8
%chk=fch3f_m_irc
#b3lyp/6-311++g** pop=nbo7read

F...CH3F, #p11, IRC=3.88657, E = -239.703163738

-1 1
                1         -0.066352   -0.000000   -1.029369
                6         -0.406087   -0.000000   -0.000074
                9          2.128121    0.000000    0.000077
                9         -1.861432    0.000000   -0.000015
                1         -0.066277   -0.891378    0.514546
                1         -0.066277    0.891378    0.514546

$nbo file=fch3f_p11 archive plot nrt nrtlong $end
 $NRTSTR
  STR        ! Wgt=83.51%; rhoNL=0.10054; D(0)=0.03416
    LONE 3 3 4 4 END
    BOND S 1 2 S 2 3 S 2 5 S 2 6 END
  END
  STR        ! Wgt=8.79%; rhoNL=0.54057; D(0)=0.07890
    LONE 3 4 4 3 END
    BOND S 1 2 S 2 4 S 2 5 S 2 6 END
  END
  STR        ! Wgt=2.89%; rhoNL=1.54547; D(0)=0.13332
    LONE 2 1 3 3 4 3 END
    BOND S 1 2 S 2 5 S 2 6 S 3 4 END
  END
  STR        ! Wgt=1.53%; rhoNL=0.91160; D(0)=0.10242
    LONE 2 1 3 2 4 4 END
    BOND S 1 3 S 2 3 S 2 5 S 2 6 END
  END
  STR        ! Wgt=1.53%; rhoNL=0.91159; D(0)=0.10242
    LONE 2 1 3 2 4 4 END
    BOND S 1 2 S 2 3 S 2 5 S 3 6 END
  END
  STR        ! Wgt=1.53%; rhoNL=0.91159; D(0)=0.10242
    LONE 2 1 3 2 4 4 END
    BOND S 1 2 S 2 3 S 2 6 S 3 5 END
  END
  STR        ! Wgt=1.53%; rhoNL=0.91160; D(0)=0.10242
    LONE 2 1 3 4 4 2 END
    BOND S 1 4 S 2 4 S 2 5 S 2 6 END
  END
```

```
   STR        ! Wgt=1.53%; rhoNL=0.91159; D(0)=0.10242
     LONE 2 1 3 4 4 2 END
     BOND S 1 2 S 2 4 S 2 5 S 4 6 END
   END
   STR        ! Wgt=1.53%; rhoNL=0.91159; D(0)=0.10242
     LONE 2 1 3 4 4 2 END
     BOND S 1 2 S 2 4 S 2 6 S 4 5 END
   END
 $END
```

******************** TD input *********************

```
%mem=2gb
%nprocshared=8
%chk=fch3f_m_td
#b3lyp/6-311++g** pop=nbo7read density=current td=(root=78,nstates=100)

F...CH3F, #m11, IRC=-3.88656, E = -239.703163738 (-14.49)
 Excited State  78:      Singlet-A'    12.5111 eV   99.10 nm  f=0.1587  <S**2>=$
       7 -> 18         0.69058
 This state for optimization and/or second-order correction.
 Total Energy, E(TD-HF/TD-DFT) =  -239.243389548 (274.02)

-1 1
                1          0.066352   -0.000000   -1.029368
                6          0.406087   -0.000000   -0.000074
                9          1.861432    0.000000   -0.000015
                9         -2.128121    0.000000    0.000077
                1          0.066277   -0.891378    0.514546
                1          0.066277    0.891378    0.514546

$nbo file=fch3f_td_m11 archive plot nrt nrtlong fixdm $end
 $NRTSTR
   STR        ! Wgt=88.03%; rhoNL=0.10054; D(0)=0.03416
     LONE 3 3 4 4 END
     BOND S 1 2 S 2 3 S 2 5 S 2 6 END
   END
   STR        ! Wgt=8.78%; rhoNL=0.54057; D(0)=0.07890
     LONE 3 4 4 3 END
     BOND S 1 2 S 2 4 S 2 5 S 2 6 END
   END
   STR        ! Wgt=2.93%; rhoNL=1.54547; D(0)=0.13332
     LONE 2 1 3 3 4 3 END
     BOND S 1 2 S 2 5 S 2 6 S 3 4 END
   END
   STR        ! Wgt=53.92%; rhoNL=1.31552; D(0)=0.09401
     LONE 2 1 3 3 4 3 END
     BOND S 1 2 S 2 5 S 2 6 S 3 4 END
   END
   STR        ! Wgt=7.23%; rhoNL=2.93826; D(0)=0.16580
     LONE 2 1 3 3 4 3 6 1 END
     BOND S 1 2 S 2 4 S 2 5 END
   END
   STR        ! Wgt=7.23%; rhoNL=2.93826; D(0)=0.16580
     LONE 2 1 3 3 4 3 6 1 END
     BOND S 1 2 S 2 3 S 2 5 END
   END
   STR        ! Wgt=7.23%; rhoNL=2.93826; D(0)=0.16580
     LONE 2 1 3 3 4 3 5 1 END
     BOND S 1 2 S 2 3 S 2 6 END
   END
   STR        ! Wgt=7.23%; rhoNL=2.93826; D(0)=0.16580
     LONE 2 1 3 3 4 3 5 1 END
     BOND S 1 2 S 2 4 S 2 6 END
   END
   STR        ! Wgt=7.23%; rhoNL=2.93826; D(0)=0.16580
     LONE 1 1 2 1 3 3 4 3 END
     BOND S 2 4 S 2 5 S 2 6 END
   END
   STR        ! Wgt=7.23%; rhoNL=2.93826; D(0)=0.16580
     LONE 1 1 2 1 3 3 4 3 END
```

```
    BOND S 2 3 S 2 5 S 2 6 END
  END
 $END

--Link1--
%mem=2gb
%nprocshared=8
%chk=fch3f_m_td
#b3lyp/6-311++g** pop=nbo7read density=current td=(root=73,nstates=100)

F...CH3F, #m10, IRC=-3.53240, E = -239.702977083 (-14.38)
 Excited State  73:      Singlet-A'    12.4289 eV   99.75 nm  f=0.1475  <S**2>=$
       7 -> 18         0.68829
 This state for optimization and/or second-order correction.
 Total Energy, E(TD-HF/TD-DFT) =  -239.246223106 (272.24)

-1 1
                1          0.049433    0.000000   -1.030199
                6          0.384926    0.000000   -0.000022
                9          1.843073   -0.000000   -0.000008
                9         -2.093718   -0.000000    0.000027
                1          0.049418   -0.892157    0.515056
                1          0.049418    0.892157    0.515056

$nbo file=fch3f_td_m10 archive plot nrt nrtlong $end
 $NRTSTR
   STR        ! Wgt=88.03%; rhoNL=0.10054; D(0)=0.03416
     LONE 3 3 4 4 END
     BOND S 1 2 S 2 3 S 2 5 S 2 6 END
   END
   STR        ! Wgt=8.78%; rhoNL=0.54057; D(0)=0.07890
     LONE 3 4 4 3 END
     BOND S 1 2 S 2 4 S 2 5 S 2 6 END
   END
   STR        ! Wgt=2.93%; rhoNL=1.54547; D(0)=0.13332
     LONE 2 1 3 3 4 3 END
     BOND S 1 2 S 2 5 S 2 6 S 3 4 END
   END
   STR        ! Wgt=53.92%; rhoNL=1.31552; D(0)=0.09401
     LONE 2 1 3 3 4 3 END
     BOND S 1 2 S 2 5 S 2 6 S 3 4 END
   END
   STR        ! Wgt=7.23%; rhoNL=2.93826; D(0)=0.16580
     LONE 2 1 3 3 4 3 6 1 END
     BOND S 1 2 S 2 4 S 2 5 END
   END
   STR        ! Wgt=7.23%; rhoNL=2.93826; D(0)=0.16580
     LONE 2 1 3 3 4 3 6 1 END
     BOND S 1 2 S 2 3 S 2 5 END
   END
   STR        ! Wgt=7.23%; rhoNL=2.93826; D(0)=0.16580
     LONE 2 1 3 3 4 3 5 1 END
     BOND S 1 2 S 2 3 S 2 6 END
   END
   STR        ! Wgt=7.23%; rhoNL=2.93826; D(0)=0.16580
     LONE 2 1 3 3 4 3 5 1 END
     BOND S 1 2 S 2 4 S 2 6 END
   END
   STR        ! Wgt=7.23%; rhoNL=2.93826; D(0)=0.16580
     LONE 1 1 2 1 3 3 4 3 END
     BOND S 2 4 S 2 5 S 2 6 END
   END
   STR        ! Wgt=7.23%; rhoNL=2.93826; D(0)=0.16580
     LONE 1 1 2 1 3 3 4 3 END
     BOND S 2 3 S 2 5 S 2 6 END
   END
 $END

--Link1--
%mem=2gb
%nprocshared=8
```

```
%chk=fch3f_m_td
#b3lyp/6-311++g** pop=nbo7read density=current td=(root=73,nstates=100)

F...CH3F, #m9, IRC=-3.17829, E = -239.702585776 (-14.13)
 Excited State  73:      Singlet-A'    12.3336 eV  100.53 nm  f=0.1166  <S**2>$
       7 -> 18         0.66811
      12 -> 29         0.15409
 This state for optimization and/or second-order correction.
 Total Energy, E(TD-HF/TD-DFT) =  -239.249333852 (270.29)

-1 1
                1                   0.032998   -0.000000   -1.031249
                6                   0.362713   -0.000000   -0.000003
                9                   1.825367    0.000000   -0.000006
                9                  -2.059356    0.000000    0.000008
                1                   0.033020   -0.893093    0.515621
                1                   0.033020    0.893093    0.515621

$nbo file=fch3f_td_m9 archive plot nrt nrtlong $end
 $NRTSTR
   STR        ! Wgt=88.03%; rhoNL=0.10054; D(0)=0.03416
     LONE 3 3 4 4 END
     BOND S 1 2 S 2 3 S 2 5 S 2 6 END
   END
   STR        ! Wgt=8.78%; rhoNL=0.54057; D(0)=0.07890
     LONE 3 4 4 3 END
     BOND S 1 2 S 2 4 S 2 5 S 2 6 END
   END
   STR        ! Wgt=2.93%; rhoNL=1.54547; D(0)=0.13332
     LONE 2 1 3 3 4 3 END
     BOND S 1 2 S 2 5 S 2 6 S 3 4 END
   END
   STR        ! Wgt=53.92%; rhoNL=1.31552; D(0)=0.09401
     LONE 2 1 3 3 4 3 END
     BOND S 1 2 S 2 5 S 2 6 S 3 4 END
   END
   STR        ! Wgt=7.23%; rhoNL=2.93826; D(0)=0.16580
     LONE 2 1 3 3 4 3 6 1 END
     BOND S 1 2 S 2 4 S 2 5 END
   END
   STR        ! Wgt=7.23%; rhoNL=2.93826; D(0)=0.16580
     LONE 2 1 3 3 4 3 6 1 END
     BOND S 1 2 S 2 3 S 2 5 END
   END
   STR        ! Wgt=7.23%; rhoNL=2.93826; D(0)=0.16580
     LONE 2 1 3 3 4 3 5 1 END
     BOND S 1 2 S 2 3 S 2 6 END
   END
   STR        ! Wgt=7.23%; rhoNL=2.93826; D(0)=0.16580
     LONE 2 1 3 3 4 3 5 1 END
     BOND S 1 2 S 2 4 S 2 6 END
   END
   STR        ! Wgt=7.23%; rhoNL=2.93826; D(0)=0.16580
     LONE 1 1 2 1 3 3 4 3 END
     BOND S 2 4 S 2 5 S 2 6 END
   END
   STR        ! Wgt=7.23%; rhoNL=2.93826; D(0)=0.16580
     LONE 1 1 2 1 3 3 4 3 END
     BOND S 2 3 S 2 5 S 2 6 END
   END
 $END

--Link1--
%mem=2gb
%nprocshared=8
%chk=fch3f_m_td
#b3lyp/6-311++g** pop=nbo7read density=current td=(root=72,nstates=100)

F...CH3F, #m8, IRC=-2.82421, E = -239.701949245 (-13.73)
 Excited State  72:      Singlet-A'    12.2343 eV  101.34 nm  f=0.1765  <S**2>$
       7 -> 18         0.62920
```

```
      12 -> 29        -0.27415
 This state for optimization and/or second-order correction.
 Total Energy, E(TD-HF/TD-DFT) =  -239.252346940 (268.40)

-1 1
                1          0.016735    0.000000   -1.032353
                6          0.342193   -0.000000    0.000002
                9          1.806183    0.000000   -0.000003
                9         -2.025094   -0.000000    0.000001
                1          0.016759   -0.894052    0.516181
                1          0.016759    0.894052    0.516181

$nbo file=fch3f_td_m8 archive plot nrt nrtlong $end
 $NRTSTR
   STR        ! Wgt=88.03%; rhoNL=0.10054; D(0)=0.03416
     LONE 3 3 4 4 END
     BOND S 1 2 S 2 3 S 2 5 S 2 6 END
   END
   STR        ! Wgt=8.78%; rhoNL=0.54057; D(0)=0.07890
     LONE 3 4 4 3 END
     BOND S 1 2 S 2 4 S 2 5 S 2 6 END
   END
   STR        ! Wgt=2.93%; rhoNL=1.54547; D(0)=0.13332
     LONE 2 1 3 3 4 3 END
     BOND S 1 2 S 2 5 S 2 6 S 3 4 END
   END
   STR        ! Wgt=53.92%; rhoNL=1.31552; D(0)=0.09401
     LONE 2 1 3 3 4 3 END
     BOND S 1 2 S 2 5 S 2 6 S 3 4 END
   END
   STR        ! Wgt=7.23%; rhoNL=2.93826; D(0)=0.16580
     LONE 2 1 3 3 4 3 6 1 END
     BOND S 1 2 S 2 4 S 2 5 END
   END
   STR        ! Wgt=7.23%; rhoNL=2.93826; D(0)=0.16580
     LONE 2 1 3 3 4 3 6 1 END
     BOND S 1 2 S 2 3 S 2 5 END
   END
   STR        ! Wgt=7.23%; rhoNL=2.93826; D(0)=0.16580
     LONE 2 1 3 3 4 3 5 1 END
     BOND S 1 2 S 2 3 S 2 6 END
   END
   STR        ! Wgt=7.23%; rhoNL=2.93826; D(0)=0.16580
     LONE 2 1 3 3 4 3 5 1 END
     BOND S 1 2 S 2 4 S 2 6 END
   END
   STR        ! Wgt=7.23%; rhoNL=2.93826; D(0)=0.16580
     LONE 1 1 2 1 3 3 4 3 END
     BOND S 2 4 S 2 5 S 2 6 END
   END
   STR        ! Wgt=7.23%; rhoNL=2.93826; D(0)=0.16580
     LONE 1 1 2 1 3 3 4 3 END
     BOND S 2 3 S 2 5 S 2 6 END
   END
 $END

--Link1--
%mem=2gb
%nprocshared=8
%chk=fch3f_m_td
#b3lyp/6-311++g** pop=nbo7read density=current td=(root=70,nstates=100)

F...CH3F, #m7, IRC=-2.47107, E = -239.701020539 (-13.15)
 Excited State  70:      Singlet-A'    12.0864 eV  102.58 nm  f=0.1582  <S**2>$
       9 -> 18         0.68396
 This state for optimization and/or second-order correction.
 Total Energy, E(TD-HF/TD-DFT) =  -239.256851767 (265.57)

-1 1
                1          0.003581   -0.000000   -1.035329
                6          0.316307   -0.000000    0.000001
```

```
                9          1.790746    0.000000   -0.000001
                9         -1.991152   -0.000000    0.000000
                1          0.003598   -0.896626    0.517667
                1          0.003598    0.896626    0.517667

$nbo file=fch3f_td_m7 archive plot nrt nrtlong $end
 $NRTSTR
   STR        ! Wgt=88.03%; rhoNL=0.10054; D(0)=0.03416
     LONE 3 3 4 4 END
     BOND S 1 2 S 2 3 S 2 5 S 2 6 END
   END
   STR        ! Wgt=8.78%; rhoNL=0.54057; D(0)=0.07890
     LONE 3 4 4 3 END
     BOND S 1 2 S 2 4 S 2 5 S 2 6 END
   END
   STR        ! Wgt=2.93%; rhoNL=1.54547; D(0)=0.13332
     LONE 2 1 3 3 4 3 END
     BOND S 1 2 S 2 5 S 2 6 S 3 4 END
   END
   STR        ! Wgt=53.92%; rhoNL=1.31552; D(0)=0.09401
     LONE 2 1 3 3 4 3 END
     BOND S 1 2 S 2 5 S 2 6 S 3 4 END
   END
   STR        ! Wgt=7.23%; rhoNL=2.93826; D(0)=0.16580
     LONE 2 1 3 3 4 3 6 1 END
     BOND S 1 2 S 2 4 S 2 5 END
   END
   STR        ! Wgt=7.23%; rhoNL=2.93826; D(0)=0.16580
     LONE 2 1 3 3 4 3 6 1 END
     BOND S 1 2 S 2 3 S 2 5 END
   END
   STR        ! Wgt=7.23%; rhoNL=2.93826; D(0)=0.16580
     LONE 2 1 3 3 4 3 5 1 END
     BOND S 1 2 S 2 3 S 2 6 END
   END
   STR        ! Wgt=7.23%; rhoNL=2.93826; D(0)=0.16580
     LONE 2 1 3 3 4 3 5 1 END
     BOND S 1 2 S 2 4 S 2 6 END
   END
   STR        ! Wgt=7.23%; rhoNL=2.93826; D(0)=0.16580
     LONE 1 1 2 1 3 3 4 3 END
     BOND S 2 4 S 2 5 S 2 6 END
   END
   STR        ! Wgt=7.23%; rhoNL=2.93826; D(0)=0.16580
     LONE 1 1 2 1 3 3 4 3 END
     BOND S 2 3 S 2 5 S 2 6 END
   END
 $END

--Link1--
%mem=2gb
%nprocshared=8
%chk=fch3f_m_td
#b3lyp/6-311++g** pop=nbo7read density=current td=(root=67,nstates=100)

F...CH3F, #m6, IRC=-2.12228, E = -239.699653885 (-12.29)
 Excited State  67:      Singlet-A'    11.7847 eV  105.21 nm  f=0.1106  <S**2>=0.000
       7 -> 16        -0.12456
       8 -> 17         0.12454
       9 -> 18         0.67035
 This state for optimization and/or second-order correction.
 Total Energy, E(TD-HF/TD-DFT) =  -239.266572814 (259.47)

-1 1
                1         -0.009065    0.000000   -1.040599
                6          0.280861   -0.000000   -0.000000
                9          1.784465    0.000000    0.000000
                9         -1.960427   -0.000000   -0.000000
                1         -0.009054   -0.901187    0.520299
                1         -0.009054    0.901187    0.520299
```

```
$nbo file=fch3f_td_m6 archive plot nrt nrtlong $end
 $NRTSTR
   STR        ! Wgt=88.03%; rhoNL=0.10054; D(0)=0.03416
     LONE 3 3 4 4 END
     BOND S 1 2 S 2 3 S 2 5 S 2 6 END
   END
   STR        ! Wgt=8.78%; rhoNL=0.54057; D(0)=0.07890
     LONE 3 4 4 3 END
     BOND S 1 2 S 2 4 S 2 5 S 2 6 END
   END
   STR        ! Wgt=2.93%; rhoNL=1.54547; D(0)=0.13332
     LONE 2 1 3 3 4 3 END
     BOND S 1 2 S 2 5 S 2 6 S 3 4 END
   END
   STR        ! Wgt=53.92%; rhoNL=1.31552; D(0)=0.09401
     LONE 2 1 3 3 4 3 END
     BOND S 1 2 S 2 5 S 2 6 S 3 4 END
   END
   STR        ! Wgt=7.23%; rhoNL=2.93826; D(0)=0.16580
     LONE 2 1 3 3 4 3 6 1 END
     BOND S 1 2 S 2 4 S 2 5 END
   END
   STR        ! Wgt=7.23%; rhoNL=2.93826; D(0)=0.16580
     LONE 2 1 3 3 4 3 6 1 END
     BOND S 1 2 S 2 3 S 2 5 END
   END
   STR        ! Wgt=7.23%; rhoNL=2.93826; D(0)=0.16580
     LONE 2 1 3 3 4 3 5 1 END
     BOND S 1 2 S 2 3 S 2 6 END
   END
   STR        ! Wgt=7.23%; rhoNL=2.93826; D(0)=0.16580
     LONE 2 1 3 3 4 3 5 1 END
     BOND S 1 2 S 2 4 S 2 6 END
   END
   STR        ! Wgt=7.23%; rhoNL=2.93826; D(0)=0.16580
     LONE 1 1 2 1 3 3 4 3 END
     BOND S 2 4 S 2 5 S 2 6 END
   END
   STR        ! Wgt=7.23%; rhoNL=2.93826; D(0)=0.16580
     LONE 1 1 2 1 3 3 4 3 END
     BOND S 2 3 S 2 5 S 2 6 END
   END
 $END

--Link1--
%mem=2gb
%nprocshared=8
%chk=fch3f_m_td
#b3lyp/6-311++g** pop=nbo7read density=current td=(root=64,nstates=100)

F...CH3F, #m5, IRC=-1.76986, E = -239.697548469 (-10.97)
 Excited State  64:      Singlet-A'    11.3550 eV  109.19 nm  f=0.0960  <S**2>$
       7 -> 16         0.28288
       8 -> 17         0.28286
       9 -> 18         0.56902
 This state for optimization and/or second-order correction.
 Total Energy, E(TD-HF/TD-DFT) =  -239.280259318 (250.88)

-1 1
                1        -0.015161    0.000000   -1.048552
                6         0.235928    0.000000   -0.000001
                9         1.791858    0.000000    0.000001
                9        -1.938455    0.000000    0.000000
                1        -0.015153   -0.908073    0.524274
                1        -0.015153    0.908073    0.524274

$nbo file=fch3f_td_m5 archive plot nrt nrtlong $end
 $NRTSTR
   STR        ! Wgt=88.03%; rhoNL=0.10054; D(0)=0.03416
     LONE 3 3 4 4 END
     BOND S 1 2 S 2 3 S 2 5 S 2 6 END
```

```
   END
   STR        ! Wgt=8.78%; rhoNL=0.54057; D(0)=0.07890
     LONE 3 4 4 3 END
     BOND S 1 2 S 2 4 S 2 5 S 2 6 END
   END
   STR        ! Wgt=2.93%; rhoNL=1.54547; D(0)=0.13332
     LONE 2 1 3 3 4 3 END
     BOND S 1 2 S 2 5 S 2 6 S 3 4 END
   END
   STR        ! Wgt=53.92%; rhoNL=1.31552; D(0)=0.09401
     LONE 2 1 3 3 4 3 END
     BOND S 1 2 S 2 5 S 2 6 S 3 4 END
   END
   STR        ! Wgt=7.23%; rhoNL=2.93826; D(0)=0.16580
     LONE 2 1 3 3 4 3 6 1 END
     BOND S 1 2 S 2 4 S 2 5 END
   END
   STR        ! Wgt=7.23%; rhoNL=2.93826; D(0)=0.16580
     LONE 2 1 3 3 4 3 6 1 END
     BOND S 1 2 S 2 3 S 2 5 END
   END
   STR        ! Wgt=7.23%; rhoNL=2.93826; D(0)=0.16580
     LONE 2 1 3 3 4 3 5 1 END
     BOND S 1 2 S 2 3 S 2 6 END
   END
   STR        ! Wgt=7.23%; rhoNL=2.93826; D(0)=0.16580
     LONE 2 1 3 3 4 3 5 1 END
     BOND S 1 2 S 2 4 S 2 6 END
   END
   STR        ! Wgt=7.23%; rhoNL=2.93826; D(0)=0.16580
     LONE 1 1 2 1 3 3 4 3 END
     BOND S 2 4 S 2 5 S 2 6 END
   END
   STR        ! Wgt=7.23%; rhoNL=2.93826; D(0)=0.16580
     LONE 1 1 2 1 3 3 4 3 END
     BOND S 2 3 S 2 5 S 2 6 END
   END
 $END

--Link1--
%mem=2gb
%nprocshared=8
%chk=fch3f_m_td
#b3lyp/6-311++g** pop=nbo7read density=current td=(root=55,nstates=100)

F...CH3F, #m4, IRC=-1.41605, E = E(RB3LYP) =  -239.694883585 (-9.30)
 Excited State  55:      Singlet-A'    10.7344 eV  115.50 nm  f=0.1550  <S**2>$
       7 -> 16        -0.16734
       8 -> 17        -0.16735
       9 -> 18         0.64171
 This state for optimization and/or second-order correction.
 Total Energy, E(TD-HF/TD-DFT) =  -239.300402646 (238.24)

-1 1
                1         -0.018107   -0.000000   -1.057221
                6          0.189320    0.000000   -0.000002
                9          1.804013   -0.000000    0.000001
                9         -1.920720   -0.000000    0.000001
                1         -0.018102   -0.915580    0.528607
                1         -0.018102    0.915580    0.528607

$nbo file=fch3f_td_m4 archive plot nrt nrtlong $end
 $NRTSTR
   STR        ! Wgt=88.03%; rhoNL=0.10054; D(0)=0.03416
     LONE 3 3 4 4 END
     BOND S 1 2 S 2 3 S 2 5 S 2 6 END
   END
   STR        ! Wgt=8.78%; rhoNL=0.54057; D(0)=0.07890
     LONE 3 4 4 3 END
     BOND S 1 2 S 2 4 S 2 5 S 2 6 END
   END
```

```
   STR        ! Wgt=2.93%; rhoNL=1.54547; D(0)=0.13332
     LONE 2 1 3 3 4 3 END
     BOND S 1 2 S 2 5 S 2 6 S 3 4 END
   END
   STR        ! Wgt=53.92%; rhoNL=1.31552; D(0)=0.09401
     LONE 2 1 3 3 4 3 END
     BOND S 1 2 S 2 5 S 2 6 S 3 4 END
   END
   STR        ! Wgt=7.23%; rhoNL=2.93826; D(0)=0.16580
     LONE 2 1 3 3 4 3 6 1 END
     BOND S 1 2 S 2 4 S 2 5 END
   END
   STR        ! Wgt=7.23%; rhoNL=2.93826; D(0)=0.16580
     LONE 2 1 3 3 4 3 6 1 END
     BOND S 1 2 S 2 3 S 2 5 END
   END
   STR        ! Wgt=7.23%; rhoNL=2.93826; D(0)=0.16580
     LONE 2 1 3 3 4 3 5 1 END
     BOND S 1 2 S 2 3 S 2 6 END
   END
   STR        ! Wgt=7.23%; rhoNL=2.93826; D(0)=0.16580
     LONE 2 1 3 3 4 3 5 1 END
     BOND S 1 2 S 2 4 S 2 6 END
   END
   STR        ! Wgt=7.23%; rhoNL=2.93826; D(0)=0.16580
     LONE 1 1 2 1 3 3 4 3 END
     BOND S 2 4 S 2 5 S 2 6 END
   END
   STR        ! Wgt=7.23%; rhoNL=2.93826; D(0)=0.16580
     LONE 1 1 2 1 3 3 4 3 END
     BOND S 2 3 S 2 5 S 2 6 END
   END
 $END

--Link1--
%mem=2gb
%nprocshared=8
%chk=fch3f_m_td
#b3lyp/6-311++g** pop=nbo7read density=current td=(root=50,nstates=100)

F...CH3F, #m3, IRC=-1.06204, E = -239.692192320 (-7.61)
 Excited State  50:      Singlet-A'    10.2389 eV  121.09 nm  f=0.0332  <S**2>=$
       7 -> 17        -0.10672
       8 -> 16        -0.10672
       9 -> 18         0.53804
      12 -> 25        -0.21200
      13 -> 24         0.24199
      14 -> 23         0.24195
 This state for optimization and/or second-order correction.
 Total Energy, E(TD-HF/TD-DFT) =  -239.315920157 (228.50)

-1 1
                1         -0.016083    0.000000   -1.065313
                6          0.142177   -0.000000   -0.000002
                9          1.818172    0.000000    0.000001
                9         -1.905424    0.000000    0.000001
                1         -0.016078   -0.922586    0.532652
                1         -0.016078    0.922586    0.532652

$nbo file=fch3f_td_m3 archive plot nrt nrtlong $end
 $NRTSTR
   STR        ! Wgt=88.03%; rhoNL=0.10054; D(0)=0.03416
     LONE 3 3 4 4 END
     BOND S 1 2 S 2 3 S 2 5 S 2 6 END
   END
   STR        ! Wgt=8.78%; rhoNL=0.54057; D(0)=0.07890
     LONE 3 4 4 3 END
     BOND S 1 2 S 2 4 S 2 5 S 2 6 END
   END
   STR        ! Wgt=2.93%; rhoNL=1.54547; D(0)=0.13332
     LONE 2 1 3 3 4 3 END
```

```
    BOND S 1 2 S 2 5 S 2 6 S 3 4 END
  END
  STR        ! Wgt=53.92%; rhoNL=1.31552; D(0)=0.09401
    LONE 2 1 3 3 4 3 END
    BOND S 1 2 S 2 5 S 2 6 S 3 4 END
  END
  STR        ! Wgt=7.23%; rhoNL=2.93826; D(0)=0.16580
    LONE 2 1 3 3 4 3 6 1 END
    BOND S 1 2 S 2 4 S 2 5 END
  END
  STR        ! Wgt=7.23%; rhoNL=2.93826; D(0)=0.16580
    LONE 2 1 3 3 4 3 6 1 END
    BOND S 1 2 S 2 3 S 2 5 END
  END
  STR        ! Wgt=7.23%; rhoNL=2.93826; D(0)=0.16580
    LONE 2 1 3 3 4 3 5 1 END
    BOND S 1 2 S 2 3 S 2 6 END
  END
  STR        ! Wgt=7.23%; rhoNL=2.93826; D(0)=0.16580
    LONE 2 1 3 3 4 3 5 1 END
    BOND S 1 2 S 2 4 S 2 6 END
  END
  STR        ! Wgt=7.23%; rhoNL=2.93826; D(0)=0.16580
    LONE 1 1 2 1 3 3 4 3 END
    BOND S 2 4 S 2 5 S 2 6 END
  END
  STR        ! Wgt=7.23%; rhoNL=2.93826; D(0)=0.16580
    LONE 1 1 2 1 3 3 4 3 END
    BOND S 2 3 S 2 5 S 2 6 END
  END
 $END

--Link1--
%mem=2gb
%nprocshared=8
%chk=fch3f_m_td
#b3lyp/6-311++g** pop=nbo7read density=current td=(root=42,nstates=100)

F...CH3F, #m2, IRC=-0.70805, E = -239.689954267 (-6.21)
 Excited State  42:      Singlet-A'     9.7974 eV  126.55 nm  f=0.1582  <S**2>$
       9 -> 18         0.62459
      10 -> 20         0.15942
      11 -> 19         0.15942
      14 -> 22         0.10639
 This state for optimization and/or second-order correction.
 Total Energy, E(TD-HF/TD-DFT) =  -239.329907045 (219.73)

-1 1
                1         -0.012332   -0.000000   -1.070594
                6          0.094872    0.000000   -0.000002
                9          1.832996   -0.000000    0.000001
                9         -1.890962    0.000000    0.000001
                1         -0.012327   -0.927159    0.535293
                1         -0.012327    0.927159    0.535293

$nbo file=fch3f_td_m2 archive plot nrt nrtlong $end
 $NRTSTR
  STR        ! Wgt=88.03%; rhoNL=0.10054; D(0)=0.03416
    LONE 3 3 4 4 END
    BOND S 1 2 S 2 3 S 2 5 S 2 6 END
  END
  STR        ! Wgt=8.78%; rhoNL=0.54057; D(0)=0.07890
    LONE 3 4 4 3 END
    BOND S 1 2 S 2 4 S 2 5 S 2 6 END
  END
  STR        ! Wgt=2.93%; rhoNL=1.54547; D(0)=0.13332
    LONE 2 1 3 3 4 3 END
    BOND S 1 2 S 2 5 S 2 6 S 3 4 END
  END
  STR        ! Wgt=53.92%; rhoNL=1.31552; D(0)=0.09401
    LONE 2 1 3 3 4 3 END
```

```
    BOND S 1 2 S 2 5 S 2 6 S 3 4 END
  END
  STR        ! Wgt=7.23%; rhoNL=2.93826; D(0)=0.16580
    LONE 2 1 3 3 4 3 6 1 END
    BOND S 1 2 S 2 4 S 2 5 END
  END
  STR        ! Wgt=7.23%; rhoNL=2.93826; D(0)=0.16580
    LONE 2 1 3 3 4 3 6 1 END
    BOND S 1 2 S 2 3 S 2 5 END
  END
  STR        ! Wgt=7.23%; rhoNL=2.93826; D(0)=0.16580
    LONE 2 1 3 3 4 3 5 1 END
    BOND S 1 2 S 2 3 S 2 6 END
  END
  STR        ! Wgt=7.23%; rhoNL=2.93826; D(0)=0.16580
    LONE 2 1 3 3 4 3 5 1 END
    BOND S 1 2 S 2 4 S 2 6 END
  END
  STR        ! Wgt=7.23%; rhoNL=2.93826; D(0)=0.16580
    LONE 1 1 2 1 3 3 4 3 END
    BOND S 2 4 S 2 5 S 2 6 END
  END
  STR        ! Wgt=7.23%; rhoNL=2.93826; D(0)=0.16580
    LONE 1 1 2 1 3 3 4 3 END
    BOND S 2 3 S 2 5 S 2 6 END
  END
 $END

--Link1--
%mem=2gb
%nprocshared=8
%chk=fch3f_m_td
#b3lyp/6-311++g** pop=nbo7read density=current td=(root=42,nstates=100)

F...CH3F, #m1, IRC=-0.35407, E = -239.688496623 (-5.29)
 Excited State  42:      Singlet-A'     9.5024 eV  130.48 nm  f=0.3189  <S**2>$
       9 -> 18         0.47044
       9 -> 21         0.10485
      10 -> 19         0.21344
      11 -> 20        -0.21344
      14 -> 21         0.33670
      14 -> 22        -0.13521
 This state for optimization and/or second-order correction.
 Total Energy, E(TD-HF/TD-DFT) =  -239.339288239 (213.84)

-1 1
                1         -0.006549    0.000000   -1.072800
                6          0.047465    0.000000   -0.000000
                9          1.847731   -0.000000    0.000000
                9         -1.876669   -0.000000   -0.000000
                1         -0.006547   -0.929072    0.536400
                1         -0.006547    0.929072    0.536400

$nbo file=fch3f_td_m1 archive plot nrt nrtlong $end
 $NRTSTR
  STR        ! Wgt=88.03%; rhoNL=0.10054; D(0)=0.03416
    LONE 3 3 4 4 END
    BOND S 1 2 S 2 3 S 2 5 S 2 6 END
  END
  STR        ! Wgt=8.78%; rhoNL=0.54057; D(0)=0.07890
    LONE 3 4 4 3 END
    BOND S 1 2 S 2 4 S 2 5 S 2 6 END
  END
  STR        ! Wgt=2.93%; rhoNL=1.54547; D(0)=0.13332
    LONE 2 1 3 3 4 3 END
    BOND S 1 2 S 2 5 S 2 6 S 3 4 END
  END
  STR        ! Wgt=53.92%; rhoNL=1.31552; D(0)=0.09401
    LONE 2 1 3 3 4 3 END
    BOND S 1 2 S 2 5 S 2 6 S 3 4 END
  END
```

```
   STR        ! Wgt=7.23%; rhoNL=2.93826; D(0)=0.16580
     LONE 2 1 3 3 4 3 6 1 END
     BOND S 1 2 S 2 4 S 2 5 END
   END
   STR        ! Wgt=7.23%; rhoNL=2.93826; D(0)=0.16580
     LONE 2 1 3 3 4 3 6 1 END
     BOND S 1 2 S 2 3 S 2 5 END
   END
   STR        ! Wgt=7.23%; rhoNL=2.93826; D(0)=0.16580
     LONE 2 1 3 3 4 3 5 1 END
     BOND S 1 2 S 2 3 S 2 6 END
   END
   STR        ! Wgt=7.23%; rhoNL=2.93826; D(0)=0.16580
     LONE 2 1 3 3 4 3 5 1 END
     BOND S 1 2 S 2 4 S 2 6 END
   END
   STR        ! Wgt=7.23%; rhoNL=2.93826; D(0)=0.16580
     LONE 1 1 2 1 3 3 4 3 END
     BOND S 2 4 S 2 5 S 2 6 END
   END
   STR        ! Wgt=7.23%; rhoNL=2.93826; D(0)=0.16580
     LONE 1 1 2 1 3 3 4 3 END
     BOND S 2 3 S 2 5 S 2 6 END
   END
 $END

--Link1--
%mem=2gb
%nprocshared=8
%chk=fch3f_m_td
#b3lyp/6-311++g** pop=nbo7read density=current td=(root=41,nstates=100)

F...CH3F  SN2 t.s., IRC = 0.00, E(RB3LYP) =  -239.687990417 (-4.97)
[F(-) + CH3F: -239.6800656]
nu: -465,314(2),342,1043(2),1200,1389(2),3155,3346(2)
 Vibrational temperatures:    451.12   451.12   492.69  1501.26  1501.26
          (Kelvin)           1727.70  1997.85  1997.85  4539.86  4814.50
                             4814.50
 Zero-point correction=                           0.038460 (Hartree/Particle)
 Thermal correction to Energy=                    0.042564
 Thermal correction to Enthalpy=                  0.043508
 Thermal correction to Gibbs Free Energy=         0.014548
 Sum of electronic and zero-point Energies=           -239.649530
 Sum of electronic and thermal Energies=              -239.645426
 Sum of electronic and thermal Enthalpies=            -239.644482
 Sum of electronic and thermal Free Energies=         -239.673442
 Excited State  41:      Singlet-A1'    9.2812 eV  133.59 nm  f=0.0000  <S**2>=$
       9 -> 18         0.67262
       9 -> 21         0.10687
 This state for optimization and/or second-order correction.
 Total Energy, E(TD-HF/TD-DFT) =  -239.346912833 (209.06)

-1  1
H
C    1   r2
F    2   r3    1  90.
F    2   r3    1  90.   3   180.
H    2   r2    3  90.   1   120.
H    2   r2    3  90.   1  -120.

r2  1.0728
r3  1.8622

$nbo file=fch3f_td_m_ts archive archive plot nrt nrtlong $end
 $NRTSTR
   STR        ! Wgt=88.03%; rhoNL=0.10054; D(0)=0.03416
     LONE 3 3 4 4 END
     BOND S 1 2 S 2 3 S 2 5 S 2 6 END
   END
   STR        ! Wgt=8.78%; rhoNL=0.54057; D(0)=0.07890
     LONE 3 4 4 3 END
```

```
      BOND S 1 2 S 2 4 S 2 5 S 2 6 END
    END
    STR        ! Wgt=2.93%; rhoNL=1.54547; D(0)=0.13332
      LONE 2 1 3 3 4 3 END
      BOND S 1 2 S 2 5 S 2 6 S 3 4 END
    END
    STR        ! Wgt=53.92%; rhoNL=1.31552; D(0)=0.09401
      LONE 2 1 3 3 4 3 END
      BOND S 1 2 S 2 5 S 2 6 S 3 4 END
    END
    STR        ! Wgt=7.23%; rhoNL=2.93826; D(0)=0.16580
      LONE 2 1 3 3 4 3 6 1 END
      BOND S 1 2 S 2 4 S 2 5 END
    END
    STR        ! Wgt=7.23%; rhoNL=2.93826; D(0)=0.16580
      LONE 2 1 3 3 4 3 6 1 END
      BOND S 1 2 S 2 3 S 2 5 END
    END
    STR        ! Wgt=7.23%; rhoNL=2.93826; D(0)=0.16580
      LONE 2 1 3 3 4 3 5 1 END
      BOND S 1 2 S 2 3 S 2 6 END
    END
    STR        ! Wgt=7.23%; rhoNL=2.93826; D(0)=0.16580
      LONE 2 1 3 3 4 3 5 1 END
      BOND S 1 2 S 2 4 S 2 6 END
    END
    STR        ! Wgt=7.23%; rhoNL=2.93826; D(0)=0.16580
      LONE 1 1 2 1 3 3 4 3 END
      BOND S 2 4 S 2 5 S 2 6 END
    END
    STR        ! Wgt=7.23%; rhoNL=2.93826; D(0)=0.16580
      LONE 1 1 2 1 3 3 4 3 END
      BOND S 2 3 S 2 5 S 2 6 END
    END
  $END

--Link1--
%mem=2gb
%nprocshared=8
%chk=fch3f_m_td
#b3lyp/6-311++g** pop=nbo7read density=current td=(root=42,nstates=100)

F...CH3F, #p1, IRC=0.35408, E = E(RB3LYP) =  -239.688496623 (-5.29)
 Excited State  42:      Singlet-A'     9.5024 eV  130.48 nm  f=0.3189  <S**2>$
       9 -> 18         0.47044
       9 -> 21         0.10485
      10 -> 19         0.21344
      11 -> 20         0.21344
      14 -> 21         0.33670
      14 -> 22        -0.13521
 This state for optimization and/or second-order correction.
 Total Energy, E(TD-HF/TD-DFT) =  -239.339288239 (213.84)

-1 1
                1          0.006549   -0.000000   -1.072800
                6         -0.047465   -0.000000    0.000000
                9          1.876669    0.000000   -0.000000
                9         -1.847731    0.000000    0.000000
                1          0.006547   -0.929072    0.536400
                1          0.006547    0.929072    0.536400

$nbo file=fch3f_td_p1 archive plot nrt nrtlong $end
 $NRTSTR
    STR        ! Wgt=88.03%; rhoNL=0.10054; D(0)=0.03416
      LONE 3 3 4 4 END
      BOND S 1 2 S 2 3 S 2 5 S 2 6 END
    END
    STR        ! Wgt=8.78%; rhoNL=0.54057; D(0)=0.07890
      LONE 3 4 4 3 END
      BOND S 1 2 S 2 4 S 2 5 S 2 6 END
    END
```

```
   STR        ! Wgt=2.93%; rhoNL=1.54547; D(0)=0.13332
     LONE 2 1 3 3 4 3 END
     BOND S 1 2 S 2 5 S 2 6 S 3 4 END
   END
   STR        ! Wgt=53.92%; rhoNL=1.31552; D(0)=0.09401
     LONE 2 1 3 3 4 3 END
     BOND S 1 2 S 2 5 S 2 6 S 3 4 END
   END
   STR        ! Wgt=7.23%; rhoNL=2.93826; D(0)=0.16580
     LONE 2 1 3 3 4 3 6 1 END
     BOND S 1 2 S 2 4 S 2 5 END
   END
   STR        ! Wgt=7.23%; rhoNL=2.93826; D(0)=0.16580
     LONE 2 1 3 3 4 3 6 1 END
     BOND S 1 2 S 2 3 S 2 5 END
   END
   STR        ! Wgt=7.23%; rhoNL=2.93826; D(0)=0.16580
     LONE 2 1 3 3 4 3 5 1 END
     BOND S 1 2 S 2 3 S 2 6 END
   END
   STR        ! Wgt=7.23%; rhoNL=2.93826; D(0)=0.16580
     LONE 2 1 3 3 4 3 5 1 END
     BOND S 1 2 S 2 4 S 2 6 END
   END
   STR        ! Wgt=7.23%; rhoNL=2.93826; D(0)=0.16580
     LONE 1 1 2 1 3 3 4 3 END
     BOND S 2 4 S 2 5 S 2 6 END
   END
   STR        ! Wgt=7.23%; rhoNL=2.93826; D(0)=0.16580
     LONE 1 1 2 1 3 3 4 3 END
     BOND S 2 3 S 2 5 S 2 6 END
   END
 $END

--Link1--
%mem=2gb
%nprocshared=8
%chk=fch3f_m_td
#b3lyp/6-311++g** pop=nbo7read density=current td=(root=42,nstates=100)

F...CH3F, #p2, IRC=0.70806, E = E(RB3LYP) =  -239.689954308 (-6.21)
 Excited State  42:      Singlet-A'     9.7974 eV  126.55 nm  f=0.1582  <S**2>$
       9 -> 18         0.62459
      10 -> 20        -0.15942
      11 -> 19         0.15941
      14 -> 22         0.10639
 This state for optimization and/or second-order correction.
 Total Energy, E(TD-HF/TD-DFT) =  -239.329906397 (219.73)

-1 1
                1          0.012332   -0.000000   -1.070595
                6         -0.094873   -0.000000   -0.000003
                9          1.890960    0.000000    0.000001
                9         -1.832993    0.000000    0.000001
                1          0.012327   -0.927159    0.535292
                1          0.012327    0.927159    0.535292

$nbo file=fch3f_td_p2 archive plot nrt nrtlong $end
 $NRTSTR
   STR        ! Wgt=88.03%; rhoNL=0.10054; D(0)=0.03416
     LONE 3 3 4 4 END
     BOND S 1 2 S 2 3 S 2 5 S 2 6 END
   END
   STR        ! Wgt=8.78%; rhoNL=0.54057; D(0)=0.07890
     LONE 3 4 4 3 END
     BOND S 1 2 S 2 4 S 2 5 S 2 6 END
   END
   STR        ! Wgt=2.93%; rhoNL=1.54547; D(0)=0.13332
     LONE 2 1 3 3 4 3 END
     BOND S 1 2 S 2 5 S 2 6 S 3 4 END
   END
```

```
   STR        ! Wgt=53.92%; rhoNL=1.31552; D(0)=0.09401
     LONE 2 1 3 3 4 3 END
     BOND S 1 2 S 2 5 S 2 6 S 3 4 END
   END
   STR        ! Wgt=7.23%; rhoNL=2.93826; D(0)=0.16580
     LONE 2 1 3 3 4 3 6 1 END
     BOND S 1 2 S 2 4 S 2 5 END
   END
   STR        ! Wgt=7.23%; rhoNL=2.93826; D(0)=0.16580
     LONE 2 1 3 3 4 3 6 1 END
     BOND S 1 2 S 2 3 S 2 5 END
   END
   STR        ! Wgt=7.23%; rhoNL=2.93826; D(0)=0.16580
     LONE 2 1 3 3 4 3 5 1 END
     BOND S 1 2 S 2 3 S 2 6 END
   END
   STR        ! Wgt=7.23%; rhoNL=2.93826; D(0)=0.16580
     LONE 2 1 3 3 4 3 5 1 END
     BOND S 1 2 S 2 4 S 2 6 END
   END
   STR        ! Wgt=7.23%; rhoNL=2.93826; D(0)=0.16580
     LONE 1 1 2 1 3 3 4 3 END
     BOND S 2 4 S 2 5 S 2 6 END
   END
   STR        ! Wgt=7.23%; rhoNL=2.93826; D(0)=0.16580
     LONE 1 1 2 1 3 3 4 3 END
     BOND S 2 3 S 2 5 S 2 6 END
   END
 $END

--Link1--
%mem=2gb
%nprocshared=8
%chk=fch3f_m_td
#b3lyp/6-311++g** pop=nbo7read density=current td=(root=50,nstates=100)

F...CH3F, #p3, IRC=1.06205, E = -239.692192374 (-7.61)
 Excited State  50:      Singlet-A'    10.2389 eV  121.09 nm  f=0.0332  <S**2>=$
       7 -> 17        -0.10672
       8 -> 16         0.10672
       9 -> 18         0.53806
      12 -> 25        -0.21203
      13 -> 24         0.24195
      14 -> 23        -0.24191
 This state for optimization and/or second-order correction.
 Total Energy, E(TD-HF/TD-DFT) =  -239.315919811 (228.51)

-1 1
                1          0.016083    0.000000   -1.065313
                6         -0.142178    0.000000   -0.000003
                9          1.905423   -0.000000    0.000001
                9         -1.818170   -0.000000    0.000001
                1          0.016078   -0.922585    0.532651
                1          0.016078    0.922585    0.532651

$nbo file=fch3f_td_p3 archive plot nrt nrtlong $end
 $NRTSTR
   STR        ! Wgt=88.03%; rhoNL=0.10054; D(0)=0.03416
     LONE 3 3 4 4 END
     BOND S 1 2 S 2 3 S 2 5 S 2 6 END
   END
   STR        ! Wgt=8.78%; rhoNL=0.54057; D(0)=0.07890
     LONE 3 4 4 3 END
     BOND S 1 2 S 2 4 S 2 5 S 2 6 END
   END
   STR        ! Wgt=2.93%; rhoNL=1.54547; D(0)=0.13332
     LONE 2 1 3 3 4 3 END
     BOND S 1 2 S 2 5 S 2 6 S 3 4 END
   END
   STR        ! Wgt=53.92%; rhoNL=1.31552; D(0)=0.09401
     LONE 2 1 3 3 4 3 END
```

```
    BOND S 1 2 S 2 5 S 2 6 S 3 4 END
  END
  STR        ! Wgt=7.23%; rhoNL=2.93826; D(0)=0.16580
    LONE 2 1 3 3 4 3 6 1 END
    BOND S 1 2 S 2 4 S 2 5 END
  END
  STR        ! Wgt=7.23%; rhoNL=2.93826; D(0)=0.16580
    LONE 2 1 3 3 4 3 6 1 END
    BOND S 1 2 S 2 3 S 2 5 END
  END
  STR        ! Wgt=7.23%; rhoNL=2.93826; D(0)=0.16580
    LONE 2 1 3 3 4 3 5 1 END
    BOND S 1 2 S 2 3 S 2 6 END
  END
  STR        ! Wgt=7.23%; rhoNL=2.93826; D(0)=0.16580
    LONE 2 1 3 3 4 3 5 1 END
    BOND S 1 2 S 2 4 S 2 6 END
  END
  STR        ! Wgt=7.23%; rhoNL=2.93826; D(0)=0.16580
    LONE 1 1 2 1 3 3 4 3 END
    BOND S 2 4 S 2 5 S 2 6 END
  END
  STR        ! Wgt=7.23%; rhoNL=2.93826; D(0)=0.16580
    LONE 1 1 2 1 3 3 4 3 END
    BOND S 2 3 S 2 5 S 2 6 END
  END
 $END

--Link1--
%mem=2gb
%nprocshared=8
%chk=fch3f_m_td
#b3lyp/6-311++g** pop=nbo7read density=current td=(root=55,nstates=100)

F...CH3F, #p4, IRC=1.41605, E = -239.694883640 (-9.30)
 Excited State  55:      Singlet-A'    10.7344 eV  115.50 nm  f=0.1550  <S**2>$
       7 -> 16        -0.16734
       8 -> 17         0.16735
       9 -> 18         0.64171
 This state for optimization and/or second-order correction.
 Total Energy, E(TD-HF/TD-DFT) =  -239.300402248 (238.24)

-1 1
                1          0.018107    0.000000   -1.057221
                6         -0.189321   -0.000000   -0.000002
                9          1.920719    0.000000    0.000001
                9         -1.804012    0.000000    0.000001
                1          0.018102   -0.915580    0.528607
                1          0.018102    0.915580    0.528607

$nbo file=fch3f_td_p4 archive plot nrt nrtlong $end
 $NRTSTR
  STR        ! Wgt=88.03%; rhoNL=0.10054; D(0)=0.03416
    LONE 3 3 4 4 END
    BOND S 1 2 S 2 3 S 2 5 S 2 6 END
  END
  STR        ! Wgt=8.78%; rhoNL=0.54057; D(0)=0.07890
    LONE 3 4 4 3 END
    BOND S 1 2 S 2 4 S 2 5 S 2 6 END
  END
  STR        ! Wgt=2.93%; rhoNL=1.54547; D(0)=0.13332
    LONE 2 1 3 3 4 3 END
    BOND S 1 2 S 2 5 S 2 6 S 3 4 END
  END
  STR        ! Wgt=53.92%; rhoNL=1.31552; D(0)=0.09401
    LONE 2 1 3 3 4 3 END
    BOND S 1 2 S 2 5 S 2 6 S 3 4 END
  END
  STR        ! Wgt=7.23%; rhoNL=2.93826; D(0)=0.16580
    LONE 2 1 3 3 4 3 6 1 END
    BOND S 1 2 S 2 4 S 2 5 END
```

```
   END
   STR        ! Wgt=7.23%; rhoNL=2.93826; D(0)=0.16580
     LONE 2 1 3 3 4 3 6 1 END
     BOND S 1 2 S 2 3 S 2 5 END
   END
   STR        ! Wgt=7.23%; rhoNL=2.93826; D(0)=0.16580
     LONE 2 1 3 3 4 3 5 1 END
     BOND S 1 2 S 2 3 S 2 6 END
   END
   STR        ! Wgt=7.23%; rhoNL=2.93826; D(0)=0.16580
     LONE 2 1 3 3 4 3 5 1 END
     BOND S 1 2 S 2 4 S 2 6 END
   END
   STR        ! Wgt=7.23%; rhoNL=2.93826; D(0)=0.16580
     LONE 1 1 2 1 3 3 4 3 END
     BOND S 2 4 S 2 5 S 2 6 END
   END
   STR        ! Wgt=7.23%; rhoNL=2.93826; D(0)=0.16580
     LONE 1 1 2 1 3 3 4 3 END
     BOND S 2 3 S 2 5 S 2 6 END
   END
 $END

--Link1--
%mem=2gb
%nprocshared=8
%chk=fch3f_m_td
#b3lyp/6-311++g** pop=nbo7read density=current td=(root=64,nstates=100)

F...CH3F, #p5, IRC=1.76987, E = -239.697548509 (-10.97)
 Excited State  64:      Singlet-A'    11.3550 eV  109.19 nm  f=0.0960  <S**2>$
       7 -> 16         0.28288
       8 -> 17         0.28286
       9 -> 18         0.56902
 This state for optimization and/or second-order correction.
 Total Energy, E(TD-HF/TD-DFT) =  -239.280259318 (250.88)

-1 1
                1          0.015161    0.000000   -1.048552
                6         -0.235929    0.000000   -0.000002
                9          1.938454   -0.000000    0.000000
                9         -1.791857   -0.000000    0.000001
                1          0.015154   -0.908073    0.524273
                1          0.015154    0.908073    0.524273

$nbo file=fch3f_td_p5 archive plot nrt nrtlong $end
 $NRTSTR
   STR        ! Wgt=88.03%; rhoNL=0.10054; D(0)=0.03416
     LONE 3 3 4 4 END
     BOND S 1 2 S 2 3 S 2 5 S 2 6 END
   END
   STR        ! Wgt=8.78%; rhoNL=0.54057; D(0)=0.07890
     LONE 3 4 4 3 END
     BOND S 1 2 S 2 4 S 2 5 S 2 6 END
   END
   STR        ! Wgt=2.93%; rhoNL=1.54547; D(0)=0.13332
     LONE 2 1 3 3 4 3 END
     BOND S 1 2 S 2 5 S 2 6 S 3 4 END
   END
   STR        ! Wgt=53.92%; rhoNL=1.31552; D(0)=0.09401
     LONE 2 1 3 3 4 3 END
     BOND S 1 2 S 2 5 S 2 6 S 3 4 END
   END
   STR        ! Wgt=7.23%; rhoNL=2.93826; D(0)=0.16580
     LONE 2 1 3 3 4 3 6 1 END
     BOND S 1 2 S 2 4 S 2 5 END
   END
   STR        ! Wgt=7.23%; rhoNL=2.93826; D(0)=0.16580
     LONE 2 1 3 3 4 3 6 1 END
     BOND S 1 2 S 2 3 S 2 5 END
   END
```

```
   STR        ! Wgt=7.23%; rhoNL=2.93826; D(0)=0.16580
     LONE 2 1 3 3 4 3 5 1 END
     BOND S 1 2 S 2 3 S 2 6 END
   END
   STR        ! Wgt=7.23%; rhoNL=2.93826; D(0)=0.16580
     LONE 2 1 3 3 4 3 5 1 END
     BOND S 1 2 S 2 4 S 2 6 END
   END
   STR        ! Wgt=7.23%; rhoNL=2.93826; D(0)=0.16580
     LONE 1 1 2 1 3 3 4 3 END
     BOND S 2 4 S 2 5 S 2 6 END
   END
   STR        ! Wgt=7.23%; rhoNL=2.93826; D(0)=0.16580
     LONE 1 1 2 1 3 3 4 3 END
     BOND S 2 3 S 2 5 S 2 6 END
   END
 $END

--Link1--
%mem=2gb
%nprocshared=8
%chk=fch3f_m_td
#b3lyp/6-311++g** pop=nbo7read density=current td=(root=67,nstates=100)

F...CH3F, #p6, IRC=2.12229, E = -239.699653902 (-12.29)
 Excited State  67:      Singlet-A'    11.7847 eV  105.21 nm  f=0.1106  <S**2>=0.000
       7 -> 16        -0.12456
       8 -> 17         0.12454
       9 -> 18         0.67035
 This state for optimization and/or second-order correction.
 Total Energy, E(TD-HF/TD-DFT) =  -239.266572814 (259.47)

-1 1
                1          0.009064    0.000000   -1.040599
                6         -0.280861    0.000000   -0.000001
                9          1.960426   -0.000000   -0.000000
                9         -1.784464   -0.000000    0.000001
                1          0.009054   -0.901187    0.520299
                1          0.009054    0.901187    0.520299

$nbo file=fch3f_td_p6 archive plot nrt nrtlong $end
 $NRTSTR
   STR        ! Wgt=88.03%; rhoNL=0.10054; D(0)=0.03416
     LONE 3 3 4 4 END
     BOND S 1 2 S 2 3 S 2 5 S 2 6 END
   END
   STR        ! Wgt=8.78%; rhoNL=0.54057; D(0)=0.07890
     LONE 3 4 4 3 END
     BOND S 1 2 S 2 4 S 2 5 S 2 6 END
   END
   STR        ! Wgt=2.93%; rhoNL=1.54547; D(0)=0.13332
     LONE 2 1 3 3 4 3 END
     BOND S 1 2 S 2 5 S 2 6 S 3 4 END
   END
   STR        ! Wgt=53.92%; rhoNL=1.31552; D(0)=0.09401
     LONE 2 1 3 3 4 3 END
     BOND S 1 2 S 2 5 S 2 6 S 3 4 END
   END
   STR        ! Wgt=7.23%; rhoNL=2.93826; D(0)=0.16580
     LONE 2 1 3 3 4 3 6 1 END
     BOND S 1 2 S 2 4 S 2 5 END
   END
   STR        ! Wgt=7.23%; rhoNL=2.93826; D(0)=0.16580
     LONE 2 1 3 3 4 3 6 1 END
     BOND S 1 2 S 2 3 S 2 5 END
   END
   STR        ! Wgt=7.23%; rhoNL=2.93826; D(0)=0.16580
     LONE 2 1 3 3 4 3 5 1 END
     BOND S 1 2 S 2 3 S 2 6 END
   END
   STR        ! Wgt=7.23%; rhoNL=2.93826; D(0)=0.16580
```

```
     LONE 2 1 3 3 4 3 5 1 END
     BOND S 1 2 S 2 4 S 2 6 END
   END
   STR        ! Wgt=7.23%; rhoNL=2.93826; D(0)=0.16580
     LONE 1 1 2 1 3 3 4 3 END
     BOND S 2 4 S 2 5 S 2 6 END
   END
   STR        ! Wgt=7.23%; rhoNL=2.93826; D(0)=0.16580
     LONE 1 1 2 1 3 3 4 3 END
     BOND S 2 3 S 2 5 S 2 6 END
   END
 $END

--Link1--
%mem=2gb
%nprocshared=8
%chk=fch3f_m_td
#b3lyp/6-311++g** pop=nbo7read density=current td=(root=70,nstates=100)

F...CH3F, #p7, IRC=2.47108, E = -239.701020545 (-13.15)
 Excited State  70:      Singlet-A'    12.0864 eV  102.58 nm  f=0.1582  <S**2>$-       9 -> 18
0.68396
 This state for optimization and/or second-order correction.
 Total Energy, E(TD-HF/TD-DFT) =  -239.256851767 (265.57)

-1 1
                1         -0.003582   -0.000000   -1.035330
                6         -0.316308   -0.000000    0.000000
                9          1.991152    0.000000    0.000000
                9         -1.790746    0.000000   -0.000001
                1         -0.003598   -0.896626    0.517667
                1         -0.003598    0.896626    0.517667

$nbo file=fch3f_td_p7 archive plot nrt nrtlong $end
 $NRTSTR
   STR        ! Wgt=88.03%; rhoNL=0.10054; D(0)=0.03416
     LONE 3 3 4 4 END
     BOND S 1 2 S 2 3 S 2 5 S 2 6 END
   END
   STR        ! Wgt=8.78%; rhoNL=0.54057; D(0)=0.07890
     LONE 3 4 4 3 END
     BOND S 1 2 S 2 4 S 2 5 S 2 6 END
   END
   STR        ! Wgt=2.93%; rhoNL=1.54547; D(0)=0.13332
     LONE 2 1 3 3 4 3 END
     BOND S 1 2 S 2 5 S 2 6 S 3 4 END
   END
   STR        ! Wgt=53.92%; rhoNL=1.31552; D(0)=0.09401
     LONE 2 1 3 3 4 3 END
     BOND S 1 2 S 2 5 S 2 6 S 3 4 END
   END
   STR        ! Wgt=7.23%; rhoNL=2.93826; D(0)=0.16580
     LONE 2 1 3 3 4 3 6 1 END
     BOND S 1 2 S 2 4 S 2 5 END
   END
   STR        ! Wgt=7.23%; rhoNL=2.93826; D(0)=0.16580
     LONE 2 1 3 3 4 3 6 1 END
     BOND S 1 2 S 2 3 S 2 5 END
   END
   STR        ! Wgt=7.23%; rhoNL=2.93826; D(0)=0.16580
     LONE 2 1 3 3 4 3 5 1 END
     BOND S 1 2 S 2 3 S 2 6 END
   END
   STR        ! Wgt=7.23%; rhoNL=2.93826; D(0)=0.16580
     LONE 2 1 3 3 4 3 5 1 END
     BOND S 1 2 S 2 4 S 2 6 END
   END
   STR        ! Wgt=7.23%; rhoNL=2.93826; D(0)=0.16580
     LONE 1 1 2 1 3 3 4 3 END
     BOND S 2 4 S 2 5 S 2 6 END
   END
```

```
   STR        ! Wgt=7.23%; rhoNL=2.93826; D(0)=0.16580
     LONE 1 1 2 1 3 3 4 3 END
     BOND S 2 3 S 2 5 S 2 6 END
   END
 $END

--Link1--
%mem=2gb
%nprocshared=8
%chk=fch3f_m_td
#b3lyp/6-311++g** pop=nbo7read density=current td=(root=72,nstates=100)

F...CH3F, #p8, IRC=2.82422, E = E(RB3LYP) =  -239.701949250 (-13.73)
 Excited State  72:      Singlet-A'    12.2343 eV  101.34 nm  f=0.1765  <S**2>$
       7 -> 18         0.62920
      12 -> 29        -0.27415
 This state for optimization and/or second-order correction.
 Total Energy, E(TD-HF/TD-DFT) =  -239.252346940 (268.40)

-1 1
                1         -0.016736    0.000000   -1.032353
                6         -0.342193    0.000000    0.000001
                9          2.025094   -0.000000    0.000001
                9         -1.806184   -0.000000   -0.000003
                1         -0.016759   -0.894052    0.516181
                1         -0.016759    0.894052    0.516181

$nbo file=fch3f_td_p8 archive plot nrt nrtlong $end
 $NRTSTR
   STR        ! Wgt=88.03%; rhoNL=0.10054; D(0)=0.03416
     LONE 3 3 4 4 END
     BOND S 1 2 S 2 3 S 2 5 S 2 6 END
   END
   STR        ! Wgt=8.78%; rhoNL=0.54057; D(0)=0.07890
     LONE 3 4 4 3 END
     BOND S 1 2 S 2 4 S 2 5 S 2 6 END
   END
   STR        ! Wgt=2.93%; rhoNL=1.54547; D(0)=0.13332
     LONE 2 1 3 3 4 3 END
     BOND S 1 2 S 2 5 S 2 6 S 3 4 END
   END
   STR        ! Wgt=53.92%; rhoNL=1.31552; D(0)=0.09401
     LONE 2 1 3 3 4 3 END
     BOND S 1 2 S 2 5 S 2 6 S 3 4 END
   END
   STR        ! Wgt=7.23%; rhoNL=2.93826; D(0)=0.16580
     LONE 2 1 3 3 4 3 6 1 END
     BOND S 1 2 S 2 4 S 2 5 END
   END
   STR        ! Wgt=7.23%; rhoNL=2.93826; D(0)=0.16580
     LONE 2 1 3 3 4 3 6 1 END
     BOND S 1 2 S 2 3 S 2 5 END
   END
   STR        ! Wgt=7.23%; rhoNL=2.93826; D(0)=0.16580
     LONE 2 1 3 3 4 3 5 1 END
     BOND S 1 2 S 2 3 S 2 6 END
   END
   STR        ! Wgt=7.23%; rhoNL=2.93826; D(0)=0.16580
     LONE 2 1 3 3 4 3 5 1 END
     BOND S 1 2 S 2 4 S 2 6 END
   END
   STR        ! Wgt=7.23%; rhoNL=2.93826; D(0)=0.16580
     LONE 1 1 2 1 3 3 4 3 END
     BOND S 2 4 S 2 5 S 2 6 END
   END
   STR        ! Wgt=7.23%; rhoNL=2.93826; D(0)=0.16580
     LONE 1 1 2 1 3 3 4 3 END
     BOND S 2 3 S 2 5 S 2 6 END
   END
 $END
```

```
--Link1--
%mem=2gb
%nprocshared=8
%chk=fch3f_m_td
#b3lyp/6-311++g** pop=nbo7read density=current td=(root=73,nstates=100)

F...CH3F, #p9, IRC=3.17830, E = -239.702585779 (-14.13)
 Excited State  73:      Singlet-A'    12.3336 eV  100.53 nm  f=0.1166  <S**2>$
       7 -> 18         0.66811
      12 -> 29         0.15409
 This state for optimization and/or second-order correction.
 Total Energy, E(TD-HF/TD-DFT) =  -239.249333852 (270.29)

-1 1
                1         -0.032998   -0.000000   -1.031249
                6         -0.362713   -0.000000   -0.000003
                9          2.059356    0.000000    0.000009
                9         -1.825367    0.000000   -0.000006
                1         -0.033019   -0.893093    0.515620
                1         -0.033019    0.893093    0.515620

$nbo file=fch3f_td_p9 archive plot nrt nrtlong $end
 $NRTSTR
   STR        ! Wgt=88.03%; rhoNL=0.10054; D(0)=0.03416
     LONE 3 3 4 4 END
     BOND S 1 2 S 2 3 S 2 5 S 2 6 END
   END
   STR        ! Wgt=8.78%; rhoNL=0.54057; D(0)=0.07890
     LONE 3 4 4 3 END
     BOND S 1 2 S 2 4 S 2 5 S 2 6 END
   END
   STR        ! Wgt=2.93%; rhoNL=1.54547; D(0)=0.13332
     LONE 2 1 3 3 4 3 END
     BOND S 1 2 S 2 5 S 2 6 S 3 4 END
   END
   STR        ! Wgt=53.92%; rhoNL=1.31552; D(0)=0.09401
     LONE 2 1 3 3 4 3 END
     BOND S 1 2 S 2 5 S 2 6 S 3 4 END
   END
   STR        ! Wgt=7.23%; rhoNL=2.93826; D(0)=0.16580
     LONE 2 1 3 3 4 3 6 1 END
     BOND S 1 2 S 2 4 S 2 5 END
   END
   STR        ! Wgt=7.23%; rhoNL=2.93826; D(0)=0.16580
     LONE 2 1 3 3 4 3 6 1 END
     BOND S 1 2 S 2 3 S 2 5 END
   END
   STR        ! Wgt=7.23%; rhoNL=2.93826; D(0)=0.16580
     LONE 2 1 3 3 4 3 5 1 END
     BOND S 1 2 S 2 3 S 2 6 END
   END
   STR        ! Wgt=7.23%; rhoNL=2.93826; D(0)=0.16580
     LONE 2 1 3 3 4 3 5 1 END
     BOND S 1 2 S 2 4 S 2 6 END
   END
   STR        ! Wgt=7.23%; rhoNL=2.93826; D(0)=0.16580
     LONE 1 1 2 1 3 3 4 3 END
     BOND S 2 4 S 2 5 S 2 6 END
   END
   STR        ! Wgt=7.23%; rhoNL=2.93826; D(0)=0.16580
     LONE 1 1 2 1 3 3 4 3 END
     BOND S 2 3 S 2 5 S 2 6 END
   END
 $END

--Link1--
%mem=2gb
%nprocshared=8
%chk=fch3f_m_td
#b3lyp/6-311++g** pop=nbo7read density=current td=(root=73,nstates=100)
```

```
F...CH3F, #p10, IRC=3.53241, E = -239.702977085 (-14.38)
 Excited State  73:      Singlet-A'    12.4289 eV   99.75 nm  f=0.1475  <S**2>=$
       7 -> 18         0.68829
 This state for optimization and/or second-order correction.
 Total Energy, E(TD-HF/TD-DFT) =  -239.246223106 (272.24)

-1 1
                1         -0.049433   -0.000000   -1.030199
                6         -0.384926    0.000000   -0.000022
                9          2.093719    0.000000    0.000027
                9         -1.843073   -0.000000   -0.000008
                1         -0.049418   -0.892157    0.515056
                1         -0.049418    0.892157    0.515056

$nbo file=fch3f_td_p10 archive plot nrt nrtlong $end
 $NRTSTR
   STR        ! Wgt=88.03%; rhoNL=0.10054; D(0)=0.03416
     LONE 3 3 4 4 END
     BOND S 1 2 S 2 3 S 2 5 S 2 6 END
   END
   STR        ! Wgt=8.78%; rhoNL=0.54057; D(0)=0.07890
     LONE 3 4 4 3 END
     BOND S 1 2 S 2 4 S 2 5 S 2 6 END
   END
   STR        ! Wgt=2.93%; rhoNL=1.54547; D(0)=0.13332
     LONE 2 1 3 3 4 3 END
     BOND S 1 2 S 2 5 S 2 6 S 3 4 END
   END
   STR        ! Wgt=53.92%; rhoNL=1.31552; D(0)=0.09401
     LONE 2 1 3 3 4 3 END
     BOND S 1 2 S 2 5 S 2 6 S 3 4 END
   END
   STR        ! Wgt=7.23%; rhoNL=2.93826; D(0)=0.16580
     LONE 2 1 3 3 4 3 6 1 END
     BOND S 1 2 S 2 4 S 2 5 END
   END
   STR        ! Wgt=7.23%; rhoNL=2.93826; D(0)=0.16580
     LONE 2 1 3 3 4 3 6 1 END
     BOND S 1 2 S 2 3 S 2 5 END
   END
   STR        ! Wgt=7.23%; rhoNL=2.93826; D(0)=0.16580
     LONE 2 1 3 3 4 3 5 1 END
     BOND S 1 2 S 2 3 S 2 6 END
   END
   STR        ! Wgt=7.23%; rhoNL=2.93826; D(0)=0.16580
     LONE 2 1 3 3 4 3 5 1 END
     BOND S 1 2 S 2 4 S 2 6 END
   END
   STR        ! Wgt=7.23%; rhoNL=2.93826; D(0)=0.16580
     LONE 1 1 2 1 3 3 4 3 END
     BOND S 2 4 S 2 5 S 2 6 END
   END
   STR        ! Wgt=7.23%; rhoNL=2.93826; D(0)=0.16580
     LONE 1 1 2 1 3 3 4 3 END
     BOND S 2 3 S 2 5 S 2 6 END
   END
 $END

--Link1--
%mem=2gb
%nprocshared=8
%chk=fch3f_m_td
#b3lyp/6-311++g** pop=nbo7read density=current td=(root=78,nstates=100)

F...CH3F, #p11, IRC=3.88657, E = -239.703163738 (-14.49)
 Excited State  78:      Singlet-A'    12.5111 eV   99.10 nm  f=0.1587  <S**2>=$
       7 -> 18         0.69058
 This state for optimization and/or second-order correction.
 Total Energy, E(TD-HF/TD-DFT) =  -239.243389548 (274.02)

-1 1
```

```
                1         -0.066352   -0.000000   -1.029369
                6         -0.406087   -0.000000   -0.000074
                9          2.128121    0.000000    0.000077
                9         -1.861432    0.000000   -0.000015
                1         -0.066277   -0.891378    0.514546
                1         -0.066277    0.891378    0.514546

$nbo file=fch3f_td_p11 archive plot nrt nrtlong $end
 $NRTSTR
   STR        ! Wgt=88.03%; rhoNL=0.10054; D(0)=0.03416
     LONE 3 3 4 4 END
     BOND S 1 2 S 2 3 S 2 5 S 2 6 END
   END
   STR        ! Wgt=8.78%; rhoNL=0.54057; D(0)=0.07890
     LONE 3 4 4 3 END
     BOND S 1 2 S 2 4 S 2 5 S 2 6 END
   END
   STR        ! Wgt=2.93%; rhoNL=1.54547; D(0)=0.13332
     LONE 2 1 3 3 4 3 END
     BOND S 1 2 S 2 5 S 2 6 S 3 4 END
   END
   STR        ! Wgt=53.92%; rhoNL=1.31552; D(0)=0.09401
     LONE 2 1 3 3 4 3 END
     BOND S 1 2 S 2 5 S 2 6 S 3 4 END
   END
   STR        ! Wgt=7.23%; rhoNL=2.93826; D(0)=0.16580
     LONE 2 1 3 3 4 3 6 1 END
     BOND S 1 2 S 2 4 S 2 5 END
   END
   STR        ! Wgt=7.23%; rhoNL=2.93826; D(0)=0.16580
     LONE 2 1 3 3 4 3 6 1 END
     BOND S 1 2 S 2 3 S 2 5 END
   END
   STR        ! Wgt=7.23%; rhoNL=2.93826; D(0)=0.16580
     LONE 2 1 3 3 4 3 5 1 END
     BOND S 1 2 S 2 3 S 2 6 END
   END
   STR        ! Wgt=7.23%; rhoNL=2.93826; D(0)=0.16580
     LONE 2 1 3 3 4 3 5 1 END
     BOND S 1 2 S 2 4 S 2 6 END
   END
   STR        ! Wgt=7.23%; rhoNL=2.93826; D(0)=0.16580
     LONE 1 1 2 1 3 3 4 3 END
     BOND S 2 4 S 2 5 S 2 6 END
   END
   STR        ! Wgt=7.23%; rhoNL=2.93826; D(0)=0.16580
     LONE 1 1 2 1 3 3 4 3 END
     BOND S 2 3 S 2 5 S 2 6 END
   END
 $END
```

Cyanine dyes, $H_2N(CH)_{2n+1}NH_2^+$, *n* = 2-5

```
 %mem=2GB
 %nprocshared=8
 %chk=cyanine_5
 #N B3LYP/6-311++G** POP=NBO7read

 NH2(CH)5NH2(+), E(RB3LYP) =  -305.312767779
 [$CHOOSE selects highest-weighted NRT structure, not NLS structure]
 Excited State   1:      Singlet-A'     4.1112 eV  301.58 nm  f=1.1517  <S**2>=0.000
      26 -> 27         0.71554
      26 <- 27        -0.14117
 This state for optimization and/or second-order correction.
 Total Energy, E(TD-HF/TD-DFT) =  -305.161684554 (94.81)
 E(NLS1) = -304.724414; dE(NLS1) = 0.588354 (369.20 kcal/mol)
 E($del n-ss*) = -305.209627; dE($del n-ss*) = 0.103141 (64.72)
 E2(n-ss*) = 60.86 kcal/mol
 nu: 86,116,211,...,3687
```

```
 Vibrational temperatures:    123.32   166.39   303.57   345.61   422.02
          (Kelvin)             547.79   559.16   727.56   766.63   771.40
                               870.78   888.90   917.70  1240.47  1295.15
                              1372.16  1463.28  1505.25  1509.58  1517.09
                              1757.00  1814.31  1845.60  1913.02  1922.67
                              1958.14  1962.67  2001.23  2258.14  2352.34
                              2367.79  2430.40  2471.70  4502.76  4543.34
                              4548.17  4565.61  4566.07  5133.02  5140.16
                              5304.10  5304.78
 Zero-point correction=                           0.139303 (Hartree/Particle)
 Thermal correction to Energy=                    0.147176
 Thermal correction to Enthalpy=                  0.148121
 Thermal correction to Gibbs Free Energy=         0.107549
 Sum of electronic and zero-point Energies=           -305.173465
 Sum of electronic and thermal Energies=              -305.165591
 Sum of electronic and thermal Enthalpies=            -305.164647
 Sum of electronic and thermal Free Energies=         -305.205219

   1  1
         7       3.639876      -0.131719       0.000000
         6       2.412073       0.373931       0.000000
         6       1.238602      -0.361907       0.000000
         6       0.000000       0.275017       0.000000
         6      -1.238622      -0.362049       0.000000
         6      -2.412175       0.373516       0.000000
         7      -3.639669      -0.132781       0.000000
         1       2.362581       1.459105       0.000000
         1       3.805057      -1.128945       0.000000
         1       4.452221       0.466409       0.000000
         1       1.287014      -1.446719       0.000000
         1      -0.000069       1.364213       0.000000
         1      -1.287354      -1.446865       0.000000
         1      -2.363296       1.458718       0.000000
         1      -4.452529       0.464675       0.000000
         1      -3.804344      -1.130141       0.000000

 $NBO file=cyanine_5 archive nrt plot $END
 $CHOOSE
     LONE 7 1 END
     BOND D 1 2 S 1 9 S 1 10 S 2 3 S 2 8 D 3 4 S 3 11 S 4 5 S 4 12 D 5 6
          S 5 13 S 6 7 S 6 14 S 7 15 S 7 16 END
 $END

--Link1--
%mem=2gb
%nprocshared=8
%chk=cyanine_7
#b3lyp/6-311++g** pop=nbo7read

 cyanine_7 [NH2(CH)7NH2+], E(RB3LYP) =  -382.745785215
 Excited State   1:      Singlet-B2     3.4398 eV  360.44 nm  f=1.5856  <S**2>=0.000
      33 -> 34         0.72066
      33 <- 34        -0.16094
 This state for optimization and/or second-order correction.
 Total Energy, E(TD-HF/TD-DFT) =  -382.619373918 (79.32)
 E(NLS1) = -381.9899642; dE(NLS1) = 0.755821 = 474.29 kcal/mol
 E($del n-ss*) = -382.6474489; dE($del n-ss*) = 0.098336 = 61.71 kcal/mol
 E2(n-ss*) = 57.74
 nu: 52,71,140,...,3696
 Vibrational temperatures:     75.22   102.77   201.06   254.68   269.02
          (Kelvin)             369.11   437.28   467.26   493.30   598.30
                               704.11   704.31   710.58   798.70   872.85
                               881.99   883.52  1234.33  1268.07  1322.23
                              1332.42  1409.69  1469.62  1495.60  1511.56
                              1513.12  1734.96  1782.40  1784.21  1848.50
                              1850.30  1902.85  1910.19  1925.72  1941.95
                              1954.75  2024.88  2232.79  2301.92  2349.74
                              2387.48  2439.87  2454.94  4500.78  4504.43
                              4538.65  4542.34  4546.90  4567.38  4567.44
                              5144.82  5149.96  5317.36  5317.62
 Zero-point correction=                           0.172442 (Hartree/Particle)
```

```
 Thermal correction to Energy=                    0.182550
 Thermal correction to Enthalpy=                  0.183494
 Thermal correction to Gibbs Free Energy=         0.137825
 Sum of electronic and zero-point Energies=           -382.573343
 Sum of electronic and thermal Energies=              -382.563236
 Sum of electronic and thermal Enthalpies=            -382.562292
 Sum of electronic and thermal Free Energies=         -382.607960

+1 1
              7        -0.000000    4.873909    0.076783
              6        -0.000000    3.630334   -0.402089
              6        -0.000000    2.476585    0.358309
              6        -0.000000    1.216347   -0.245247
              6        -0.000000    0.000000    0.428911
              6        -0.000000   -1.216347   -0.245247
              6        -0.000000   -2.476585    0.358309
              6        -0.000000   -3.630334   -0.402089
              7        -0.000000   -4.873909    0.076783
              1        -0.000000    5.060892    1.069479
              1        -0.000000    5.671476   -0.539581
              1        -0.000000    3.556659   -1.485584
              1        -0.000000    2.550714    1.441859
              1        -0.000000    1.184195   -1.333776
              1        -0.000000    0.000000    1.515089
              1        -0.000000   -1.184195   -1.333776
              1        -0.000000   -2.550714    1.441859
              1        -0.000000   -3.556659   -1.485584
              1        -0.000000   -5.060892    1.069479
              1        -0.000000   -5.671476   -0.539581

$nbo file=cyanine_7 archive nrt plot $end

--Link1--
%mem=2gb
%nprocshared=8
%chk=cyanine_7
#b3lyp/6-311++g** pop=nbo7del

 cyanine_7 [NH2(CH)7NH2+], E(RB3LYP) =  -382.745785215
 Excited State   1:      Singlet-B2     3.4398 eV  360.44 nm  f=1.5856  <S**2>=0.000
      33 -> 34         0.72066
      33 <- 34        -0.16094
 This state for optimization and/or second-order correction.
 Total Energy, E(TD-HF/TD-DFT) =  -382.619373918 (79.32)
 nu: 52,71,140,...,3696
 Vibrational temperatures:     75.22   102.77   201.06   254.68   269.02
          (Kelvin)           369.11   437.28   467.26   493.30   598.30
                             704.11   704.31   710.58   798.70   872.85
                             881.99   883.52  1234.33  1268.07  1322.23
                            1332.42  1409.69  1469.62  1495.60  1511.56
                            1513.12  1734.96  1782.40  1784.21  1848.50
                            1850.30  1902.85  1910.19  1925.72  1941.95
                            1954.75  2024.88  2232.79  2301.92  2349.74
                            2387.48  2439.87  2454.94  4500.78  4504.43
                            4538.65  4542.34  4546.90  4567.38  4567.44
                            5144.82  5149.96  5317.36  5317.62
 Zero-point correction=                           0.172442 (Hartree/Particle)
 Thermal correction to Energy=                    0.182550
 Thermal correction to Enthalpy=                  0.183494
 Thermal correction to Gibbs Free Energy=         0.137825
 Sum of electronic and zero-point Energies=           -382.573343
 Sum of electronic and thermal Energies=              -382.563236
 Sum of electronic and thermal Enthalpies=            -382.562292
 Sum of electronic and thermal Free Energies=         -382.607960

+1 1
              7        -0.000000    4.873909    0.076783
              6        -0.000000    3.630334   -0.402089
              6        -0.000000    2.476585    0.358309
              6        -0.000000    1.216347   -0.245247
              6        -0.000000    0.000000    0.428911
```

```
              6         -0.000000   -1.216347   -0.245247
              6         -0.000000   -2.476585    0.358309
              6         -0.000000   -3.630334   -0.402089
              7         -0.000000   -4.873909    0.076783
              1         -0.000000    5.060892    1.069479
              1         -0.000000    5.671476   -0.539581
              1         -0.000000    3.556659   -1.485584
              1         -0.000000    2.550714    1.441859
              1         -0.000000    1.184195   -1.333776
              1         -0.000000    0.000000    1.515089
              1         -0.000000   -1.184195   -1.333776
              1         -0.000000   -2.550714    1.441859
              1         -0.000000   -3.556659   -1.485584
              1         -0.000000   -5.060892    1.069479
              1         -0.000000   -5.671476   -0.539581

$nbo file=cyanine_7 archive nrt plot $end
$CHOOSE
     LONE 9 1 END
     BOND D 1 2 S 1 10 S 1 11 S 2 3 S 2 12 D 3 4 S 3 13 S 4 5 S 4 14 D 5 6
          S 5 15 S 6 7 S 6 16 D 7 8 S 7 17 S 8 9 S 8 18 S 9 19 S 9 20 END
$END
$DEL
 lewis
 delete 1 element 10 51
$END

--Link1--
%mem=2gb
%nprocshared=8
%chk=cyanine_9
#b3lyp/6-311++g** pop=nbo7read

 cyanine_9 [NH2(CH)9NH2+], E(RB3LYP) =  -460.177695895
 nu: 36,49,96,...,3702
 Vibrational temperatures:     51.61    69.92   138.23   185.91   205.17
          (Kelvin)            260.27   343.26   368.59   389.21   409.07
                              527.56   530.96   615.13   649.26   651.63
                              682.33   717.43   855.73   858.32   871.14
                              880.45  1231.22  1255.18  1296.16  1313.86
                             1335.92  1372.72  1430.60  1475.77  1499.75
                             1501.81  1513.84  1729.60  1734.90  1759.81
                             1806.42  1824.22  1857.95  1863.08  1895.67
                             1903.24  1916.16  1924.68  1940.16  1948.53
                             2040.98  2207.34  2272.52  2326.49  2373.28
                             2390.35  2440.82  2441.49  4496.72  4499.62
                             4502.27  4531.67  4535.54  4539.05  4542.11
                             4566.34  4566.39  5153.48  5157.47  5326.58
                             5326.77
 Zero-point correction=                           0.205573 (Hartree/Particle)
 Thermal correction to Energy=                    0.217920
 Thermal correction to Enthalpy=                  0.218865
 Thermal correction to Gibbs Free Energy=         0.167472
 Sum of electronic and zero-point Energies=           -459.972123
 Sum of electronic and thermal Energies=              -459.959775
 Sum of electronic and thermal Enthalpies=            -459.958831
 Sum of electronic and thermal Free Energies=         -460.010224

   1  1
  N
  C     1  r2
  C     2  r3        1  a3
  C     3  r4        2  a4        1  d4
  C     4  r5        3  a5        2  d5
  C     5  r6        4  a6        3  d6
  C     6  r7        5  a7        4  d7
  C     7  r8        6  a8        5  d8
  C     8  r9        7  a9        6  d9
  C     9  r10       8  a10       7  d10
  N    10  r11       9  a11       8  d11
  H     1  r12       2  a12       3  d12
```

```
 H    11  r13       10  a13        9  d13
 H     1  r14        2  a14        3  d14
 H    11  r15       10  a15        9  d15
 H     2  r16        1  a16       12  d16
 H    10  r17       11  a17       13  d17
 H     3  r18        2  a18        1  d18
 H     9  r19       10  a19       11  d19
 H     4  r20        3  a20        2  d20
 H     8  r21        9  a21       10  d21
 H     5  r22        4  a22        3  d22
 H     7  r23        8  a23        9  d23
 H     6  r24        5  a24        4  d24

r2       1.3363
r3       1.3791
a3     125.6225
r4       1.4008
a4     121.2708
d4     180.0000
r5       1.3877
a5     125.4002
d5     180.0000
r6       1.3948
a6     122.3467
d6     180.0000
r7       1.3948
a7     125.2333
d7     180.0000
r8       1.3877
a8     122.3467
d8     180.0000
r9       1.4008
a9     125.4002
d9     180.0000
r10      1.3791
a10    121.2708
d10    180.0000
r11      1.3363
a11    125.6225
d11    180.0000
r12      1.0096
a12    121.6556
d12      0.0000
r13      1.0096
a13    121.6556
d13      0.0000
r14      1.0074
a14    121.2474
d14    180.0000
r15      1.0074
a15    121.2474
d15    180.0000
r16      1.0859
a16    114.8532
d16    180.0000
r17      1.0859
a17    114.8532
d17    180.0000
r18      1.0864
a18    119.4128
d18      0.0000
r19      1.0864
a19    119.4128
d19      0.0000
r20      1.0891
a20    117.2419
d20      0.0000
r21      1.0891
a21    117.2419
d21      0.0000
r22      1.0866
```

```
 a22    118.9214
 d22      0.0000
 r23      1.0866
 a23    118.9214
 d23      0.0000
 r24      1.0891
 a24    117.3834
 d24      0.0000

$nbo file=cyanine_9 archive nrt plot $end
$CHOOSE
  LONE 1 1 END
  BOND S 1 12  S 1 14  S 2 16  S 3 18  S 4 20  S 5 22
       S 6 24  S 7 23  S 8 21  S 9 19  S 10 17  S 11 13
       S 11 15
       S 1 2  D 2 3  S 3 4  D 4 5  S 5 6  D 6 7  S 7 8
       D 8 9  S 9 10  D 10 11 END
$END

--Link1--
%mem=2gb
%nprocshared=8
%chk=cyanine_9
#b3lyp/6-311++g** pop=nbo7del

 cyanine_9 [NH2(CH)9NH2+], E(RB3LYP) =  -460.177695895
 E(NLS) = ; dE(NLS) =  kcal/mol
 E($del 12-45) = ; dE($del nN-piCC*) = 59.88
 E($del 17-50) = ; dE($del piCC-piCC*) = 33.80
 E($del 22-55) = ; dE($del piCC-piCC*) = 31.09
 E($del 27-60) = ; dE($del piCC-piCC*) = 33.39
 E($del 32-65) = ; dE($del piCC-piCN*) = 64.99
 E2(n-ss*) =

   1  1
  N
  C     1  r2
  C     2  r3        1  a3
  C     3  r4        2  a4        1  d4
  C     4  r5        3  a5        2  d5
  C     5  r6        4  a6        3  d6
  C     6  r7        5  a7        4  d7
  C     7  r8        6  a8        5  d8
  C     8  r9        7  a9        6  d9
  C     9  r10       8  a10       7  d10
  N    10  r11       9  a11       8  d11
  H     1  r12       2  a12       3  d12
  H    11  r13      10  a13       9  d13
  H     1  r14       2  a14       3  d14
  H    11  r15      10  a15       9  d15
  H     2  r16       1  a16      12  d16
  H    10  r17      11  a17      13  d17
  H     3  r18       2  a18       1  d18
  H     9  r19      10  a19      11  d19
  H     4  r20       3  a20       2  d20
  H     8  r21       9  a21      10  d21
  H     5  r22       4  a22       3  d22
  H     7  r23       8  a23       9  d23
  H     6  r24       5  a24       4  d24

 r2       1.3363
 r3       1.3791
 a3     125.6225
 r4       1.4008
 a4     121.2708
 d4     180.0000
 r5       1.3877
 a5     125.4002
 d5     180.0000
 r6       1.3948
 a6     122.3467
```

```
 d6     180.0000
 r7       1.3948
 a7     125.2333
 d7     180.0000
 r8       1.3877
 a8     122.3467
 d8     180.0000
 r9       1.4008
 a9     125.4002
 d9     180.0000
 r10      1.3791
 a10    121.2708
 d10    180.0000
 r11      1.3363
 a11    125.6225
 d11    180.0000
 r12      1.0096
 a12    121.6556
 d12      0.0000
 r13      1.0096
 a13    121.6556
 d13      0.0000
 r14      1.0074
 a14    121.2474
 d14    180.0000
 r15      1.0074
 a15    121.2474
 d15    180.0000
 r16      1.0859
 a16    114.8532
 d16    180.0000
 r17      1.0859
 a17    114.8532
 d17    180.0000
 r18      1.0864
 a18    119.4128
 d18      0.0000
 r19      1.0864
 a19    119.4128
 d19      0.0000
 r20      1.0891
 a20    117.2419
 d20      0.0000
 r21      1.0891
 a21    117.2419
 d21      0.0000
 r22      1.0866
 a22    118.9214
 d22      0.0000
 r23      1.0866
 a23    118.9214
 d23      0.0000
 r24      1.0891
 a24    117.3834
 d24      0.0000

$nbo file=cyanine_9 archive nrt plot $end
$CHOOSE
  LONE 1 1 END
  BOND S 1 12  S 1 14  S 2 16  S 3 18  S 4 20  S 5 22
       S 6 24  S 7 23  S 8 21  S 9 19  S 10 17  S 11 13
       S 11 15
       S 1 2  D 2 3  S 3 4  D 4 5  S 5 6  D 6 7  S 7 8
       D 8 9  S 9 10  D 10 11 END
$END
$DEL
 DELETE 1 ELEMENT 12 45
 DELETE 1 ELEMENT 17 50
 DELETE 1 ELEMENT 22 55
 DELETE 1 ELEMENT 27 60
 DELETE 1 ELEMENT 32 65
```

```
 LEWIS
$END

--Link1--
%mem=2gb
%nprocshared=8
%chk=cyanine_11
#b3lyp/6-311++g** pop=nbo7read

 cyanine_11 [NH2(CH)11NH2+], E(RB3LYP) =  -537.608866925
 nu: 26,35,69,...,3707
 Vibrational temperatures:     37.46    50.20    99.70   135.49   171.65
          (Kelvin)            192.95   255.43   311.63   313.60   330.79
                              401.35   433.83   462.76   558.92   561.48
                              593.55   598.51   605.74   627.49   719.64
                              804.29   840.43   841.68   862.64   903.13
                             1228.48  1244.36  1278.73  1300.94  1312.69
                             1341.27  1347.87  1398.79  1443.80  1481.74
                             1497.75  1500.00  1514.80  1722.24  1723.58
                             1738.66  1751.89  1805.90  1832.68  1833.50
                             1863.73  1870.15  1892.56  1894.71  1910.60
                             1915.81  1927.71  1930.82  1959.84  2050.44
                             2195.05  2240.16  2308.77  2360.49  2376.63
                             2384.54  2436.45  2438.97  4495.49  4497.04
                             4499.28  4501.94  4527.15  4530.90  4534.04
                             4537.16  4539.31  4564.77  4565.57  5160.34
                             5163.63  5333.92  5334.09
 Zero-point correction=                           0.238708 (Hartree/Particle)
 Thermal correction to Energy=                    0.253301
 Thermal correction to Enthalpy=                  0.254245
 Thermal correction to Gibbs Free Energy=         0.196457
 Sum of electronic and zero-point Energies=           -537.370159
 Sum of electronic and thermal Energies=              -537.355566
 Sum of electronic and thermal Enthalpies=            -537.354622
 Sum of electronic and thermal Free Energies=         -537.412410

   1  1
            7         -7.338294    0.060711   -0.000000
            6         -6.072364    0.497510   -0.000000
            6         -4.948554   -0.297526   -0.000000
            6         -3.659329    0.257532   -0.000000
            6         -2.475833   -0.462153    0.000000
            6         -1.220977    0.154238   -0.000000
            6          0.000000   -0.513579    0.000000
            6          1.221101    0.154251   -0.000000
            6          2.475837   -0.462183    0.000000
            6          3.659436    0.257524    0.000000
            6          4.948493   -0.297545    0.000000
            6          6.072353    0.497586   -0.000000
            7          7.338141    0.060598    0.000000
            1         -7.557956   -0.924298    0.000000
            1         -8.112840    0.704225   -0.000000
            1         -5.963729    1.577995   -0.000000
            1         -5.060047   -1.378339    0.000000
            1         -3.585427    1.344137   -0.000000
            1         -2.521479   -1.547966    0.000000
            1         -1.197979    1.243069   -0.000000
            1          0.000074   -1.600465    0.000000
            1          1.197955    1.243085   -0.000000
            1          2.521574   -1.547992    0.000000
            1          3.585386    1.344098   -0.000000
            1          5.060183   -1.378258    0.000000
            1          5.963690    1.578011   -0.000000
            1          7.557829   -0.924332    0.000000
            1          8.112870    0.703935   -0.000000

$nbo file=cyanine_11 archive nrt plot $end

--Link1--
%mem=2gb
%nprocshared=8
```

```
%chk=cyanine_11
#b3lyp/6-311++g** pop=nbo7del

 cyanine_11 [NH2(CH)11NH2+], E(RB3LYP) =  -537.608866925
 E(NLS) = ; dE(NLS) =  kcal/mol
 E($del 14 75); dE($del nN-piCC*) =
 E2(14-75) = 54.34
 E($del 42,70); dE($del piCC-piCC*) =
 E2(42-70) = 27.95
 E($del 37-65); dE($del piCC-piCC*) =
 E2(37-65) = 27.20
 E($del 32-60); dE($del piCC-piCC*) =
 E2(32-60) = 29.00
 E($del 27-55); dE($del piCC-piCC*) =
 E2(27-55) = 35.87
 E($del 22-49); dE($del piCC-piCN*) =
 E2(22-49) = 62.88

   1  1
             7         -7.338294    0.060711   -0.000000
             6         -6.072364    0.497510   -0.000000
             6         -4.948554   -0.297526   -0.000000
             6         -3.659329    0.257532   -0.000000
             6         -2.475833   -0.462153    0.000000
             6         -1.220977    0.154238   -0.000000
             6          0.000000   -0.513579    0.000000
             6          1.221101    0.154251   -0.000000
             6          2.475837   -0.462183    0.000000
             6          3.659436    0.257524    0.000000
             6          4.948493   -0.297545    0.000000
             6          6.072353    0.497586   -0.000000
             7          7.338141    0.060598    0.000000
             1         -7.557956   -0.924298    0.000000
             1         -8.112840    0.704225   -0.000000
             1         -5.963729    1.577995   -0.000000
             1         -5.060047   -1.378339    0.000000
             1         -3.585427    1.344137   -0.000000
             1         -2.521479   -1.547966    0.000000
             1         -1.197979    1.243069   -0.000000
             1          0.000074   -1.600465    0.000000
             1          1.197955    1.243085   -0.000000
             1          2.521574   -1.547992    0.000000
             1          3.585386    1.344098   -0.000000
             1          5.060183   -1.378258    0.000000
             1          5.963690    1.578011   -0.000000
             1          7.557829   -0.924332    0.000000
             1          8.112870    0.703935   -0.000000

$nbo file=cyanine_11 archive nrt plot $end
$CHOOSE
     LONE 13 1 END
     BOND D 1 2 S 1 14 S 1 15 S 2 3 S 2 16 D 3 4 S 3 17 S 4 5 S 4 18 D 5 6
          S 5 19 S 6 7 S 6 20 D 7 8 S 7 21 S 8 9 S 8 22 D 9 10 S 9 23 S 10 11
          S 10 24 D 11 12 S 11 25 S 12 13 S 12 26 S 13 27 S 13 28 END
$END
$DEL
 DELETE 1 ELEMENT 14 75
 DELETE 1 ELEMENT 42 70
 DELETE 1 ELEMENT 37 65
 DELETE 1 ELEMENT 32 60
 DELETE 1 ELEMENT 27 55
 DELETE 1 ELEMENT 22 49
 LEWIS
$END

--Link1--
```
